# Comparative analysis of photocatalytic, enzymatic, and chemical approaches to identify canonical and noncanonical surfaceomes

**DOI:** 10.1073/pnas.2623700123

**Published:** 2026-10-21

**Authors:** William W. Ho, Yifei Chen, Tomasz Slezak, Oleksandr Zginnyk, Trenton M. Peters-Clarke, Kaitlin Schaefer, Anthony A. Kossiakoff, Kevin K. Leung, James A. Wells

**Affiliations:** ^a^ Department of Pharmaceutical Chemistry, University of California, San Francisco, CA 94158; ^b^ Department of Biochemistry and Molecular Biology, The University of Chicago, Chicago, IL 60637; ^c^ Department of Microbial Pathogenesis, Yale University School of Medicine, New Haven, CT 06519; ^d^ Institute for Biophysical Dynamics, The University of Chicago, Chicago, IL 60637; ^e^ Department of Cellular and Molecular Pharmacology, University of California, San Francisco, CA 94158

**Keywords:** surfaceomics, proxmity labeling, photocatalysis, membrane proteins, cell surface

## Abstract

The cell surface is a critical nexus in cellular communication and a rich landscape yielding the majority of current therapeutic targets, yet only a small fraction of cell surface proteins have been targeted therapeutically. The cell surface proteome (surfaceome) comprises only ~1% of the human proteome by mass, motivating enrichment strategies to detect surfaceome alterations in disease for drug target discovery. Developments in photocatalytic technology have revolutionized the study of protein neighborhoods around specific targets of interest, but photocatalysis has been underutilized for global surfaceome analysis. Here, we describe five distinct photocatalytic surfaceomics strategies, all without the need for cellular engineering, that apply the photocatalyst Eosin Y attached to different cell surface structures: direct glycan ligation, glycan attachment via two different lectins, membrane insertion, and free in extracellular space. We systematically compare these photocatalytic surfaceomics methods to each other as well as to three established chemical and enzymatic surfaceomics methods. We demonstrate that the different photocatalytic surfaceomics techniques achieve broadly similar canonical cell surface protein enrichment but show some important differences reflective of cell surface attachment mechanism. These mechanistically orthogonal surfaceomics techniques consistently identified numerous nonglycosylated proteins with high concordance, many of which represent an underappreciated class of noncanonical cell surface proteins lacking traditional cell surface protein features that may represent potential drug targets. This study substantially expands options for surfaceomics methods using photocatalytic proximity labeling.

## Significance

The cell surface proteome is an important area of study for disease biology and intervention. Existing surfaceomics strategies based on small molecule chemistry have limited detection scope and poor temporal control, while those based on enzymatic radical generators induce oxidative stress at biologic temperatures where the surfaceome can remodel. Photocatalysis, akin to molecular photography, has emerged as a promising tool in proximity labeling proteomics but has yet to be applied broadly for unbiased surfaceomics. Here, we employ protein-agnostic photocatalysis to achieve high resolution, minimally perturbing snapshots of the cell surface. By systematic comparison of eight surfaceomics methods, we uncover large numbers of nonglycosylated and noncanonical cell surface proteins that comprise an emerging class of surface localized proteins.

The cell surface is an essential interface for communication between a cell and its surrounding environment. Cell surface proteins (CSPs), which are commonly glycosylated (1) and densely packed within the plasma membrane (PM) (2), contribute more than 50% of FDA-approved small molecules and virtually all the biologics (3, 4). However, the estimated ~5,000 canonical CSPs (cCSPs) comprise only ~1% of the cellular proteome by mass (5), challenging dynamic CSP quantitation by whole cell proteomics (WCP) and motivating enrichment tools for the systems-level study of the cell surface proteome (surfaceomics) (for recent review see ref. 5).

As physical isolation of PM proteins is challenging (6–10), surfaceomics techniques frequently tag CSPs with a biotin enrichment handle (11–18). Published surfaceomics techniques differ mechanistically in target coverage and specificity (5). Established techniques include the gold standard chemical glycan-biotinylating cell surface capture (CSC) (14–16, 19, 20), enzymatic tyrosine biotinylation via radicals generated by enzymes like the glycan-tethered lectin-enzyme conjugate wheat germ agglutinin-horseradish peroxidase (WGA-HRP) (12, 19, 21, 22) or conjugates of APEX2 (11, 13, 23), and chemical amine biotinylation by sulfo-N-hydroxysuccinimide ester biotin (Sulfo-NHS) (17–19, 24). Limited direct comparisons of existing surfaceomics methods show low correlations of protein detection (13) that could be attributable to differences in labeling chemistry or localization to distinct microenvironments of the heterogeneous cell surface (25–27).

Photocatalytic biotinylation has emerged as a promising technology for proximity labeling proteomics (PLP) to study interacting protein neighborhoods (25, 28–33), but the application of photocatalytic PLP (photo-PLP) to the whole surfaceome has been limited (32, 34). In this study, we bridge photo-PLP and surfaceomics via five photo-PLP surfaceomics strategies that localize a standardized photo-PLP photocatalyst, Eosin Y (EY), to various molecular constituents of the cell surface and extracellular space. Under the control of visible light, EY activates diffusive biotin-probes (31) that rapidly insert into proximal biomolecules (32). We validate the cell surface specificity of these photo-PLP surfaceomics techniques and compare them alongside CSC, WGA-HRP, and Sulfo-NHS. Since the diffusive nature of photo-PLP surfaceomics labeling is not restricted by protein glycosylation as the gold standard CSC method is, we consistently recovered many nonglycosylated and noncanonical CSPs (nCSPs) that corroborate and expand the growing set of recently discovered nCSPs (23, 34, 35). We believe this study will provide an important resource for the surfaceomics and drug discovery communities for investigating canonical and noncanonical extracellular proteins.

## Results

### Photo-PLP Surfaceomics Design.

**Fig. 1.**
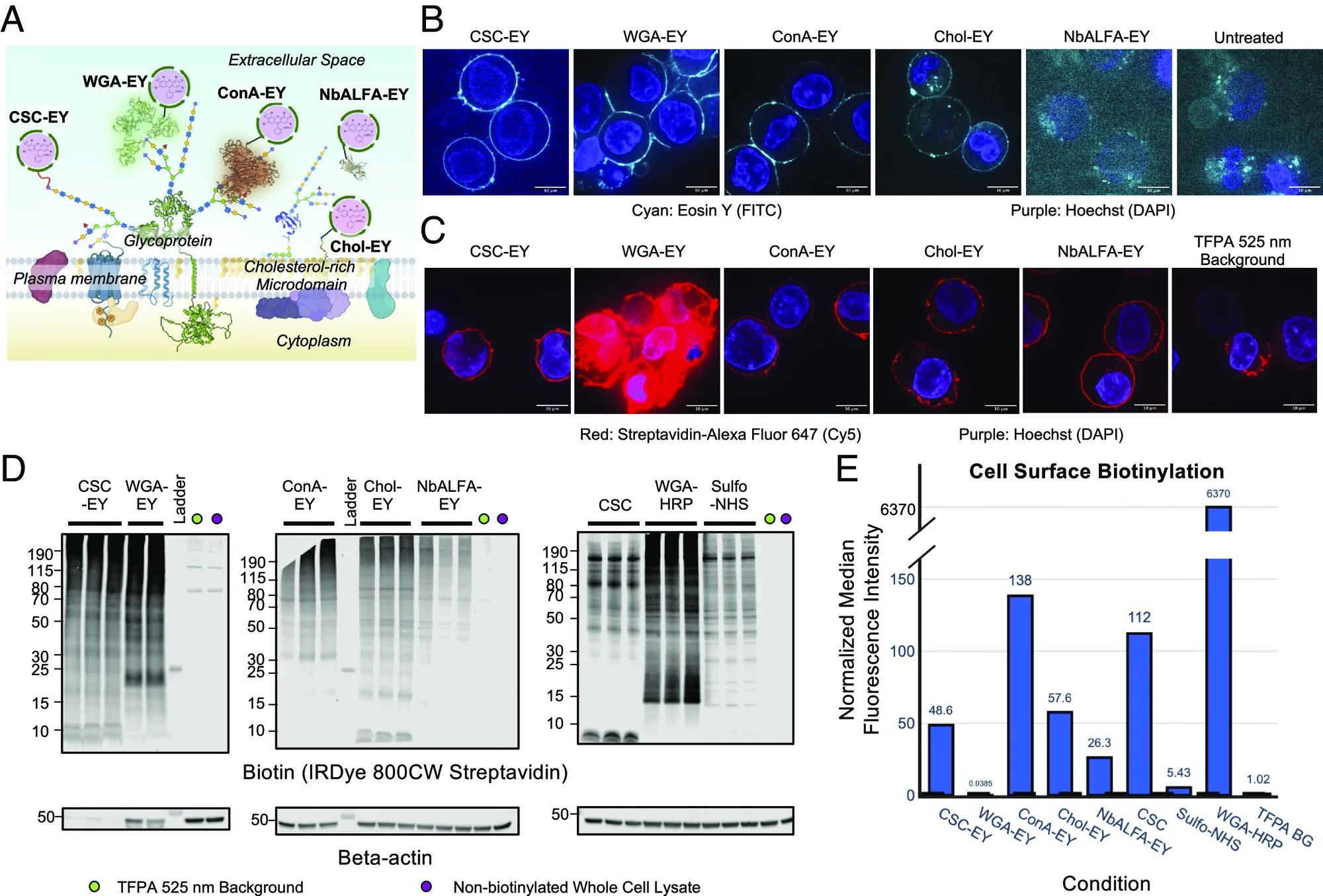
Development of five photo-PLP surfaceomics techniques. (*A*) Photo-PLP surfaceomics techniques localize EY to the cell surface or extracellular space. CSC-EY and the lectin conjugates WGA-EY and ConA-EY attach EY to glycans. Chol-EY embeds EY in the PM. Soluble, inert NbALFA-EY does not bind to the PM or internalize. (*B*) Photo-PLP conjugates localize to the cell surface, with only nontethered NbALFA-EY not showing appreciable cell surface binding as expected. (Scale bar 10 μm.) (*C*) Biotinylation by photo-PLP surfaceomics techniques is restricted to the cell surface. (Scale bar 10 μm.) (*D*) All surfaceomics techniques biotinylate proteins but have subtle differences. (*E*) Flow cytometry of nonpermeabilized cells affirms successful cell surface biotinylation by photo-PLP surfaceomics methods. Median fluorescence intensity of Streptavidin-Alexa Fluor 647 signal was normalized to that of nonbiotinylated cells incubated with the same fluorescent probe in the same experimental batch.

To interrogate the surfaceome, we developed five strategies to localize the same photo-PLP photocatalyst to the cell surface (Fig. 1*A*). We chose the commercially available photocatalyst Eosin Y (EY) for its versatility and accessibility (31). We designed all photo-PLP surfaceomics methods with 4 °C labeling compatibility to minimize internalization, signaling, and membrane reorganization (36–39). These EY-conjugates leverage cell surface enrichment principles currently exploited for CSP enrichment and nonphotocatalytic surfaceomics.

We first employed a photo-PLP approach (CSC-EY) that attaches EY to glycans which are the targets of CSC (14–16). For this, we synthesized an aminooxy-EY small molecule to react with sodium periodate-oxidized cell surface glycans (*SI Appendix*, Fig. S1). Next, we used wheat germ agglutinin (WGA) that binds sialic acid and N-acetylglucosamine (GlcNAc) (40–42) and is frequently employed for surfaceomics and staining PM glycoproteins (43, 44). We conjugated EY onto WGA to generate the photo-PLP tool WGA-EY (*SI Appendix*, Fig. S2). We next generated ConA-EY, a conjugate of EY with the lectin concanavalin A (ConA) (*SI Appendix*, Fig. S3) that is widely used for glycoprotein enrichment (45) via mannose and glucose binding (46, 47). Next, we adapted the lipid cholesterol, which has recently been used as a surfaceomics method with diffusive APEX2-mediated Tyr biotinylation (11) and POCA for oxidation of His residues into reactive electrophiles (48), into Chol-EY (*SI Appendix*, Fig. S4). As a lipid, Chol-EY was first incorporated into the PM by a 5-min incubation with cells at 37 °C before cells were cooled to 4 °C. Last, we conjugated EY to a nonbinding nanobody, NbALFA (49) (*SI Appendix*, Fig. S5), that in the absence of an engineered ALFA-tag target restricts EY to the extracellular space akin to APEX2-based SLAPSHOT (13). We chose NbALFA-EY over free EY small molecule because free EY can enter cells (33) or adsorb onto the PM nonspecifically (50).

### Comparisons of Labeling Patterns at the Cellular Level.

All experiments were performed on wildtype K562 chronic myelogenous leukemia cells. We confirmed that the EY conjugates localized as intended by leveraging the fluorescence of EY (51) in confocal microscopy (Fig. 1*B*). As expected, all EY conjugates except NbALFA-EY bound to the cell surface. Most showed broadly uniform cell surface localization across single cells with two exceptions: WGA-EY and Chol-EY. WGA-EY induced cellular aggregation consistent with the known cellular agglutination activity of WGA on suspended cells (40, 52). Chol-EY showed nonuniform cell surface distribution, which may be due to heterogeneous distribution of cholesterol across subdomains of the plasma membrane like lipid rafts. Chol-EY also localized in intracellular puncta that likely reflect mild internalization before the cells cooled to 4 °C (*SI Appendix*, Fig. S6). In NbALFA-EY and the untreated control, we did not observe notable cell surface signal, but we noted intracellular puncta in the EY channel that we attribute to cellular autofluorescence (53) because they align with paraformaldehyde fixation-induced blebs (54) in brightfield (*SI Appendix*, Fig. S7). The aminooxy-EY used in CSC-EY bound cells without periodate oxidation (*SI Appendix*, Fig. S8), possibly because of uptake of the small molecule (55, 56), nonspecific adsorption to lipids (50), baseline oxidation of CSPs, or bond formation between cell surface carboxylates and a carbodiimide generated via oxidative desulfurization of the molecule’s thiourea linker. This highlights the disadvantage of using small molecule EY compared to larger conjugates. Nonetheless, there was an appreciable difference in aminooxy-EY attachment between the periodate oxidized and unoxidized conditions at 50 μM; thus, this concentration was employed in CSC-EY.

We biotinylated the cell surface via the EY-catalyzed conversion of the tetrafluorophenyl azide (TFPA)-biotin photoprobe into a diffusive aryl nitrene-biotin reactive intermediate (31). While the original report on EY for targeted photo-PLP used 450 nm blue light to activate the photocatalyst (31), blue light independently converts TFPA-biotin into the reactive intermediate and contributes background labeling (57, 58). To reduce this, we utilized a green light-emitting diode (LED) (hv_max_ = 520 to 525 nm) that does not appreciably activate TFPA in the absence of EY and aligns better with the maximum excitation wavelength of EY [λ_max_^ex^ = 515 nm (59)]. Confocal microscopy confirmed that biotinylation was spatially constrained to the cell surface (Fig. 1*C*). Consistent with the EY localization microscopy, we noted nonuniform surface distribution of Chol-EY biotinylation with cell surface and intracellular puncta consistent with microdomain localization and mild internalization, aggregation of WGA-EY treated cells, and surface biotinylation by aminooxy-EY without periodate oxidation (*SI Appendix*, Fig. S9). After irradiation, the EY signal was not ablated and showed similar patterns to biotinylation signal (*SI Appendix*, Fig. S10). NbALFA-EY displayed very faint cell surface signal in the EY channel, which may reflect a small amount of NbALFA-EY adsorbing or being crosslinked to the cell surface (*Discussion*).

As a comparator, we performed the same surface biotinylation assessment on established surfaceomics methods CSC, WGA-HRP, and Sulfo-NHS (*SI Appendix*, Fig. S11). The small molecule method CSC demonstrated exquisite cell surface selectivity and healthy cell morphology, as did Sulfo-NHS. In contrast, enzymatic WGA-HRP demonstrated poor cell recovery, substantial heterogeneity in cell surface labeling, and damaged cell integrity. Nevertheless, the biotin signal from WGA-HRP was still predominantly cell surface localized.

Western blot confirmed successful biotinylation by all surfaceomics methods (Fig. 1*D*) but revealed differences in biotinylation patterns. This was most pronounced in the lower molecular weight (MW) range, where CSC-EY, WGA-EY, WGA-HRP, and Sulfo-NHS biotinylated a broad range of proteins down to below 15 kDa. In contrast, ConA-EY, NbALFA-EY, and CSC demonstrated a higher MW preference with a lower bound at ~30 kDa. Chol-EY exhibited a similar high MW preference but also mild biotinylation down to ~15 kDa; in this and CSC, we also noted the robust biotinylation of a distinct low MW species. These MW differences were unexpected and may reflect different molecular target biases (*Discussion*).

We quantified the degree of cell surface biotinylation using flow cytometry. For all photo-PLP methods except WGA-EY, there were at least 10-fold increases in median cell surface biotin signal over the nonbiotinylated control (Fig. 1*E*). Because we mechanically strained and gated for single cells, WGA-EY as a cellular aggregator is not expected to perform well by this assay. WGA-HRP behaved quite differently from WGA-EY and did recover biotinylated single cells. This likely results from protocol differences between WGA-HRP (12) and WGA-EY: WGA-HRP is not allowed to incubate with rotation, reducing agglutination at the expense of uniform coating. Another rationalization is that the higher efficiency and labeling radius of enzymatic HRP may heavily biotinylate lightly coated single cells. Fixation improved cell recovery of photo-PLP and enzymatically labeled cells for flow cytometry (*SI Appendix*, Fig. S12), potentially by restoring structural integrity that is damaged by a combination of oxidative stress thinning the PM (50) and tensile force from centrifuging agglutinized cells, particularly for the multivalent lectin-based methods. This cell pellet decline was apparent after extended flow cytometry incubations and washes but did not impact proteomics samples as appreciably. We also noted that although aminooxy-EY alone performed cell surface biotinylation, this was an order of magnitude less than CSC-EY (*SI Appendix*, Fig. S13).

### Proteomics Experimental Design for Standardized Comparison of Surfaceomics Techniques.

**Fig. 2.**
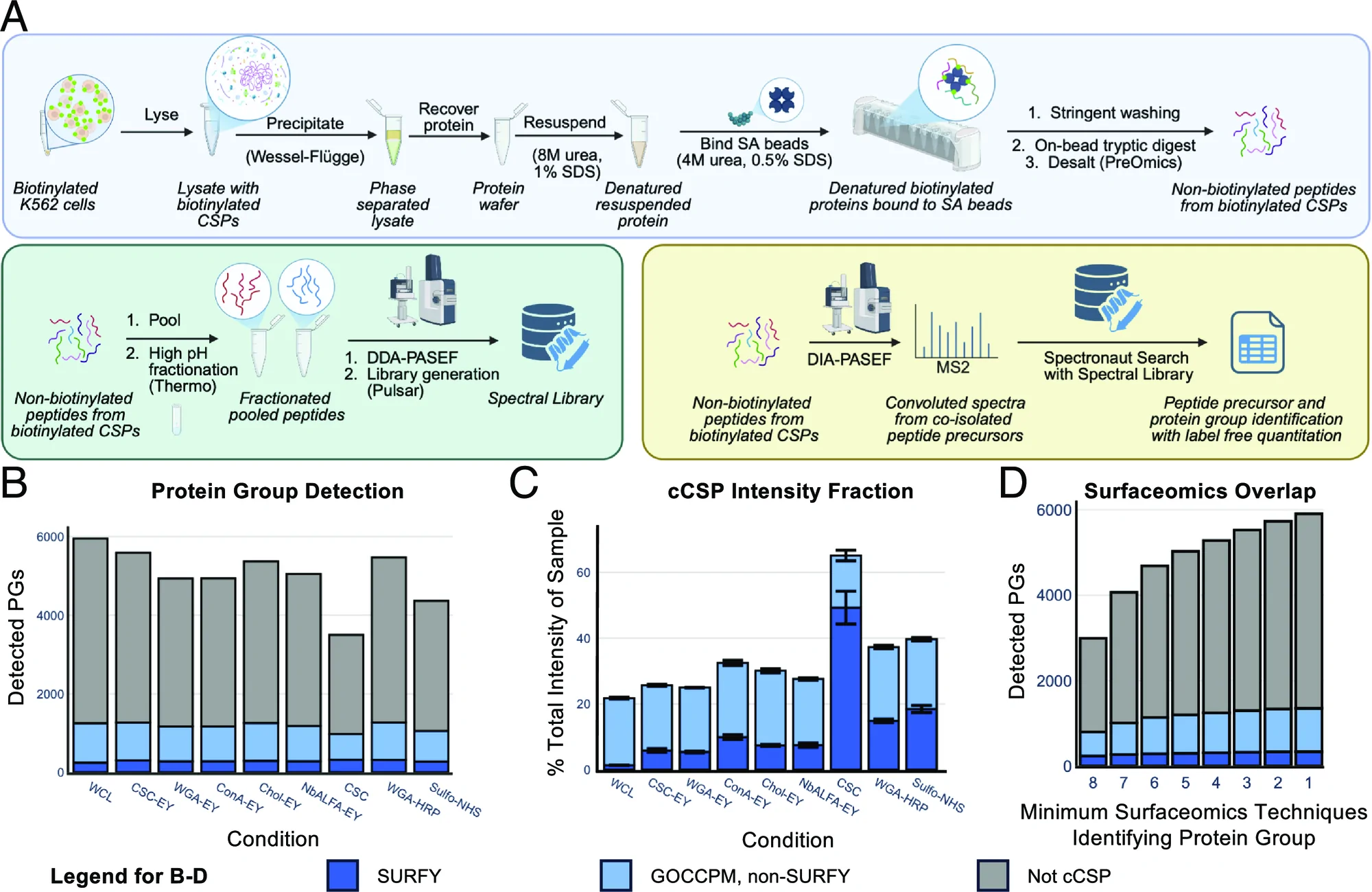
Surfaceomics achieves high confidence detection of cell surface proteins. (*A*) Workflow for preparation of peptides from biotinylated proteins and mass spectrometry. Figure created with Biorender.com. (*B*) Protein group detection counts, annotated for cCSPs. (*C*) Fraction of total LFQ intensity within each sample contributed by cCSPs. Data are mean of biological triplicates with SD shown as error bars. (*D*) Analysis by the number of protein groups consistently detected across at least *n* surfaceomics samples demonstrates high overlap in enriched proteins across surfaceomics methods.

Having compared the breadth, effectiveness, and selectivity of cell surface biotinylation via conventional biochemical assays, we next assessed the surfaceomics methods by bottom–up enrichment proteomics. For higher confidence, we performed data independent acquisition-parallel accumulation-serial fragmentation [DIA-PASEF (60, 61)] with an offline fractionated data-dependent acquisition-parallel accumulation-serial fragmentation [DDA-PASEF (62)]-generated spectral library (Fig. 2*A*). Nonbiotinylated K562 cells were prepared as whole cell lysate (WCL) for a WCP comparison. Two technical negative controls were also included. The bead background (bead BG) reflects nonbiotinylated lysate treated as if it were a surfaceomics sample. This bead BG, in which the only biotinylated proteins are endogenously biotinylated, was used to gauge nonspecific binding to the streptavidin enrichment beads. The TFPA background (TFPA BG) reflects a no EY control where cells were not treated with EY but with TFPA-biotin and green light irradiation, capturing residual biotinylation promoted by light as well as adsorption to the beads.

Biotin enrichment was conducted at the protein level for all samples, including for CSC where glycopeptide enrichment is the norm (14, 16). We performed protein-level enrichment to control for technical differences of protein vs. peptide enrichment: Protein-level enrichment is more prone to nonspecific binding of protein to the enrichment support, and a recent study comparing WGA-HRP with protein-level enrichment to CSC with peptide-level enrichment suggested that CSC may achieve greater surface specificity in part because it is a peptide-level enrichment method (19). Consistent with these observations, when we performed protein-level biotin enrichment on nonbiotinylated whole cell lysate by a previously used protocol (31, 32) with library-free directDIA to gauge the bead BG, we detected a staggering 5,779 protein groups (PGs) (*SI Appendix*, Fig. S14*A*). By this reported enrichment protocol, protein-level enrichment of CSC labeled samples only increased the number of detected PGs to 6,198.

To decrease background, we optimized a stringent protocol for protein-level enrichment using phase separation (63), biotin capture in chemically denaturing conditions on surface-blocked streptavidin-coated magnetic particles, and harsh washing with denaturing concentrations of urea and sodium dodecyl sulfate (SDS) (*SI Appendix, Extended Methods*). This optimized enrichment protocol vastly outperformed the old protocol for reducing recovery of nonbiotinylated contaminant proteins, as we detected only 50 PGs in the bead BG and 128 PGs in TFPA BG by the optimized protocol (*SI Appendix*, Fig. S14*B*). However, we note that beads loaded with biotinylated protein may have different adsorptive behavior compared to minimally coated beads, and that we cannot definitively rule out confounding by nonspecific adsorption in biotinylated samples.

### Comparisons at the Proteomics Level for Enrichment of Canonical Cell Surface Proteins.

We used two existing lists of CSPs to define canonical CSPs (cCSPs): the in silico surfaceome SURFY (2,886 proteins) (64) and proteins annotated as PM-associated in Gene Ontology: Cellular Component (GOCCPM) (5,746 proteins) (65, 66). We defined a cCSP to be any protein found in either SURFY or GOCCPM. SURFY is stricter than GOCCPM and GOCCPM covers the majority of SURFY—only 376 SURFY proteins are not in GOCCPM, whereas 3,236 GOCCPM proteins are not in SURFY. Of note, many cell surface localization annotations relied on glycosylated protein recovery in construction; SURFY is a prime example of this, as CSC data were among those used for training (15, 64). Consequently, there is an inherent circularity in benchmarking surfaceomics methods against such reference lists. For an orthogonal noncircular reference list, we used a compiled list, referred to here as CIRFESS TM, from Veneer (67) and CIRFESS (68) of proteins that have annotated transmembrane domains (TMDs) in UniProt (69) or predicted TMDs according to SOSUI (70), TMHMM (71), or Phobius (72). While these domain prediction models are not based on glycan enrichment proteomics, they are expected to miss CSPs that lack a TMD and adhere to the cell surface by protein–protein interactions or lipid anchors.

We first analyzed the datasets based on peptide precursor elution group (EG) quantitation because bottom–up proteomics directly quantifies peptides by elution profile before algorithmically deriving a protein quantity. For robustness, we considered an EG to be detected in a condition only if it was quantified in at least two biological replicates. All tested surfaceomics methods detected numerous cCSP and CIRFESS TM-derived peptides, with EG detections exceeding those of the BG negative controls by two orders of magnitude (*SI Appendix*, Fig. S15*A*). Furthermore, cCSP and CIRFESS TM-derived EGs comprised higher fractions of the EGs in surfaceomics than in unenriched WCL. Using label free quantitation (LFQ) as a proxy for mass, we quantified what fraction of the total EG quantified intensity within each sample was attributable to cCSPs or CIRFESS TM proteins (*SI Appendix*, Fig. S15*B*). All surfaceomics methods showed appreciably higher SURFY fractional intensities compared to WCL, but CSC stood out with exceptionally high fractional SURFY intensity; we were not surprised given the circularity of SURFY’s training set including CSC data. Expanding to all cCSPs diminished the difference between surfaceomics methods and WCL in fractional cCSP intensity; however, surfaceomics methods still outperformed WCL for membrane protein quantitation with nearly doubled CIRFESS TM fraction.

Analyzing algorithmically deduced PG-level quantitative data, we retained only PGs with at least two unique detected peptides for high confidence. For a PG to be considered detected in a condition, it must have been quantitated in at least two biologic replicates. For intercondition comparisons, we employed average replicate intensities with missing not at random (MNAR) imputed values for missing values. We started by counting the number of detected PGs (Fig. 2*B*). All surfaceomics methods detected more than 3,000 PGs. The photo-PLP methods surpassed chemical methods CSC and Sulfo-NHS but detected fewer PGs than enzymatic WGA-HRP. When considering only cCSPs, CSC detected the fewest at 976 PGs while all photo-PLP methods, WGA-HRP, and Sulfo-NHS exceeded 1,000 cCSP PGs. CIRFESS TM PG detection closely reflected cCSP detection (*SI Appendix*, Fig. S16*A*). However, in cCSP fractional intensity (Fig. 2*C*), CSC far exceeded the other surfaceomics methods with >65% of PG-level intensity attributable to cCSPs, whereas photo-PLP methods exhibited similar metrics to WGA-HRP and Sulfo-NHS. We reason that non-CSC methods consistently recovered a higher proportion of non-SURFY proteins than CSC because they are not restricted to biotinylating only glycosylated proteins: In essence, CSC achieves high cCSP specificity at the expense of maximum coverage. We note that our analysis counts the entire signal of a protein group found in a cCSP list as surface-derived even if there are multiple known cellular localizations. If a protein is predominantly cytoplasmic but annotated as a cCSP due to a minor cell surface population, surfaceomics should quantify the cell surface population, whereas WCL should predominantly detect the abundant cytoplasmic fraction and misleadingly inflate the cCSP fraction. Indeed, although WCL showed a similar cCSP intensity fraction to most surfaceomics methods, CIRFESS TM proteins—proteins which should be constrained to a membrane—comprised a smaller intensity fraction of WCL than of any surfaceomics method (*SI Appendix*, Fig. S16*B*).

For another quantitative description of CSP enrichment, we assessed how cCSPs distributed over quantitation intensity across methods (*SI Appendix*, Fig. S17). Under a null hypothesis where cCSPs distribute uniformly over rank, successful CSP biotinylation should bias cCSPs at high quantitation (left-shifted distribution). CSC displayed the strongest left shift. Sulfo-NHS, WGA-HRP, ConA-EY, Chol-EY, and NbALFA-EY also displayed high quantitation preference for cCSPs, but CSC-EY and WGA-EY showed a uniform distribution of CSPs across ranks. Notably, unenriched WCL displayed a low quantitation bias for cCSPs, suggesting that quantifying cCSPs by WCP risks signal suppression, presumably by more abundantly recovered cytoplasmic proteins; this affirms the value of surfaceomics for measuring changes in CSP levels. We confirmed similar results using receiver operating characteristic (ROC) curves over intensity ranks where cCSPs were considered positive hits (*SI Appendix*, Fig. S18). The area under the curve (AUC) for WCL was less than 0.5, the AUC of the uniform null hypothesis, confirming that cCSPs comprise a low abundance population in WCP. In contrast, all surfaceomics methods yielded cCSP AUC greater than 0.5, supporting that surfaceomics detects cCSPs in a greater dynamic range. To determine whether this difference was more than noise, we computed nonparametric bootstrap 95% CI on the differences of AUCs with both the uniform null hypothesis (AUC = 0.5) and empirical WCL AUC value. When the CI entirely exceeded zero, we considered this to support enrichment. All methods except CSC-EY and WGA-EY achieved cCSP enrichment over the uniform null hypothesis; when compared to WCL, all methods including CSC-EY and WGA-EY achieved enrichment.

Next, we compared surfaceomics to WCL by scatter plots (*SI Appendix*, Fig. S19). For this analysis, each sample’s LFQs were normalized to its internal median of detected PG LFQs. The surfaceomics methods promisingly quantified cCSPs–and very starkly for SURFY proteins—above the linear regression line, supporting that CSPs are better quantified and represented in surfaceomics datasets than WCP. To assess statistical significance, we performed pairwise limma (73) contrasts between each surfaceomics method and unenriched WCL (*SI Appendix*, Figs. S20–S22). SURFY proteins biased heavily toward being significantly enriched by surfaceomics methods; this same skew was also present, albeit less stark, for cCSPs and CIRFESS TM proteins. Nonetheless, cCSPs and CIRFESS TM proteins made up a large proportion of the proteins that were significantly enriched by surfaceomics methods (*SI Appendix*, Table S1), with CSC again performing the best. We further sought to confirm CSP enrichment using gene set enrichment analysis (GSEA) (74, 75) comparisons between surfaceomics and WCL, which showed significant enrichment of PM and extracellular space relevant terms by all surfaceomics methods (*SI Appendix*, Fig. S23). From these analyses, we concluded that all the surfaceomics methods reliably enrich CSPs. We performed similar analyses to Bead BG and TFPA BG (*SI Appendix*, Figs. S24–S31 and Tables S2 and S3), but because we downweighed imputed values and the BG samples detected far fewer PGs than surfaceomics samples or WCL, these comparisons were less informative.

### High-Level Comparisons Among Surfaceomics Methods.

We broadly compared surfaceome enrichment schemes. First, we performed principal component analysis (PCA) and Pearson correlation analysis at both the EG and PG levels (*SI Appendix*, Fig. S32). For these analyses, PGs that were not detected in at least two replicates were still included to assure identical dimensionality across samples, but missing values were imputed by the MNAR algorithm. All surfaceomics methods clustered with each other, while unenriched WCL and negative controls were excluded from this cluster—this suggested that the compositional difference between WCL and surfaceomics is not driven by the bead enrichment support. Within the surfaceomics subcluster, glycan-restricted CSC separated away from all other surfaceomics methods.

We then evaluated the overlap of detected PGs (Fig. 2*D*). All eight surfaceomics methods shared 2,992 detected PGs, of which 797 are cCSPs. 1,246 cCSPs were among the 5,282 total PGs detected by at least half of the surfaceomics methods. Only 130 PGs in total were seen by bead BG and TFPA BG, suggesting most shared PGs were not merely adsorbed to the bead surface. As the surfaceomics methods are mechanistically orthogonal, consistent identification across multiple surfaceomics methods but not by the negative controls boosts confidence that these proteins are truly at the cell surface. However, as aforementioned, we cannot definitively rule out adsorption of nonbiotinylated proteins facilitated by biotinylated proteins despite taking steps to minimize such adsorption.

**Fig. 3.**
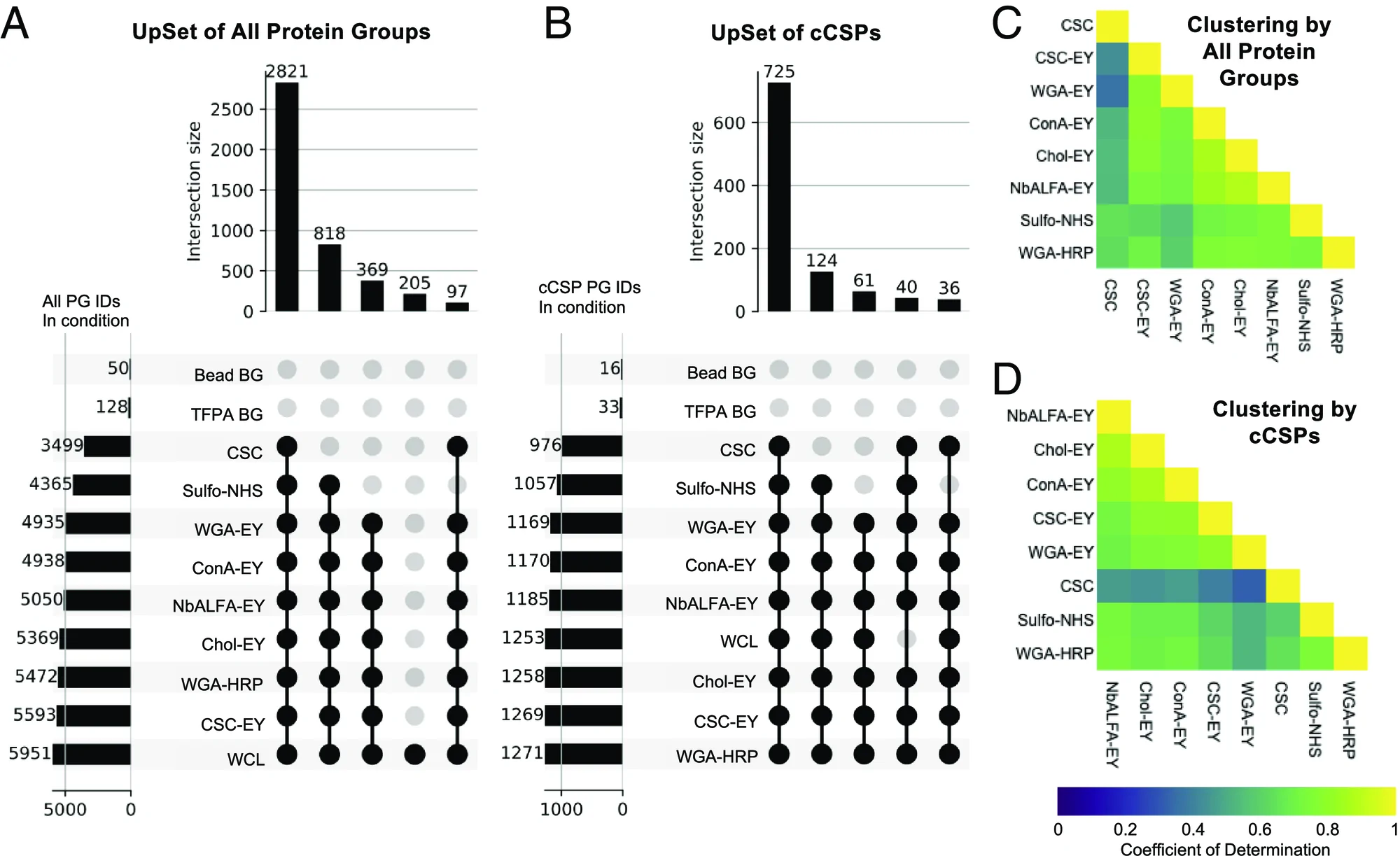
Diffusive surfaceomics methods are broadly similar but differ most from glycan-restricted CSC. (*A*) PG-level UpSet plot of set intersections. Top five sets by size are shown. (*B*) UpSet plot restricted to only cCSPs. Top five sets by size are shown. (*C*) Heatmap of pairwise coefficients of determination across surfaceomics methods. (*D*) Heatmap of pairwise coefficients of determination restricted to only cCSPs.

UpSet plot analysis (76) based on PG detections (Fig. 3 *A* and *B*) strikingly revealed the largest set was 2,821 PGs detected by all surfaceomics conditions as well as WCL but not the BG negative controls. Of these, 725 were cCSPs. An additional 43 PGs were detected in all surfaceomics methods but not WCL or BG samples; 40 of these are cCSPs. The seven surfaceomics methods that are not glycan-restricted detected 818 PGs (124 cCSPs) that CSC and the BG controls missed. Affirming the mechanistic validity of our data, only 2.7% of PGs that were missed by CSC but seen by all seven other surfaceomics methods are annotated as N-glycosylated in UniProtKB (69) (*SI Appendix*, Fig. S33); in contrast, 12.0% of PGs identified by all eight surfaceomics methods and 13.7% of PGs identified by CSC had N-glycosylation annotations. These percentages were far lower than the N-glycosylation annotations of the SURFY and GOCCPM databases (SURFY 86.6%; GOCCPM 49.7%), underscoring that surfaceomics is an enrichment but not a purification technique. While protein-level enrichment may contribute persistent background, we also found it curious that there was high concordance across surfaceomics methods in which nonglycosylated proteins they recovered. We performed pairwise Venn diagram comparisons of all surfaceomics methods and WCL to CSC for PGs detected (*SI Appendix*, Fig. S34). This confirmed that the non-CSC surfaceomics methods overall detected many more PGs missed by CSC—on the order of thousands. Nonetheless, CSC preferentially detected some PGs—many of which were SURFY proteins—over other surfaceomics methods.

We next considered pairwise comparisons of the surfaceomics methods (*SI Appendix*, Figs. S35–S37) and evaluated their pairwise coefficients of determination (Fig. 3 *C* and *D*). For these and subsequent analyses, surfaceomics LFQs were normalized to their internal median LFQ of endogenously biotinylated carboxylases. This endogenous biotinylation normalization accounts for the differing degrees of artificial biotinylation across the surfaceomics methods, which was apparent in the mass spectrometry data (*SI Appendix*, Figs. S38–S42), and operates under the assumption that endogenous biotinylation is consistently present and recovered equally across samples. There were strong correlations between surfaceomics datasets overall. The photo-PLP methods showed high similarity to each other as well as to WGA-HRP and Sulfo-NHS where labeling occurs on proteins themselves, while CSC that only labels glycans differed the most. This analysis highlighted that the methods have complementary benefits since each surfaceomics method could detect some cCSPs missed by another.

We wondered whether the molecular weight biases seen by Western blot would appear in proteomics. Using molecular weights deposited in UniProtKB (69), we analyzed the distribution of detected PGs in each sample (*SI Appendix*, Fig. S43). Interestingly, the mass spectrometry did not recapitulate the trends from Western blot despite being from the same samples. One reason for this discrepancy could be posttranslational processing of proteins: Many small extracellular proteins are generated from proteolysis of much larger precursors (77), and extracellular proteolysis is rampant on cancer cell surfaces (78). Such proteolyzed proteins would appear large by this UniProt MW analysis but small by Western blot. Another potential explanation—and a caveat that must be applied to all biotin-enrichment proteomics experiments without residue level evidence of biotinylation—is adsorbed nonbiotinylated proteins: High sensitivity proteomics may detect them while they would not appear by Western blot.

### Comparisons of Proteomics Based on Spatial Location and Molecular Target of the EY Catalyst.

**Fig. 4.**
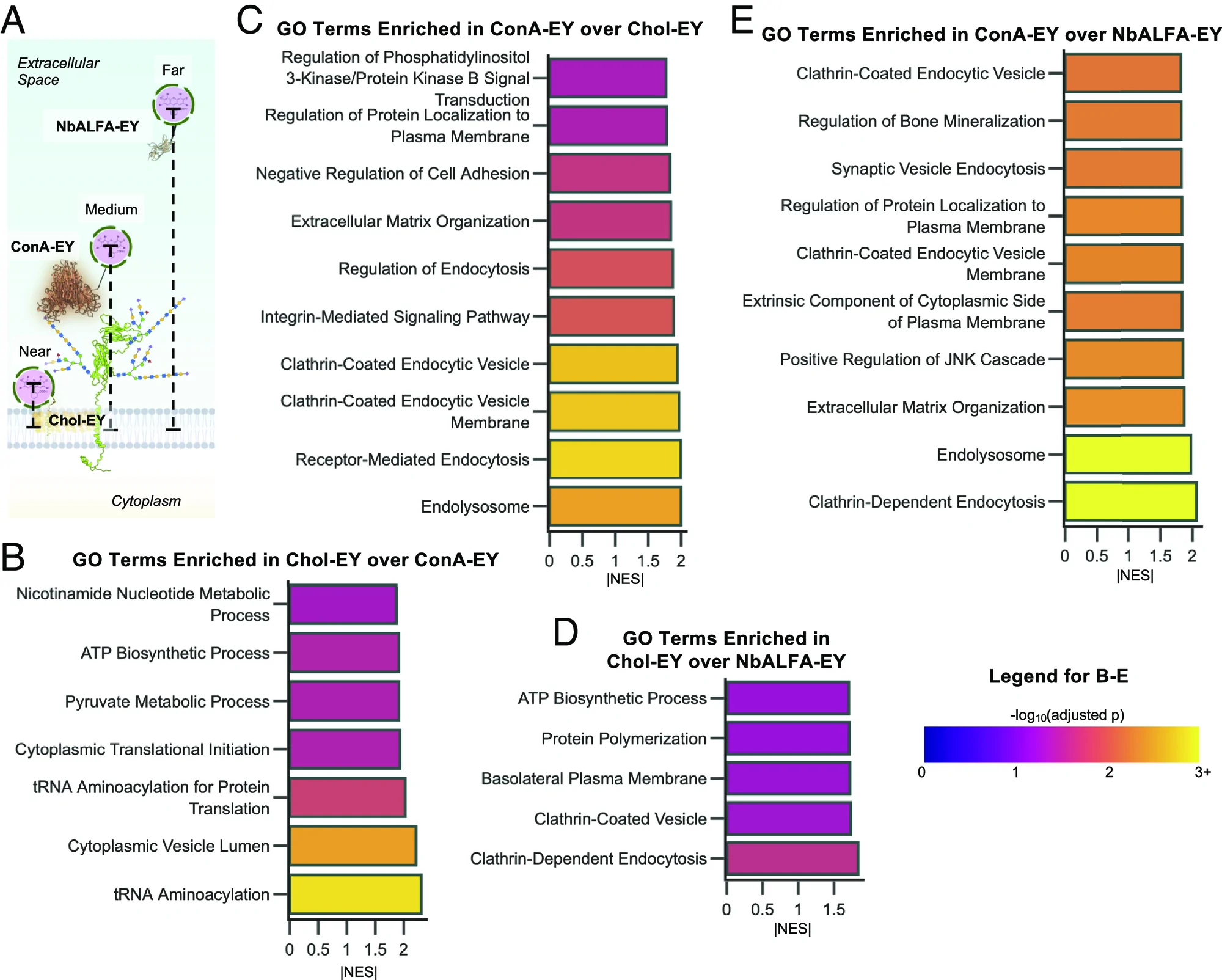
Differential localization of photocatalyst captures differing features of the cell surface. (*A*) Chol-EY, ConA-EY, and NbALFA-EY have different spacing from the PM. Figure created with Biorender.com. (*B*) GSEA shows greater cytoplasm-associated annotations in Chol-EY compared to ConA-EY. All significant terms (FDR < 0.05 and normalized enrichment score (NES) greater than 1.5) are shown. (*C*) ConA-EY enriches plasma membrane-related features compared to Chol-EY. The top 10 significant terms ranked by NES are shown. (*D*) Chol-EY significantly enriches plasma membrane proteins over NbALFA-EY. All significant terms are shown. (*E*) ConA-EY enriches plasma membrane proteins, especially endocytosis-relevant proteins, relative to NbALFA-EY. The top 10 significant terms, ranked by NES, are shown.

To analyze specific differences between surfaceomics methods, we performed differential analysis and bioinformatic enrichment analysis using endogenous biotin-normalized LFQs. We first asked whether the spatial distance between EY and the PM influences which CSPs are enriched by comparing PM-incorporated Chol-EY, ectodomain glycosylation-tethered ConA-EY, and labeling from solution by NbALFA-EY (Fig. 4*A*). Comparing tethered methods ConA-EY and Chol-EY by GSEA, Chol-EY enriched proteins associated with cytoplasmic and mitochondrial pathways (Fig. 4*B*), whereas ConA-EY enriched vesicular transport and extracellular matrix (ECM) related proteins (Fig. 4*C*). The enrichment of intracellular annotations by Chol-EY likely is, at least partially, attributable to aforementioned internalization of Chol-EY. ECM enrichment by ConA-EY may result from the spatial separation between the PM and bulky CSP ectodomains that interact with ECM. Enrichment of endocytic proteins by ConA-EY may result from the tetravalency of ConA that enables CSP clustering; clustering may promote association with endocytic machinery and clathrin-coated pit (CCP) formation even at 4 °C (79, 80). Moreover, multiple CSPs that ConA-EY could bind such as LDLR and TFRC reside natively in CCPs (81, 82). However, as ConA-EY binding, irradiation, and washing are performed at 4 °C, we do not expect internalization by active endocytosis due to inhibition of budding and scission (83–85). This is consistent with the confocal microscopy that showed ConA-EY biotinylation signal remained at the cell surface without noticeable intracellular contributions.

Volcano plot analysis (*SI Appendix*, Fig. S44*A*) affirmed that Chol-EY enriched proteins known to reside in cholesterol-rich microdomains compared to ConA-EY, consistent with Chol-EY localizing to these microdomains (48), whereas ConA-EY enriched CCP-resident TFRC (81). Endocytosis-relevant AP2 subunits were also enriched by ConA-EY, even though AP2 associates with the PM from the cytoplasmic side. We believe that PM-associated cytosolic proteins like AP2 are detected despite ConA-EY not internalizing, as there is literature precedence of extracellular EY photo-PLP detecting proximal intracellular proteins at 4 °C (31, 33) (*Discussion*). Both ConA-EY and Chol-EY detected proteins that were not annotated as cCSPs but have PM-relevant functions; many of these may also be PM-proximal intracellular proteins.

The difference between tethered and nontethered surfaceomics is an unresolved topic in the PLP field (12, 13), so we compared ConA-EY and Chol-EY pairwise to nontethered NbALFA-EY. Both ConA-EY and Chol-EY enriched PM features over NbALFA-EY (Fig. 4 *D* and *E*). This is largely due to greater efficiency in labeling CSPs, as evident from asymmetry in volcano plots (*SI Appendix*, Fig. S44 *B* and *C*). Interestingly, among the few proteins NbALFA-EY enriched were the 1,238 amino acid JAG2 and the 4,391 amino acid HSPG2, some of the most protrusive CSPs known (*SI Appendix*, Figs. S45 and S46).

Next, we compared ConA-EY to the other glycan-tethered diffusive surfaceomics methods CSC-EY, WGA-EY, and WGA-HRP (*SI Appendix*, Fig. S47). Consistent with expectations, ConA-EY enriched PM-relevant annotations over both CSC-EY and WGA-EY, whereas CSC-EY and WGA-EY enrichment favored spliceosome and RNA binding proteins. While the greater biotinylation efficiency of enzymatic HRP over EY contributed to only one GO term being enriched by ConA-EY over WGA-HRP, surprisingly only one of the WGA-HRP enriched terms was PM-relevant—the rest pertained to the nucleus and RNA. The repeated enrichment of spliceosome, nuclear, and RNA-related annotations was unexpected but may result from molecular localization target (*Discussion*).

Whereas endogenous biotin normalization can capture differing degrees of biotinylation, we also considered whether molecular biases of each surfaceomics method would be more apparent by GSEA on median normalized LFQs (*SI Appendix*, Figs. S48–S53). We observed similar trends as with endogenous biotin normalization.

### Comparison Among Methods for Identification of Noncanonical Cell Surface Proteins.

Recent studies have identified noncanonical CSPs (nCSPs)—proteins that lack PM-directed signal sequences, TMDs, or extracellular anchoring lipids yet localize to the cell surface—in cancer (23, 34). We reasoned that as our photo-PLP surfaceomics methods generate reactive intermediates that diffuse and insert nonspecifically into proximal proteins, they may be particularly suitable for systematically detecting nCSPs. Our surfaceomics methods consistently detected many recently described nCSPs including Inside-Out (IO) proteins (23) and intracellular myristoylated proteins flipped to the extracellular PM via autophagolysosomal exocytosis (ALE) (34) (*SI Appendix*, Fig. S54). These known nCSPs distributed across a wide intensity range in WCL (*SI Appendix*, Fig. S55), suggesting that they may be on the cell surface and not merely high abundance intracellular contamination.

**Fig. 5.**
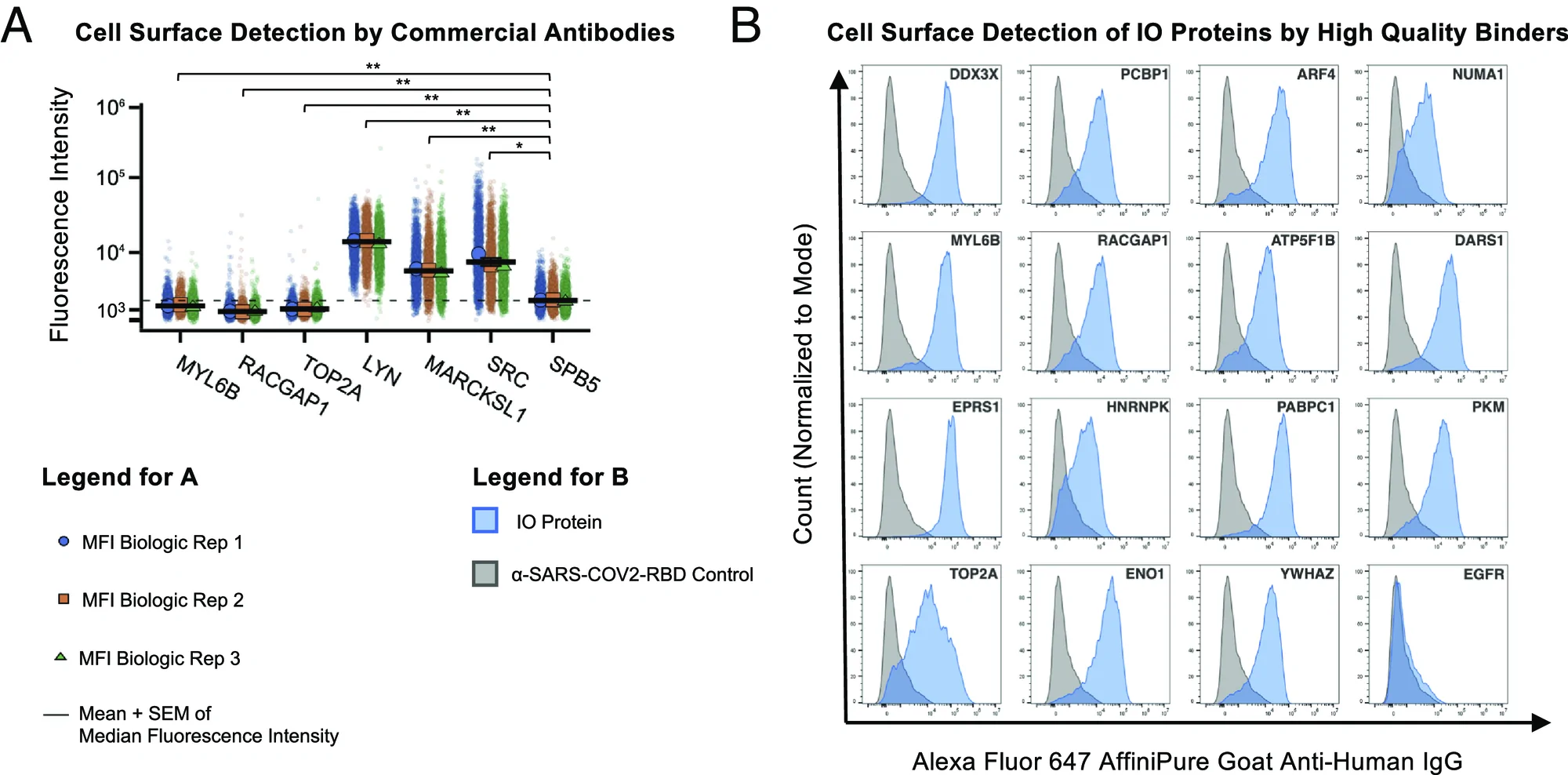
Known noncanonical cell surface proteins detected by surfaceomics are detectable by antibodies. (*A*) Commercial antibody staining for known nCSPs. Significance reflects a two-tailed Welch’s *t* test (86) compared to SPB5. **P < 0.01. *P < 0.05. ns: *P* ≥ 0.05. The *y* axis is Logicle (87) transformed. (*B*) Representative histograms for cell surface binding of antibodies against 15 IO proteins (blue). An isotype control from the same library targeting SARS-COV2-RBD (gray) is provided as reference and anti-EGFR is included as a negative control.

To confirm whether these nCSPs were detected because they are truly present at the cell surface, we stained for six nCSPs [three ALE proteins (LYN, MARCKSL1, and SRC) and three IO proteins (MYL6B, RACGAP1, and TOP2A)] using commercial antibodies on nonpermeabilized cells. By flow cytometry, we detected appreciable levels of extracellular LYN, MARCKSL1, and SRC over negative control SPB5, which is not expressed in K562 (3) or found in our proteomics data, but could not detect the tested IO proteins (Fig. 5*A* and *SI Appendix*, Fig. S56). Confocal microscopy recapitulated these findings (*SI Appendix*, Fig. S57). While the ALE proteins are known to associate with the cytoplasmic face of the plasma membrane, these data indicate they are also on the extracellular face of the cell surface, consistent with a recent report (34). We wondered whether the IO proteins are truly not present on the cell surface or if the commercial antibodies used were insufficient for detecting the native extracellular fold of these IO proteins. Using validated high-quality binders (23), we confirmed cell surface presence of 15 published IO proteins detected in our surfaceomics data that vastly exceeded negative control EGFR, which is not expressed in K562 (3) or detected in our surfaceomics data (Fig. 5*B*). This finding suggests that nCSPs may adopt a different fold or have masked epitopes in the extracellular environment; alternatively, it may simply reflect differences in antibody quality for binding the native protein. The difficulty this presents for antibody-based validation underscores the value of mass spectrometry-based approaches like photo-PLP surfaceomics.

Considering our success in validating previously discovered nCSPs, we wondered whether surfaceomics could guide prioritization of nCSP candidates. To this end, we evaluated PGs by their relative enrichment in each surfaceomics method over WCL by three metrics: Limma contrasts (73) with median normalization, pairwise rank permutation (88), and robust z-score (z*) across detected PGs. We considered PGs with |log_2_(fold change)| ≥ 1 and adjusted *P* < 0.05 to be enriched by limma, false discovery rate (FDR) < 0.05 to be enriched by rank permutation, and z* > 2 to be enriched by robust z-score. We note that the z* > 2 threshold identifies surfaceomics-preferred outliers but does not have a statistical testing metric. Filtering detected PGs for these enrichment thresholds (*SI Appendix*, Fig. S58) yielded more stringent enriched PG lists and increased cCSP representation, as expected of robust surfaceomics methods. Although the robust z-score lacked a statistical significance metric, it was stricter than either limma or rank permutation. PGs repeatedly enriched by different surfaceomics methods biased for cCSPs, as expected, whereas PGs not in a cCSP list were more frequently detected by a small number of surfaceomics methods. We applied this analysis to the literature reported IO and ALE nCSPs (*SI Appendix*, Fig. S59). Interestingly, we found that many IO and ALE proteins were not enriched—many of these were depleted by surfaceomics compared to WCL, presumably because their canonical intracellular pools are dominant. However, we noted that those that did pass the enrichment threshold showed notable cell surface signal in our flow cytometry validations.

**Fig. 6.**
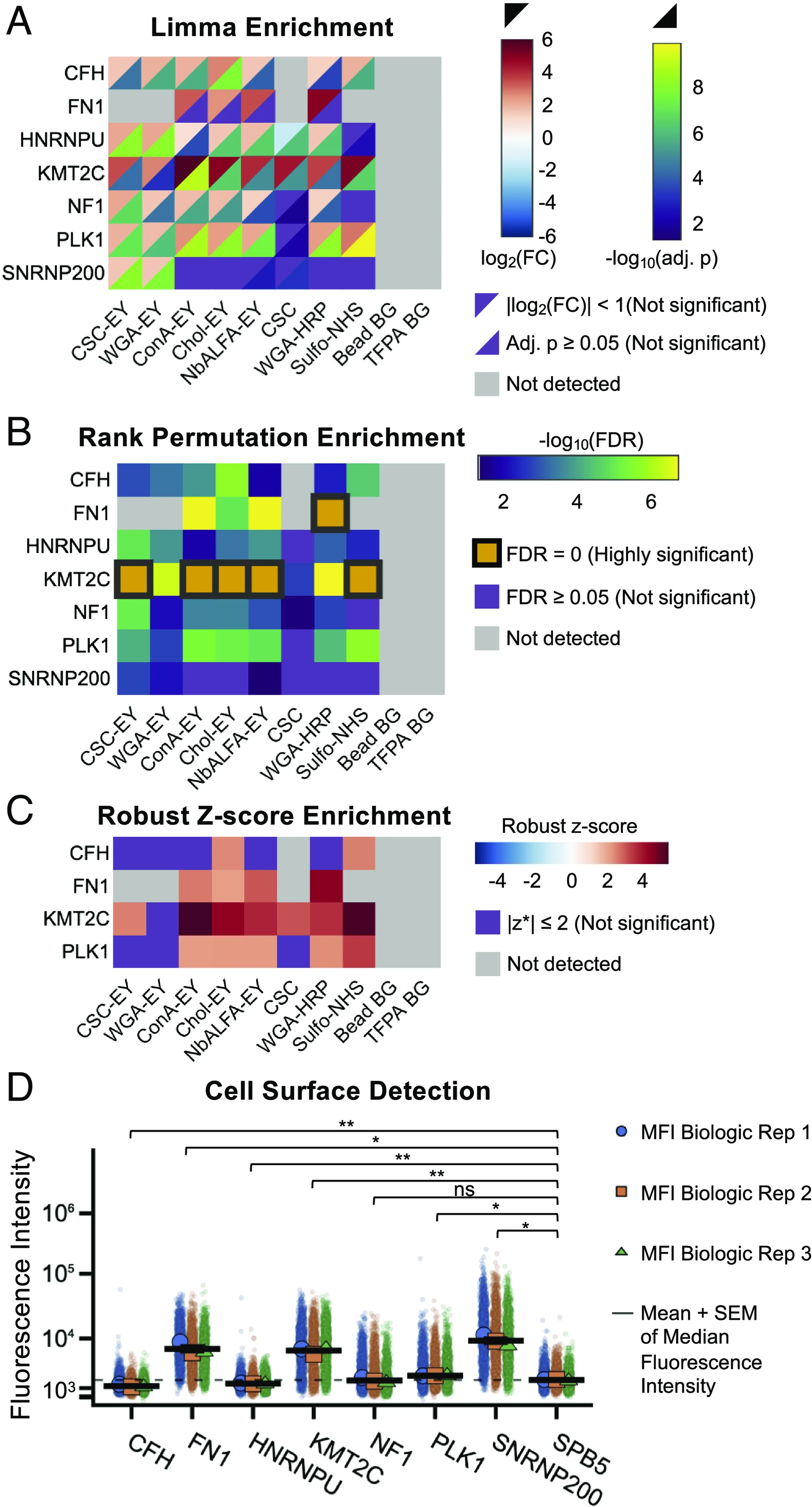
Photo-PLP surfaceomics enables nCSP discovery. Enrichment signatures of seven selected CSP candidates relative to whole cell lysate by median normalized intensities using (*A*) limma, (*B*) rank permutation, and (*C*) robust z-score. HNRNPU, NF1, and SNRNP200 are not shown in (*C*) because they did not meet the robust z-score significance threshold of z* > 2 in any condition. (*D*) FN1, KMT2C, and SNRNP200 show cell surface staining on nonpermeabilized K562. Significance reflects a two-tailed Welch’s *t* test (86) compared to SPB5. **P < 0.01. *P < 0.05. ns: *P* ≥ 0.05. The *y* axis is Logicle (87) transformed.

We selected seven candidates (CFH, FN1, HNRNPU, KMT2C, NF1, PLK1, SNRNP200) with distinct enrichment signatures for validation (Fig. 6 *A*–*C*): CFH, KMT2C, NF1, and PLK1 have not been reported at the cell surface and are nCSP candidates proposed from our surfaceomics datasets; HNRNPU and SNRNP200 are recently reported cancer-specific nCSPs (35, 89); and FN1 is a secreted cCSP that is not in SURFY. We selected KMT2C as it was highly enriched by multiple surfaceomics methods, reaching FDR = 0 significance in four of the five photo-PLP methods. CFH and FN1 were selected as they were not detected by CSC but were enriched by photo-PLP; in the case of FN1, the three photo-PLP methods that had the highest cCSP enrichment (ConA-EY, Chol-EY, and NbALFA-EY) all showed enrichment by rank permutation and robust z-score but not by limma. NF1 was chosen as it was best enriched by CSC-EY and weakly enriched by other surfaceomics methods except Sulfo-NHS. PLK1 represented a protein that was enriched by ConA-EY, Chol-EY, NbALFA-EY, WGA-HRP, and Sulfo-NHS but not CSC by all metrics. Both literature reported nCSPs in this candidate set (HNRNPU and SNRNP200) were enriched by a photo-PLP method but not CSC by rank permutation; for SNRNP200, none of the literature reported surfaceomics methods displayed significant enrichment.

Using the same commercial antibody validation flow cytometry workflow described above, we detected cell surface presence of FN1, KMT2C, and SNRNP200, but not CFH, NF1, or HNRNPU (Fig. 6*D* and *SI Appendix*, Fig. S60). PLK1 showed mildly increased signal over the SPB5 negative control, but due to the small effect size we hesitate to declare it a positive validation. While not all seven candidates validated, we find the validation of those that did encouraging considering the difficulty of detecting nCSPs by antibodies (23). This is underscored by the inability to detect cell surface HNRNPU by flow cytometry, even though HNRNPU was previously shown to be on the cell surface of K562 (35). The domains used to generate antibodies against cytoplasmic proteins may be hidden, perhaps occluded by noncanonical PTMs like unexpected compartment-specific glycosylation (35), or adopt a different fold when such protein is at the cell surface as a nCSP, which would result in a false negative. Moreover, validation of antibodies often occurs in the context of denaturation as in Western blot or fixation as in intracellular flow cytometry, which would lead to differences from the native nCSP fold.

Surface validation of FN1 is noteworthy as this protein is known to be secreted into the extracellular space, providing evidence that secreted proteins which rebind to the cell surface (90, 91) are detectable by diffusive surfaceomics. Moreover, despite being canonically glycosylated, FN1 was not detected by CSC or Sulfo-NHS. A caveat is that FN1 is abundant in fetal bovine serum (FBS) and shares 90% homology to human FN1 (92); the anti-FN1 antibody used is likely cross-reactive to bovine FN1, and analysis of the mass spectrometry did not show unique human FN1 peptides (*SI Appendix*, Fig. S61). Thus, it is possible that the FN1 signal results from specific binding or even nonspecific adsorption of bovine FN1 to the cell surface. However, even in this case, there may be biologic relevance to cell surface detection of FN1: The concentration of FN1 in human blood is approximately 150 mg/L (93), whereas the concentration of bovine FN1 in standard 10% FBS cell culture medium is only approximately 3 mg/L (94). If extracellular FN1 can associate with the surface of K562 cells in culture—whether secreted by the cells or derived from FBS—it is plausible that plasma FN1 similarly associates with the cell surface in vivo. This highlights the potential utility of diffusive surfaceomics for detecting secreted extracellular proteins that rebind to the cell surface.

Aside from FN1, the other two proteins that were detected at the cell surface by flow cytometry lack features of cCSPs like a TMD. The lysine methyltransferase KMT2C was detected in all eight surfaceomics methods and robustly enriched by photo-PLP methods. The validation of cell surface KMT2C by flow cytometry is particularly salient given that noncanonical PTMs on CSPs including methylation are increasingly appreciated and may provide therapeutic targets (95). Successful validation of cell surface SNRNP200 is also informative: SNRNP200 was not enriched according to robust z-score, but it passed enrichment thresholds for limma and rank permutation by some photo-PLP methods—and not in any of the chemical or enzymatic surfaceomics methods. This suggests that the more lenient enrichment metrics still yield true positives. In totality, we believe that this evidence supports the utility of photo-PLP surfaceomics for prioritizing nCSP candidates.

## Discussion

Here we developed and compared five unique photo-PLP methods that significantly expand our ability to study the surfaceome. We explored ways to tether EY to the cell surface or restrict it to extracellular space to diffusively biotinylate CSPs. All constructs are readily generatable and can be simply added to native cells, making them broadly accessible to investigators and promising for primary biological samples.

Our experiments demonstrate the utility of surfaceomics in both providing localization-specific information as well as robust quantitation of CSPs, as all surfaceomics methods improve the quantitative representation of cCSPs relative to WCP. One confounder in this comparison is that to accurately represent WCP, WCL samples were prepared per PreOmics manufacturer directions. The proprietary PreOmics LYSE buffer, which contains sodium deoxycholate (SDC) and is derived from a 1% SDC buffer (96), differs from the 1× RIPA lysis buffer (50 mM Tris-HCl, pH 7.4, 150 mM NaCl, 0.25% deoxycholic acid, 1% NP-40, 1 mM EDTA) used for surfaceomics and BG samples. Lysis buffer can impact membrane protein recovery (97–99), but 1% SDC buffers extract membrane proteins well (99) and exceed the deoxycholic acid concentration in 1× RIPA, making this an unlikely cause for the lower cCSP recovery by WCL.

Photo-PLP offers numerous advantages for surfaceomics that previously have not been fully leveraged. Established methods such as CSC for labeling glycoproteins and Sulfo-NHS for labeling amines have chemical restrictions. In contrast, EY can activate diffusive biotin probes like TFPA that insert into C-C, C-H, and C-N bonds present in all biomolecules (32, 57). Furthermore, EY-compatible photoprobes cover multiple labeling ranges (31, 33, 100); while we used only TFPA in this comparative study, other photoprobes may be used with the described photo-PLP methods. A key advantage of diffusion-based surfaceomics over the glycosylation-restricted gold standard CSC is increased coverage, especially of nonglycosylated CSPs. Moreover, unlike enzymatic peroxidase-based techniques that are performed at 37 °C in the presence of millimolar concentrations of toxic hydrogen peroxide, photo-PLP methods are temporally controlled by visible light and viable at 4 °C where membrane trafficking is halted. Even though peroxidase-based techniques require less reaction time than photo-PLP (2 min for peroxidases (11, 12) compared to 5 min for photo-PLP), reduced diffusion kinetics at 4 °C help photo-PLP achieve a high resolution, high “shutter speed” molecular photograph of the cell surface.

Among photo-PLP methods, differences in enriched proteins arise. Molecular localization target may contribute to MW biases. CSC-EY, WGA-EY, and WGA-HRP biotinylated proteins with MW below 20 kDa and enriched DNA binding and ribosomal proteins—which frequently are observed in this MW range (101). A notable similarity among these methods is that they can attach to canonically intracellular molecules such as O-GlcNAc for WGA-EY and WGA-HRP or O-glycosylation, ADP-ribose, and RNA for CSC-EY. Indeed, canonically intracellular proteins enriched by these specific methods include spliceosome proteins and transcription factors, which can be O-GlcNAc modified (102, 103), ADP-ribosylated (104), or RNA binding (105). Thus, targeting EY to canonically intracellular PTMs may underlie the strong detection of these protein classes. We cannot exclude intracellular biotinylation from decreased PM integrity or dead cells; however, RNA binding proteins were recently found to cluster around cell surface RNA on cancer cells including K562 (35, 106).

Much remains to be studied about the functions of the intracellular proteins that are aberrantly presented on the cell surface in cancer as nCSPs (23, 34, 35, 89). Diffusion-based photo-PLP surfaceomics is a promising approach to identify new nCSP candidates. Validation of any new nCSP is strictly necessary: Mass spectrometry alone cannot distinguish true nCSPs from abundant intracellular contaminant. As a start, we showed flow cytometry validation of nCSPs that we detected by photo-PLP. This can be challenging in the absence of high-quality binders, and future studies may benefit from validation by other cellular and biochemical methods such as bispecific T cell engager (BiTE) activation assays and gene knockdown. Beyond validating cell surface presence, much remains to be learned about how these nCSPs reach the extracellular space; ALE only explains the extracellular presence of lipidated nCSPs (34). While it is known how some nCSPs tether to the cell surface (23, 35, 89), it is unclear whether they are trafficked to the cell surface in a regulated manner or released into extracellular space from lysed cells. Regardless of their mechanistic origin, nCSPs are an underappreciated facet of cell biology and have therapeutic potential as an exploitable feature of dysregulated diseases like cancer.

While we believe that many of the proteins we detected are true nCSPs, in the absence of validation we cannot rule out the possibility that some are cytoplasmic proteins. There is precedence for detecting proximal intracellular proteins by extracellular photo-PLP (33). However, cytoplasmic proteins biotinylated by photo-PLP surfaceomics should be limited to those proximal to the PM due to quenching of activated aryl nitrenes on PM lipids. An alternate explanation for the recovery of intracellular proteins is singlet oxygen mediated crosslinking. Activated EY can generate membrane-permeant singlet oxygen (50, 107) that may oxidize intracellular proteins (25, 48) to covalently crosslink them to biotinylated CSPs. This phenomenon may underlie the mild cell surface adsorption of NbALFA-EY following irradiation. Progression in the photo-PLP field to releasable photoprobes (108, 109) will provide residue level evidence of probe insertion to help distinguish this.

Targeted photo-PLP has emerged as an excellent approach to study protein neighborhoods; our photo-PLP surfaceomics methods have utility here. Targeted photo-PLP studies often use a primary antibody against the target and a secondary antibody conjugated with the photocatalyst. As a reference control for global protein abundance, investigators often employ the secondary conjugate in the absence of targeted primary (29) but wash it away before irradiation. Our group previously showed that such an untethered, washed-away PLP control underlabels the cell (32). When there is trace biotinylation, the resulting sparse data do not always align with statistical assumptions used by label-free quantitation algorithms (110) and differential analysis (73). All the photo-PLP surfaceomics techniques described here are viable reference controls for revealing CSPs enriched in a neighborhood. We particularly recommend ConA-EY, but the other methods have their own strengths and best use cases. We tabulate the advantages and disadvantages of the tested surfaceomics methods in Table 1.

**Table 1.** Evaluation of surfaceomics methods

Method	Type	Kinetic Control/Temporal Resolution	Localization	Biotin Insertion	Biotinylation Radius	Advantages	Disadvantages
CSC-EY	Photocatalytic	High (light)	syn-vicinal diol (sugars including sialic acid, D-mannose, and D-galactose of glycans)	All molecules	Modulatable (100–3,000 Å)	Compatible with 4 °C; not PTM or residue biased; binds cell surface vicinal diols (glycosylation, ribosylation)	Intracellular trapping of EY yields intracellular contamination; may bind intracellular PTMs in membrane-permeabilized cells
WGA-EY	Photocatalytic	High (light)	Sialic acid, N-acetyl-D-glucosamine (found in glycosylation)	All molecules	Modulatable (100–3,000 Å)	Compatible with 4 °C; not PTM or residue biased; cell surface O-GlcNac binding	Agglutination; cellular integrity decrease; signaling activation (inhibited by cold); may bind intracellular PTMs in membrane-permeabilized cells
ConA-EY	Photocatalytic	High (light)	alpha-D-mannose, alpha-D-glucose in glycans	All molecules	Modulatable (100–3,000 Å)	Compatible with 4 °C; not PTM or residue biased	Signaling activation and agglutination risk (inhibited by cold)
Cholesterol-EY	Photocatalytic	High (light)	Lipid bilayer (especially cholesterol-rich microdomains)	All molecules	Modulatable (100–3,000 Å)	Labeling compatible with 4 °C; not PTM or residue biased; strong detection of cholesterol rich microdomain proteins	Lower detection of proteins outside cholesterol rich microdomains; incorporation at biologic temperature contributes intracellular signal
NbALFA-EY	Photocatalytic	High (light)	None (soluble)	All molecules	Modulatable (100–3,000 Å)	Compatible with 4 °C; not PTM or residue biased; detects CSPs with large ectodomains	Lower efficiency due to distance from PM
CSC	Chemical	Low	syn-vicinal diol (sugars including sialic acid, D-mannose, and D-galactose of glycans)	Oxidized sugar	N/A	Highly specific for canonical CSPs; very well tolerated; compatible with 4 °C	Only captures glycoproteins; incomplete view of nCSPs present at the cell surface
WGA-HRP	Enzymatic	Medium (radical quenching)	Sialic acid, N-acetyl-D-glucosamine (found in glycosylation)	Tyrosine	Long (3,000 Å)	Highly efficient; not PTM biased	Requires hydrogen peroxide and generates reactive oxygen species; may induce endocytosis; agglutination; requires biologic temperature; limited to tyrosine; severe viability detriment
sulfo-NHS	Chemical	Low	All extracellularly exposed proteins	Lysine, N terminus	N/A	Very well tolerated; compatible with 4 °C; not PTM biased	Intracellular protein contamination with decreased PM integrity; instability of NHS esters in aqueous solution; requires more basic pH 8.0; only biotinylates lysine and unmodified N termini

In conclusion, we have performed eight different surfaceomics methods, including five photo-PLP methods, in the K562 cell line and compared their performance. Photo-PLP offers a high sensitivity picture of the surfaceome that fills blind spots of established surfaceomics tools, especially for nCSPs. Although the generality of our findings may be limited because only one suspension cell line was used, we believe these methods are widely applicable across different cell lines, including adherent cells, and hope that they will prove useful in future studies of the cell surface proteome, further the discovery and characterization of nCSPs, and advance photo-PLP neighborhood studies for discovering new drug targets.

## Materials and Methods

Detailed materials and methods are described in the *SI Appendix*.

### Cell Culture.

K562 cells were obtained from ATCC and cultured under standard conditions.

### Cell Surface Labeling by Photo-PLP.

Harvested cells were kept at 4 °C or on ice for all subsequent steps. After washing with DPBS, cells were incubated with 10 μΜ photo-PLP EY conjugate. For CSC-EY, cells were reacted first with 500 μM NaIO_4_ with 1:1,000 aniline at pH 6.5 followed by 50 μM aminooxy-EY at pH 8.0. Cells were washed with DPBS, incubated with TFPA-biotin, irradiated by 525 nm LED for 5 min, washed with DPBS, then flash frozen in liquid nitrogen. NbALFA-EY was not washed away before TFPA-biotin incubation and irradiation.

### Proteomics.

Cells were lysed in 1× RIPA with 1 mM EDTA and protease inhibitor, then precipitated with methanol, chloroform, and water per Wessel-Flügge (63). Precipitated protein was resuspended in 4 M urea 0.5% SDS 50 mM Tris pH 8.0 and bound to SeraMag SpeedBeads Streptavidin Blocked Magnetic Beads. Beads were washed with a sequence of four wash buffers (strongest wash 8 M urea 1% SDS 50 mM Tris pH 8.0) then processed using the PreOmics iST 96× kit.

## Supplementary Material

Appendix 01 (PDF)

## Data Availability

Code is available at https://gitfront.io/r/wwho99/U4MVEQvX7zQi/Comparative-Analysis-Canonical-and-Noncanonical-Surfaceomes/ (111). All raw and searched mass spectrometry data have been deposited to the ProteomeXchange Consortium via the MassIVE partner repository with the dataset identifier PXD080445 (112). All other data are included in the article and/or *SI Appendix*.
