# Supplementary material for: Comparative analysis of photocatalytic, enzymatic, and chemical approaches to identify canonical and noncanonical surfaceomes
Source: Proc Natl Acad Sci U S A. 2026 Oct 21;123(43):e2623700123. doi: 10.1073/pnas.2623700123 (PMC13715755; doi:10.1073/pnas.2623700123)
Supplement: Supplementary file 1 [file pnas.2623700123.sapp.pdf]

PNAS

Supporting Information for

Comparative Analysis of Photocatalytic, Enzymatic and Chemical Approaches to Identify Canonical and Noncanonical Surfaceomes

William W. Ho[1], Yifei Chen[1], Tomasz Slezak[2], Oleksandr Zginnyk[1], Trenton M. Peters-Clarke[1], Kaitlin Schaefer[3], Anthony A. Kossiakoff[2,4], Kevin K. Leung[1], James A. Wells[1,5]*

[1]Department of Pharmaceutical Chemistry, University of California, San Francisco, San Francisco, CA 94158, USA
[2]Department of Biochemistry and Molecular Biology, The University of Chicago, Chicago, IL 60637
[3]Department of Microbial Pathogenesis, Yale University School of Medicine, New Haven, CT 06519, USA
[4]Institute for Biophysical Dynamics, The University of Chicago, Chicago, IL 60637
[5]Department of Cellular and Molecular Pharmacology, University of California, San Francisco, San Francisco, CA 94158, USA

*Corresponding author: James A. Wells
Email: jim.wells@ucsf.edu

This PDF file includes:

Supporting text
Figures S1 to S61
Tables S1 to S3
SI References

Supporting Information Text

Extended Methods

Cell Culture: K562 cells were purchased from ATCC and cultured in RPMI-1640 (Cytiva SH30027.01) with 10% FBS (GeminiBio 100-106) and 1% Penicillin-Streptomycin (Thermo 15140122) under standard conditions. Cells were confirmed negative for mycoplasma by polymerase chain reaction (1).

Preparation of Eosin Y conjugates

Synthesis of conjugation starting materials: EY-PEG_4-DBCO (EY-DBCO) was synthesized and purified as previously described (2). Oxaziridine-azide was synthesized as previously described (3).

Synthesis of aminooxy-PEG-EY: Eosin-5-Isothiocyanate (EY-ITC, Biotium 90091) (20 mg, 28.4 µmol, 1 eq) was reacted with tBu N-(3-aminopropoxy)carbamate (Ambeed A368728) (5.4 mg, 28.4 µmol, 1 eq) in the presence of DIPEA (14.8 µL, 85.1 µmol, 3 eq) in DMF (2 ml). The crude product was purified using HPLC (gradient from 100% of H_2O + 0.1% TFA to 80% of ACN+0.1% TFA) affording the thiourea product (22 mg, 24.6 µmol, 86.6% yield). Boc group was then deprotected in 50% TFA in DCM for 3 h, followed by evaporation on rotary evaporator and then overnight on Genevac to afford the TFA salt. The product was detected at 761.77 m/z $[M-34+1]^+$ corresponding to a carbodiimide desulfurization product, which was likely caused by Eosin Y-mediated oxidation of sulfur followed by elimination. Thus, the product was dissolved to 1 mM in wet DMSO with the intent to convert the carbodiimide into a stable urea.

Conjugation of WGA-EY: Wheat Germ Agglutinin (Vector Laboratories L-1020-25) was dissolved in PBS, desalted by PD-10 with PBS, then concentrated by Amicon 10K MWCO (EMD Millipore). Oxaziridine-azide in DMF was added to 5X molar excess and reacted for 2 hours at 4°C. Reaction was halted by removal of unreacted oxaziridine-azide via PD-10 column (Cytiva 17-0851-01), at which point protein was concentrated by Amicon 3K MWCO spin columns (EMD Millipore). Concentrated protein was reacted with 3X molar excess EY-DBCO overnight at 4°C. Excess unreacted EY-DBCO was removed by PD-10. Conjugation was confirmed by titration in NuPage 4-12% Bis-Tris gels alongside unconjugated protein, imaged by ChemiDoc (Bio-Rad) using the Alexa Fluor 568 imaging channel.

Conjugation of ConA-EY: Concanavalin A (Cayman C819Y48) was dissolved in PBS (Cytiva SH30256.FS), desalted by PD-10 column (Cytiva 17-0851-01) with PBS, then concentrated by Amicon 10K MWCO. Oxaziridine-azide in DMF was added to 5X molar excess and reacted for 2 hours at 4°C. Reaction was halted by removal of unreacted oxaziridine-azide via PD-10 column (Cytiva 17-0851-01) with PBS, at which point protein was concentrated by Amicon 3K MWCO spin columns (EMD Millipore). Concentrated protein was reacted with 3X molar excess EY-DBCO overnight at 4°C. Excess unreacted EY-DBCO was removed by Zeba Dye and Biotin removal columns (Thermo A44297) or PD-10. Conjugation was confirmed by LC-MS as well as titration in NuPage 4-12% Bis-Tris gels alongside unconjugated protein, imaged by ChemiDoc (Bio-Rad) in the Alexa Fluor 568 imaging channel.

Synthesis of Chol-EY: Cholesterol-TEG-azide (Sigma 794384) was dissolved in DMSO to 20 mM. Cholesterol-TEG-azide (1.5 mL, 30 µmol, 1.1 eq) was reacted with EY-DBCO (33.6 mg, 27.3 µmol, 1 eq) in 27 mL total DMSO, protected from light, for one week at room temperature. The reaction was periodically monitored by TLC with 20% MeOH/DCM with drying of TLC aliquots by Genevac prior to resuspension in DCM for loading. After one week, an additional 1.5 mL cholesterol-TEG-azide (1.5 mL, 30 µmol, 1.1 eq) was added to bring reaction to a final 2.2:1 molar ratio of cholesterol-TEG-azide:EY-DBCO. The crude reaction continued for two more days with TLC monitoring before being dried by rotovap and Genevac. The lipid was extracted by the

method described by Bligh and Dyer (4). Briefly, the dried crude was dissolved in 3.8 volume equivalents 1:2:0.8 chloroform:methanol:water with 0.2% (v/v) trifluoroacetic acid. To this was added one volume chloroform with mixing, then one volume water with mixing. The liquid was allowed to sit for 5 min protected from light to complete phase separation. The denser organic layer was collected and dried by rotary evaporation. The dried organic layer was dissolved in a small quantity of dichloromethane (DCM) and purified from the organic layer by normal phase liquid chromatography (NPLC) on Biotage Selekt with RediSep Silver Silica 12 g column (Teledyne ISCO) using 2 CV hexanes, followed by 3 CV dichloromethane (DCM), and a DCM:Methanol gradient up to 20% methanol over 11 CV. Fractions containing product were pooled, dried by Genevac and lyophilization. Target mass was detected at 1859 m/z by Acquity UPLC and Xevo QTOF-MS (Waters). The dried product was dissolved in DMSO to 1 mM stock.

Expression and purification of NbALFA: NbALFA coding sequence was cloned out of NbALFA-eGFP vector provided generously by Wooyoung Choi (Cheng Lab, UCSF) and inserted into a pFUSE periplasmic expression vector provided by Madison Seto (Wells Lab, UCSF) with stII signal peptide and C-terminal His-tag by Gibson cloning. An ectopic cysteine was added after the nanobody sequence. Plasmid was amplified in XL10 *E. coli* and sequence confirmed by nanopore sequencing. Protein was expressed periplasmically in C43 *E. coli* by previously published protocol (5) using autoinduction media. Cells were lysed with B-PER (Thermo PI78248) with cOmplete protease inhibitor cocktail (Roche 11836170001) and 1 μg/mL DNase I (Goldbio D-300-500) with probe sonication for 1 min with 5s/5s pulse at 20% amplitude. Lysate was clarified by 20,000 x g 20 min 4°C spin then filtered 0.45 μm. Clarified lysate was incubated overnight with nickel resin pre-washed with wash buffer (50 mM Tris pH 8.0, 200 mM NaCl, 20 mM imidazole). Resin was washed 5 times with 10 CV wash buffer, then the protein eluted with 3.5 CV elution buffer (50 mM Tris pH 8.0, 200 mM NaCl, 500 mM imidazole). The eluate was buffer exchanged into Tris pH 7.4 while concentrating by Amicon 3K MWCO (EMD Millipore). His-tagged TEV protease (UC Berkeley) was added at 1:20 by A280 and allowed to react overnight at 4°C. The reaction mixture was added to fresh nickel resin pre-equilibrated with wash buffer and allowed to incubate 2 hours at 4°C. The flowthrough was collected along with wash buffer washes, concentrated by Amicon 3K MWCO, and exchanged into Tris pH 7.4. Mass was confirmed by LC-MS on Acquity UPLC and Xevo QTOF-MS (Waters). The reverse-eluted protein was further purified by SD75 on Akta with buffer exchange and concentration into PBS.

Conjugation of NbALFA-EY: To NbALFA in PBS was added 10X TCEP pH 7.4 and 20X EY-maleimide (Biotium 92013). The reaction was allowed to occur on ice for 2 hours, after which unreacted EY-maleimide was removed by PD-10 column (Cytiva) with PBS and conjugated NbALFA-EY concentrated by Amicon 3K MWCO (EMD Millipore). Conjugation was confirmed by titration in NuPage 4-12% Bis-Tris gels alongside unconjugated protein, imaged by ChemiDoc using the Alexa Fluor 568 imaging channel.

Labeling of cells with EY conjugates

CSC-EY: K562 cells were harvested and washed with ice cold DPBS 3x at 200 x g 5 min 4°C. Cells were resuspended in 500 μM sodium periodate (Sigma 311448) in DPBS and incubated 15 min 4°C. Cells were then washed 3x with ice cold DPBS at 200 x g 5 min 4°C. Washed cells were resuspended in 50 μM aminooxy-EY with 1:1000 v/v aniline (Sigma 132934) in 1X PBS pH 6.5 (Teknova P0185). Cells were incubated for 10 min at 4°C then washed 3x with DPBS at 200 x g 5 min 4°C. For aminooxy-EY control experiments, the same protocol was used except the 500 μM sodium periodate incubation step used cold DPBS for a mock oxidation.

WGA-EY: K562 cells were harvested and washed with ice cold DPBS 3x at 200 x g 5 min 4°C. Cells were resuspended in 10 μM WGA-EY in DPBS and allowed to incubate 30 min at 4°C. Cells were then washed 3x with DPBS 200 x g 5 min 4°C.

ConA-EY: K562 cells were harvested and washed with ice cold DPBS 3x at 200 x g 5 min 4°C. Cells were resuspended in 10 µM ConA-EY in DPBS and incubated 30 min at 4°C. Cells were then washed 3x with DPBS 200 x g 5 min 4°C.

Chol-EY: K562 cells were harvested and washed with room temperature DPBS (Cytiva SH30028.FS) 3x at 200 x g 5 min. Cells were resuspended in 10 µM room temperature Chol-EY in DPBS and immediately transferred to a 37°C heatblock. After a 5 min incubation at 37°C, tubes containing cells were immediately placed into ice water and then spun down at 200 x g 5 min 4°C. Subsequently the cells were washed 3x with ice cold DPBS 200 x g 5 min 4°C.

NbALFA-EY: K562 cells were harvested and washed with ice cold DPBS 3x at 200 x g 5 min 4°C. For direct detection of EY (microscopy): Cells were resuspended in 10 µM NbALFA-EY in DPBS and allowed to incubate 30 min at 4°C. Cells were then washed 3x with DPBS 200 x g 5 min 4°C. For biotinylation (microscopy, flow cytometry, Western blot, proteomics): Cells were resuspended in 10 µM NbALFA-EY with 500 µM TFPA-PEG$_3$-biotin (Thermo 21303) in cold DPBS and allowed to incubate 30 min 4°C.

Cell surface biotinylation

Biotinylation by photo-PLP: Cells were labeled with EY conjugates as described above. Cells were then resuspended in 500 µM TFPA-biotin in DPBS and allowed to incubate 30 min at 4°C. Photocatalysis was performed by irradiation with green light at 4°C. For microscopy experiments: a PennPhD M2 photoreactor was used. A customized LED module with green LEDs (~520 nm maximum emission) was used. Cells were irradiated 10 min at 100% intensity. For proteomics, flow cytometry, and Western blot experiments: a PhotoRedOx Box (HepatoChem HCK1006-01-016) was used with green LED (HepatoChem HCK1012-01-004). A UV filter (Rosco #3114) was applied over the LED. Samples were irradiated for 5 min. After irradiation, cells were washed 3x with DPBS 200 x g 5 min 4°C. The pellet was snap frozen in liquid nitrogen (proteomics, Western blot) or the cells immediately processed (flow cytometry, microscopy).

Biotinylation by Cell Surface Capture: CSC was performed with minor adaptations from previously optimized protocols (6). Cells were harvested and washed with ice cold DPBS 3x at 200 x g 5 min 4°C. Cells were resuspended in 500 µM sodium periodate (Sigma 311448) in DPBS and incubated 15 min 4°C. Cells were then washed 3x with ice cold DPBS at 200 x g 5 min 4°C. Washed cells were resuspended in 500 µM alkoxyamine-PEG$_4$-biotin (Thermo 26137) with 1:1000 v/v aniline (Sigma 132934) in 1X PBS pH 6.5 (Teknova P0185). Cells were incubated for 10 min at 4°C then washed 3x with DPBS at 200 x g 5 min 4°C. Pellets were either snap frozen in liquid nitrogen (proteomics, Western blot) or immediately processed (flow cytometry, microscopy).

Biotinylation by WGA-HRP: Biotinylation was performed per previously published protocol (7). Cells were harvested and washed with cold PBS pH 6.5 3x at 400 x g 5 min 4°C. Per 1M cells, pellet was resuspended in 99 µL cold PBS pH 6.5. To cells was added 1 µL of diluted (2.05 µL vendor stock with 17.95 µL PBS pH 6.5) WGA-HRP (Vector PL-1026-02), then 1 µL 50 mM Biotin-AEEA-tyramide (Iris Biotech LS-3490.0100). Sample was mixed and placed on 37°C heat block. While on heat block, 1 µL of diluted hydrogen peroxide (8 µL 30% w/w H_2O_2 (Sigma H1009) added to 92 µL water) was added and mixed. The reaction was allowed to continue for 2 min at 37°C, at which point 25 µL 5X Quench (diluted in PBS pH 6.5 from 100X quench (198 mg sodium L-ascorbate (Sigma 11140) in 1 mL 100 mM sodium pyruvate (Sigma S8636))) was added. Cells were then spun at 400 x g 5 min 4°C and washed 3x with 1X Quench at 400 x g 5 min 4°C. Cells were washed once more with PBS pH 6.5 400 x g 5 min 4°C and the pellets either snap frozen in liquid nitrogen (proteomics, Western blot) or immediately processed (flow cytometry, microscopy).

Biotinylation by Sulfo-NHS biotin: Biotinylation was performed per previously published protocol (7) with minor adaptation. Cells were harvested and washed with twice with DPBS at 200 x g 5 min 4°C followed by 1X PBS pH 8.0 (Sigma PB180) at 200 x g 5 min 4°C. Cells were

resuspended in 500 µM Sulfo-NHS S-S biotin (Thermo 21331) in PBS pH 8.0 and incubated 30 min 4°C. Cells were then washed 3x with DPBS 200 x g 5 min 4°C and snap frozen in liquid nitrogen (proteomics, Western blot) or immediately processed (flow cytometry, microscopy).

Microscopy

Confocal microscopy for detection of Eosin Y: EY-conjugate labeled cells without biotinylation or photoirradiation were added onto poly-L-Lysine treated 8-well microscopy slides (ibidi 80804) at 100K cells/well in 200 µL DPBS. The plate was then spun at 400 x g for 30 min at 4°C. Adhered cells were then fixed in the wells with ice cold 4% paraformaldehyde (Electron Microscopy Sciences 15710) in DPBS and allowed to come to room temperature over 10 min. Cells were then incubated in 100 mM glycine in DPBS for 15 min at RT, then washed 2x with room temperature DPBS. Cells were then stained with 1 µg/mL Hoechst (Invitrogen H1399) in DPBS for 10 min at RT. Cells were washed 3x with room temperature DPBS then imaged on a Nikon Ti inverted microscope with Yokogawa CSU-22 Spinning Disk Confocal. Laser intensities were: 405 (DAPI) 20%, 488 (GFP) 50%. Exposure times were: 405 (DAPI) 200 ms, 488 (GFP) 300 ms, BF 300 ms. Images were processed in FIJI. Brightness and contrast were adjusted in each channel per image independently to highlight distribution.

Confocal microscopy for detection of biotinylation: Washed biotinylated cells were added onto poly-L-Lysine treated 8-well microscopy slides (ibidi 80804) at 100K cells/well in 200 µL cold DPBS. The plate was then spun at 400 x g for 30 min at 4°C. Adhered cells were then fixed in the wells with cold 4% paraformaldehyde in DPBS (Electron Microscopy Sciences 15710) then warmed to room temperature over 10 min. Cells were washed 3x with room temperature DPBS then incubated in 3% BSA, 100 mM glycine, 0.1% Tween-20 in DPBS overnight at 4°C or for 1 hour at room temperature. Cells were next stained with 1:500 Streptavidin-Alexa Fluor 647 (Thermo S21374) in 3% BSA, 100 mM glycine, 0.1% Tween-20 in DPBS overnight at 4°C or for 1 hour at room temperature. Unbound fluorophore was washed away with 3 washes of DPBS 0.05% Tween-20 before staining with 1 µg/mL Hoechst (Invitrogen H1399) in DPBS for 10 min at RT. Cells were washed 3x with room temperature DPBS then imaged on a Nikon Ti inverted microscope with Yokogawa CSU-22 Spinning Disk Confocal. Laser intensities were: 405 (DAPI) 30% and 640 (Cy5) 15%. Exposure times were: 405 (DAPI) 200 ms, 640 (Cy5) 300 ms, BF 100 ms. Images were processed in FIJI. Brightness and contrast were adjusted in each channel per image independently to highlight distribution.

Confocal microscopy for detection of cell surface localized proteins with commercial antibodies: Washed cells were incubated with 1:1000 (v/v) Live or Dead 488/515 (Biotium 32004A) in cold DPBS for 15 min at 4°C. Cells were washed twice with cold 0.22 µm filtered DPBS 1% (w/v) BSA 1 mM EDTA (Flow buffer) 200 x g 5 min 4°C. Cells were then resuspended in 1:5 (v/v) FcR Blocking Reagent Human (Miltenyi Biotec 130-059-901) in cold 0.22 µm filtered 3% (w/v) BSA in DPBS and allowed to block at 4°C for 20 min. Subsequently 1:100 (v/v) primary antibody (Proteintech; see commercial primary antibodies table) in 3% BSA DPBS was added at equal volume to a final 1:200 (v/v) concentration. Primary antibody incubated for 1 hour at 4°C, then cells were washed 3x with flow buffer at 200 x g 5 min 4°C. Cells were resuspended in 1:200 (v/v) multi-rAb CoraLite Plus 647 goat anti-rabbit (Proteintech RGAR005) in cold 0.22 µm filtered 3% BSA in DPBS and incubated for 1 hour at 4°C covered from light. Following 3x washes with cold DPBS, cells were brought up in 1 mL cold DPBS and plated at 50K cells in 100 µL cold DPBS per well on poly-L-Lysine treated 8-well microscopy slides (ibidi 80804). The plate was then spun at 400 x g for 30 min at 4°C. Adhered cells were then fixed in the wells by adding 100 µL cold 4% paraformaldehyde (PFA) in DPBS (Electron Microscopy Sciences 15710) for a final 2% PFA. The cells were allowed to warm to room temperature over 10 min. Fixation was halted by addition of 200 µL 200 mM glycine in DPBS and incubation for 15 min at RT. Cells were washed 3x with room temperature DPBS then counterstained with 1 µg/mL Hoechst (Invitrogen H1399) in DPBS for 10 min at RT. Cells were washed 3x with room temperature DPBS then imaged on a Nikon Ti inverted microscope with Yokogawa CSU-22 Spinning Disk Confocal. Laser intensities were: 405 (DAPI) 30%, 488 (GFP) 20%, and 640 (Cy5) 15%. Exposure times were:

405 (DAPI) 200 ms, 466 (GFP) 300 ms, 640 (Cy5) 300 ms, BF 100 ms. Images were processed in FIJI. Brightness and contrast were adjusted in each channel per image independently to highlight distribution.

Flow cytometry

Flow cytometry for detection of surfaceome biotinylation: Washed biotinylated K562 cells were fixed with 2% PFA in cold DPBS for 10 min and allowed to come to room temperature. Fixed cells were washed with 1% BSA 1mM EDTA DPBS (flow buffer) at 400 x g 5 min 4°C and either immediately stained or stored at 4°C protected from light overnight. Washed cells were then incubated in 1:1000 Streptavidin-Alexa Fluor 647 (Thermo S21374) in flow buffer for 30 min at 4°C, then washed 3x with flow buffer at 400 x g 5 min 4°C. Cells were resuspended in flow buffer, strained with cell strainer (Corning 07201430) and measured on CytoFLEX (Beckman Coulter). Data were processed in FlowJo (Waters/BD Biosciences) with gating for single cells.

Flow cytometry for detection of cell surface localized proteins with commercial antibodies: K562 cells were washed with ice cold DPBS 3x at 200 x g 5 min 4°C. Cells were then incubated in 100 µL of fifty-fold dilution of MonoZero Human Fc Blocking Reagent (Proteintech PF00032) in 1% BSA 1mM EDTA DPBS filtered 0.22 µm (flow buffer) at 4°C for 15 min. To this was then added 100 µL of 20 µg/mL primary antibody (Proteintech; see commercial primary antibodies table) for a final 10 µg/mL. Cells were incubated in primary for 30 min at 4°C. Cells were then washed 3x with flow buffer at 200 x g 5 min 4°C, then stained with 1:500 multi-rAb CoraLite Plus 647 goat anti-rabbit (Proteintech RGAR005) in flow buffer for 30 min at 4°C. Cells were washed again 3x with flow buffer at 200 x g 5 min 4°C, resuspended in 1:5000 Sytox Blue (Life Technologies S34857) in flow buffer for 15 min at 4°C, then measured on CytoFLEX (Beckman Coulter) using QC gains. Data were processed in FlowJo (Waters/BD Biosciences) with gating for single cells and live cells.

Commercial Primary Antibodies

**Target**	**Vendor**	**Product**	**Concentration**	**Use**
LYN	Proteintech	18135-1-AP	10 µg/mL	FC, ICC
MARCKSL1	Proteintech	11422-1-AP	10 µg/mL	FC, ICC
SRC	Proteintech	11097-1-AP	10 µg/mL	FC, ICC
CFH	Proteintech	12748-1-AP	10 µg/mL	FC
HNRNPU	Proteintech	14599-1-AP	10 µg/mL	FC
NF1	Proteintech	27249-1-AP	10 µg/mL	FC
KMT2C	Proteintech	28437-1-AP	10 µg/mL	FC
FN1	Proteintech	15613-1-AP	10 µg/mL	FC
PLK1	Proteintech	10305-1-AP	10 µg/mL	FC
SNRNP200	Proteintech	23875-1-AP	10 µg/mL	FC
MYL6B	Proteintech	16963-1-AP	10 µg/mL	FC, ICC
RACGAP1	Proteintech	13739-1-AP	10 µg/mL	FC, ICC
TOP2A	Proteintech	24641-1-AP	10 µg/mL	FC, ICC
SPB5	Proteintech	11722-1-AP	10 µg/mL	FC, ICC

Flow cytometry for detection of IO proteins with high quality binders: K562 cells (ATCC) were cultured in IMDM media (Gibco 12440046) supplemented with 10% fetal bovine serum and 1% penicillin/streptomycin. Before antibody staining, cells were incubated with Human TruStain FcX™ Fc Receptor Blocking Solution (BioLegend 422301) according to the manufacturer's instructions to reduce non-specific antibody binding. Cells were then stained with 100 nM IgG for 30 min on ice, washed three times with PBS containing 1% BSA, and detected using AffiniPure Goat Anti-Human IgG F(ab')$_2$ (Jackson ImmunoResearch 109-605-088; 1:400 dilution in PBS 1% BSA) for 20 min on ice. Following three additional washes with PBS/1% BSA, data were acquired

on a CytoFLEX flow cytometer (Beckman Coulter) and analyzed using FlowJo v10.10.0 (Waters/BD Biosciences).

Preparation of biotinylated cell pellets for Western blot and proteomics: Frozen cell pellets (10M cells) were thawed with 250 µL ice cold 1X RIPA (Millipore 20-188) supplemented with 1 tablet/10 mL cOmplete protease inhibitor cocktail (Roche 11873580001) and 1 mM EDTA pH 8.0 (Sigma 03690) on ice. Cells were mixed in lysis buffer by vortexing then sonicated 1 min 20% amplitude with 5s/5s pulse by probe sonicator at 4°C in ice water slurry. Lysates were clarified by centrifugation at 20000 x g 15 min 4°C. The clarified lysate was transferred to new Axygen Maxymum Recovery homopolymer tubes (Corning MCT-150-L-C) and the total protein quantified by the Pierce 660 nm protein assay kit (Thermo 22662) with ionic detergent compatibility reagent (Thermo 22663) per manufacturer ratios.

Western blot: 20 µg of lysate as quantified by Pierce 660 nm protein assay kit (Thermo 22662) with ionic detergent compatibility reagent (Thermo 22663) was mixed with LDS Sample Buffer (Thermo NP0007) to 1X. Samples were not incubated at 95°C to avoid false thermally-induced biotinylation by residual unreacted probe. Samples were loaded on BOLT 4-12% Bis-Tris gels (Thermo NW04125) and run 30 min 200 V in 1X MES. The run gel was transferred by iBlot 2 (Thermo) with PVDF membrane (Thermo IB24002). Membrane was washed 3x with TBST then blocked in Intercept TBS blocking buffer (LICORbio 927-60001) for 1 hour at room temperature or overnight at 4°C. Blocked membrane was washed 3x with TBST then incubated with 1:5000 IRDye 800CW Streptavidin (LICORbio 926-33230) in Intercept with 0.1% SDS (Fisher BP166). Stained membrane was washed 3x with TBST then imaged on Odyssey (LICORbio).

Development of optimized enrichment proteomics protocol

Original protocol: We originally employed a biotin-enrichment protocol based on previously published protocol from our lab (2, 8). Briefly, frozen biotinylated cell pellets were lysed in 2X RIPA with protease inhibitors with 1 mM EDTA. Resuspended pellets were sonicated by probe sonication 20% A 5s/5s 1 min on ice, then allowed to sit on ice 10 min. The supernatant was clarified at 20,000 x g 4°C. High capacity NeutrAvidin agarose resin (Thermo 29204) was washed with 1X RIPA 1 mM EDTA three times. 100 µL of slurry was then added per sample, mixing at 4°C for 120 min within a plugged and capped chromatography column (Bio-Rad 7326008). After incubation, the resin was cleaned by vacuum filtration with washes at 42°C of 5 mL 1X RIPA 1 mM EDTA, 5 mL PBS 1 M NaCl, then 5 mL 50 mM ammonium bicarbonate (ABC) with 2 M urea. The washed resin was recovered into a 1.5 mL Axygen tube using 100 µL PreOmics LYSE, then spun on a benchtop centrifuge to pellet the resin. LYSE supernatant was removed for a total volume of 50 µL. The samples were resuspended then incubated 55°C 10 min with 1000 rpm shaking. After cooling to RT, to this was added 50 µL DIGEST (210 µL PreOmics RESUSPEND added per tube). Samples were allowed to digest for 90 min at 37°C with 500 rpm shaking. The entirety of the sample was then transferred to spin columns (Thermo 69725) and the solution separated by spinning at 500 x g 5 min RT. To this was added 100 µL STOP. Alternatively, the STOP was added prior to separation of the resin. The solution was then bound to PreOmics desalting columns by spinning at 3800 x g 3 min, then washed with 200 µL WASH 1 and 200 µL WASH 2 at 3800 x g 3 min 4°C. The washed column was transferred to a fresh low binding Axygen tube and elute with 2 x 100 µL 3800 x g 3 min 4°C washes. Peptides were dried by Genevac.

Optimization around native capture: We also tested lysis conditions including 1X RIPA, 1X RIPA 1% SDS as employed in multiple µMap reports (9, 10), and 2X RIPA 1% SDS. We did not observe a notable difference across these lysis buffer conditions in terms of number of detected proteins and thus utilized 1X RIPA with 1 mM EDTA and cOmplete protease inhibitor. While some previous publications from our lab allowed the biotinylated lysate to bind the resin overnight (2), we restricted this to 90 minutes to decrease nonspecific binding. We also tested varying strength washes including 2 M urea, 4 M urea, and 8 M urea. As we did not notice a disadvantage to 8 M

urea, we kept this high stringency wash. However, using this optimized old protocol, we still detected large numbers of proteins in non-biotinylated samples indicative of high adsorption or insufficient washing, which we reasoned could be due to protein aggregation and nonspecific but irreversible binding to the beads and/or biotinylated protein.

We were concerned that the column washing protocol may be more susceptible to batch effects, particularly if the wash buffers did not adequately permeate the agarose resin. We thus attempted to perform the same protocol with washing in Axygen tubes, using centrifugation to collect the resin. Concerningly, we noted that after urea washes, the resin no longer pelleted. We attribute this to unfolding of bead-bound protein and aggregation that traps gas. We also noted that the agarose resin is porous, meaning that the wash should ideally permeate the pores for adequate washing, but that high viscosity washes like urea washes may struggle to permeate and thus not clean the beads. Due to this technical handling issue and the risk of inadequate washing due to agarose porosity, we investigated alternative enrichment supports.

Optimization around bead enrichment support: As the use of small magnetic particles is becoming the standard in PLP, we tested multiple forms of small magnetic biotin capture particles (Pierce Streptavidin Magnetic Beads, Thermo 88817; Sera-Mag SpeedBeads Neutravidin-Coated Magnetic Particles, Cytiva 78152104011150; Sera-Mag SpeedBeads Streptavidin-Blocked Magnetic Particles, Cytiva 21152104010150). We tested these using both our existing protocol as well as the µMap washing protocol (3x with DPBS 1% SDS, 3x with DPBS 1 M NaCl, and 3x with DPBS 10% EtOH). Regardless of washing protocol, we noted that the beads would frequently film against the tube and a large proportion would thus become unrecoverable. This was more noticeable for the Pierce beads. A floating film was also noticeable—most notably after any urea wash, but also after high salt washes—that could not be attracted by magnet or spun to the bottom of the tube. We suspect that this film is similarly the result of protein aggregation and the entrapment of gas.

Optimization including denaturing biotin capture: Because we wondered whether the sudden introduction of chaotrope would cause rapid and irreversible adsorption of denatured protein onto the bead support, we tested approaches that would allow protein to bind to the Streptavidin or Neutravidin in denaturing conditions. We thus tested protein precipitation by Wessel-Flügge (11) followed by resuspension in 4 M urea with 0.5% SDS that has been previously used for Streptavidin binding (12). The use of Wessel-Flügge has the added benefit of removing biotinylated lipids, which if present may compete with biotinylated proteins for binding sites on the beads. This was a particular concern because the binding capacity of the small magnetic particles is orders of magnitude lower than that of the high capacity NeutrAvidin agarose (55 µg biotinylated protein/mg beads, 1% v/v solids compared to 8 mg biotinylated protein/mL slurry). We reasoned such conditions would not prohibit the binding of biotin to Streptavidin while keeping the proteins denatured (to prevent retention of interacting proteins) and in conditions that do not promote aggregation, allowing us to then use high urea washes without a sudden aggregation cascade. Given this starting point, we were able to use 8 M urea 1% SDS with 50 mM Tris pH 8.0 as our strongest washes, which is far stronger than the strongest 1X RIPA 1% SDS or 50 mM ABC 8 M urea washes used in the PLP field.

To determine the best enrichment conditions, we performed CSC in technical replicates and treated each sample with a different enrichment protocol. We observed superior performance in terms of higher GOCCPM fractional intensity and decreased nonspecific protein detection when using the Sera-Mag Streptavidin-Blocked Magnetic Particles and the denaturing protocol.

Explanation of bead volume: The amount of beads used in this study to capture biotinylated protein is in excess of what is necessary for complete capture. We are relatively certain of this given our internal testing, and a caveat is that with higher bead surface area comes a greater risk of nonspecific adsorption. In this study we have chosen to navigate an avenue where an excess of bead is used, followed by extremely stringent washing, as opposed to risking incomplete biotinylated protein capture.

The amount of beads used in this study for surfaceomics is on the same order of magnitude as published targeted PLP studies, which we expect achieve less biotinylation than surfaceomics due to a constrained target pool. For reference, the original μMap paper (9) used 250 μL of Pierce Streptavidin magnetic beads (55 μg biotinylated rabbit IgG/mg beads, 1% (v/v) beads) per 1 mg lysate biotinylated by targeted Iridium-activated diazirines, which are far less efficient at protein insertion than TFPA activated by EY. In the original MultiMap paper (2), 200 μL of NeutrAvidin agarose beads (8 mg biotinylated BSA/mL) was used without considering protein lysate amount. The vendor reported binding capacity for the SeraMag SpeedBeads Streptavidin-Blocked Magnetic Particles used in this study is 29.9 μg biotinylated BSA/mg (1% (v/v) beads) or 58.9 μg biotinylated IgG/mg beads.

Using the estimate of 1% of total protein being on the cell surface (13), we estimated ~2.5 μg surfaceome-derived protein in 250 μg of total cell lysate. This does not account for endogenously biotinylated proteins or off-target biotinylation. Since 100 μL of beads should bind ~29.9 μg biotinylated protein, this is a ~10X excess over the entirety of the surfaceome and presumably safe for complete capture. We empirically performed a titration of beads against WGA-HRP biotinylated samples in preliminary data to determine how much bead is needed. By dot blot of the supernatant after incubation with the streptavidin magnetic particles, we estimated that a 1:20 beads:total lysate ratio was at the border of complete biotin capture.

Given this empirical finding, we initially considered using a smaller amount of beads more consistent with 1:20 than 10:1. Ultimately, we stuck with the 10:1 ratio due to a combination of similarity to literature precedence and technical handling. As streptavidin magnetic particles can—even independently of protein binding—stick to the sides of low-bind plastic tubes over the course of sample manipulation or lose strong attachment to magnet, we reasoned that starting with a small volume of beads presented a real risk of complete sample loss. Under manual handling, we determined that 100 μL beads is tractable and very safe when handling large numbers of samples, although for more targeted experiments with smaller sample sizes we recommend 50 μL of beads for manual handling.

Proteomics sample preparation and data collection

Proteomics preparation of peptides using streptavidin magnetic bead-enrichment: For rationale in development of this protocol see Extended Methods. 250 μg of total protein lysate as quantified by Pierce 660 nm protein assay kit (Thermo 22662) with ionic detergent compatibility reagent (Thermo 22663) was aliquoted from each sample and diluted with complete lysis buffer (1X RIPA 1 mM EDTA with cOmplete) to the next 100 μL in either an Axygen 1.5 mL Maxymum Recovery tube or 15 mL Falcon tube. Protein was precipitated with methanol and chloroform as described by Wessel and Flügge (11). Per 100 μL lysate, 400 μL methanol, 100 μL chloroform, and 300 μL water were added sequentially with mixing. The mixtures were spun down at 10000 x g 5 min at room temperature (1.5 mL) or 4000 x g 10 min at room temperature (15 mL). The liquid layers were aspirated and the protein wafer gently washed with 1:1 methanol:chloroform, then spun again. All organic liquid was aspirated and the pellets allowed to briefly dry by keeping tubes open within the chemical fume hood.

To each dried wafer 250 μL of 8M urea 1% SDS 100 mM Tris pH 8.0 was added. The wafer was resuspended by pipetting using low retention tips and the suspension transferred to a low bind tube, if previously in a 15 mL tube, and the tubes parafilmed shut. The protein was resuspended by bath sonication for 30 min, with ice added periodically to the water bath to prevent excessive heating. After sonication, the resuspended proteins were transferred to new Maxymum Recovery tubes and diluted with 250 μL 100 mM Tris pH 8.0. To each sample was added 100 μL of SeraMag SpeedBeads Streptavidin Blocked Magnetic Beads (Cytiva 21152104010350) that were freshly pre-equilibrated with 4 M urea 0.5% SDS 100 mM Tris pH 8.0 and reconstituted with the starting volume. Samples were allowed to incubate with beads overnight at room temperature on a rotisserie.

Following incubation, beads were pelleted on a magnetic rack (Thermo 12321D) and the supernatant removed. Beads were washed with 1 mL 4 M urea 0.5% SDS 100 mM Tris pH 8.0 and transferred to a new Maxymum Recovery tube using low retention tips. Next 1 mL 8 M urea 1% SDS 100 mM Tris pH 8.0 was added and the beads thoroughly washed; this wash was repeated for a total of three times, then the beads transferred to a fresh Maxymum Recovery tube by low retention tips. The beads were washed with 1 mL 8 M urea 0.05% SDS 100 mM Tris pH 8.0 then resuspended in 1 mL 50 mM ammonium bicarbonate (ABC) with 0.05% SDS for transfer to a fresh Mayxmum Recovery tube. Beads were washed one final time with 50 mM ABC, the ABC aspirated, and then the beads spun to the bottom of the tube by benchtop centrifuge.

Beads were then processed by PreOmics iST 96x (PreOmics P.O.00027) with on-bead digestion. To the washed beads was added 50 µL of PreOmics LYSE. Beads were resuspended by pipetting using low retention tip, then incubated at 55°C for 10 min with 1000 RPM shaking. The mixture was then allowed to cool to room temperature, at which point 50 µL of DIGEST prepared in RESUSPEND per manufacturer directions was added and mixed by low retention tip. The beads were incubated at 37°C for 90 min with 900 RPM shaking, with periodic vortexing to prevent settling. At the conclusion of incubation, 100 µL STOP was added and thoroughly mixed. The samples were spun at 600 x g for 5 min at room temperature then the supernatant collected while on magnetic rack. Supernatant was loaded in PreOmics columns over 2 mL collection tubes and spun at 2250 x g 3 min 4°C. Columns were then washed with 200 µL each of WASH 1 and WASH 2 sequentially at 2250 x g 3 min 4°C. Cleaned columns were transferred over fresh Maxymum recovery tubes and the peptides collected via two rounds of 100 µL ELUTE at 2250 x g 3 min 4°C. Peptides were dried by Genevac and stored at -80°C or immediately processed for loading onto timsTOF Pro 2.

Preparation of whole cell proteomics samples: Samples were prepared per the PreOmics iST 96x protocol. To each frozen cell pellet (10M cells) was added 300 µL of PreOmics LYSE. Sample was resuspended and incubated at 95°C for 10 min with 1000 RPM shaking. The lysed sample was then sonicated 1 min 20% amplitude 5s/5s pulse. Sample was again incubated at 95°C for 10 min with 1000 RPM shaking. After cooling to room temperature, 50 µL of sample was taken into subsequent steps. 50 µL of DIGEST in RESUSPEND was added per sample, mixed, and incubated 90 min at 37°C with 900 RPM shaking. The reaction was stopped by 100 µL STOP. The mixture was then transferred to PreOmics column over a 2 mL microtube and spun at 2250 x g 3 min 4°C, followed by 200 µL WASH 1 and WASH 2 with the same spin parameters. The column was transferred to a new Maxymum Recovery tube and peptides eluted with 2 rounds of 100 µL ELUTE at 2250 x g 3 min 4°C, at which point they were dried by Genevac and either stored at -80°C or immediately processed for loading.

Preparation of BG negative control samples: Bead BG samples were prepared by harvesting an identical number of K562 cells as for surfaceomics. Cells were washed 3x with DPBS 200 x g 5 min 4°C and allowed to sit in DPBS on ice for the same length of time as the remainder of a photo-PLP surfaceomics labeling procedure. The pellet was snap frozen in liquid nitrogen and lysed as described above in “Preparation of biotinylated cell pellets for Western blot and proteomics”. TFPA BG samples were prepared by harvesting an identical number of K562 cells as for surfaceomics. Cells were washed 3x with DPBS 200 x g 5 min 4°C and allowed to sit in DPBS on ice for the same length of time as in a photo-PLP surfaceomics labeling procedure prior to TFPA-biotin incubation. Cells were then resuspended in 500 µM TFPA-biotin in DPBS and allowed to incubate 30 min at 4°C. Cells were then irradiated with green light at 4°C by PhotoRedOx Box (HepatoChem HCK1006-01-016) with green LED (HepatoChem HCK1012-01-004). A UV filter (Rosco #3114) was applied over the LED. Samples were irradiated for 5 min. After irradiation, cells were washed 3x with DPBS 200 x g 5 min 4°C. The pellet was snap frozen in liquid nitrogen and lysed as described in “Preparation of biotinylated cell pellets for Western blot and proteomics”.

Preparation of peptides for injection on timsTOF Pro 2: Dried peptides were spun at 15000 x g 5 min 4°C then 11 µL of LC-LOAD was added. Samples were vigorously vortexed then allowed to incubate for at least 90 min at 4°C. Samples were then vortexed again, spun down by benchtop centrifuge, mixed, and 1 µL of each sample quantified using the Pierce Quantitative Colorimetric Peptide Assay (Thermo 23275) preserving manufacturer ratios of sample to working reagent. The desired amount of peptide was then aliquoted and diluted with LCMS-grade 2% acetonitrile 0.1% formic acid in water to either 200 ng/µL, 100 ng/µL, or 50 ng/µL in Maxymum Recovery tubes. Samples were clarified by 20000 x g 10 min 4°C then the supernatant loaded into SureSTART 0.2 mL TPX screw top microvials (Thermo 60180-1655) with SureSTART screw caps (Thermo 6PSC9ST101).

Peptide fractionation for library construction: Fractionation of pooled peptides was performed by Pierce High pH Reversed-Phase peptide fractionation kit (Thermo 84868). 400 ng of peptide from each enriched sample was pooled for one fractionation, and whole cell lysate biologic replicates were pooled for a second fractionation. A minimum of 10 µg starting peptide was used for each fractionation. Pooled peptides were dried by Genevac and processed per kit instructions. Each fraction was then dried by Genevac and processed for injection as described above.

DDA-PASEF: For each sample 200 ng peptide was injected on Bruker timsTOF Pro 2. Starting solvent A was 2% ACN 0.1% FA (mix of Fisher LS118-1 and Fisher LS120-1), and solvent B was ACN 0.1% FA (Fisher LS120-1). Peptides were separated and eluted by a 30 min gradient from 2% to 28% B, followed by a step increasing to 32% B at 32 min, a rapid rise to 95% B at 32.5 min, and washing out at 95% B until 45 min. The flow rate was 0.5 µL/min and the ion mobility window ($1/K_0$ width) 0.015 $V{\cdot}S/cm^2$.

DIA-PASEF: For each sample 200 ng peptide was injected on Bruker timsTOF Pro 2. Starting solvent A was 2% ACN 0.1% FA (mix of Fisher LS118-1 and Fisher LS120-1), and solvent B was ACN 0.1% FA (Fisher LS120-1). Peptides were separated and eluted by a 30 min gradient from 2% to 28% B, followed by a step increasing to 32% B at 32 min, a rapid rise to 95% B at 32.5 min, and washing out at 95% B until 45 min at a flow rate of 0.5 µL/min. DIA-PASEF windows were acquired across an m/z range of 100-1700 Th, coupled to an ion mobility range of $1/K_0$ = 0.7 – 1.3 $V{\cdot}S/cm^2$.

Proteomics data analysis

Proteomics data processing: DIA-PASEF samples were processed using Spectronaut (Biognosys) (14) using a library generated from the DDA-PASEF files of fractionated samples via Pulsar, matched to the reviewed SwissProt human proteome (15) with common contaminants retrieved with FragPipe v23.0 and the sequence of full length streptavidin (UniProt P22629). Spectronaut default settings were used with changes to remove cross-run normalization and quantify protein groups using Quant 2.0 instead of MaxLFQ due to expected sample dissimilarity between enriched surfaceome samples, negative controls, and unenriched whole cell lysate.

Proteomics data analysis: The code used for analysis is available on GitHub, viewable via https://gitfront.io/r/wwho99/U4MVEQvX7zQi/Comparative-Analysis-Canonical-and-Noncanonical-Surfaceomes/. Common DIA-PASEF contaminants were filtered out using the list published by Frankenfield et al. (16). A criterion of quantitation in 2/3 biologic replicates was enforced for detection of an elution group within a condition. Canonical CSPs were determined per the SURFY list published by Bausch-Fluck et al. (17) and the Gene Ontology Cellular Compartment: Plasma Membrane annotation (18, 19). In cases where Spectronaut attributed an elution group to a protein group, that protein group was considered to belong to these lists if any protein of the group was found in the list.

For peptide level analysis, non-imputed data were used for absolute quantitative analyses such as intensity fractions attributable to reference lists. For heatmap and principal component analysis (PCA) visualizations, elution group total intensities were $\log_2$ transformed and missing not at random (MNAR) imputation drawn from a normal distribution with a 1.8 downshift and 0.3 width, as is the default in Perseus (20), was used for missing values. PCA was generated with scikit-learn (21).

For protein group level analysis, only protein groups with at least two unique peptides were retained for analysis. Intensities were $\log_2$ transformed then normalized on a per-sample basis to the median intensity of known endogenously biotinylated carboxylases (22, 23), after which these carboxylases were filtered out (endogenous biotin normalization) or to the median intensity of the entire sample (median normalization). MNAR imputation was then performed from a left shifted normal distribution using the Perseus default parameters of 1.8 downshift and 0.3 width. Non-imputed data were used for absolute quantitative analyses and set analyses. Imputed data were used for PCA and heatmap visualization as well as differential expression analysis. Differential expression analysis was performed with limma (24) with imputed values passed with a weight of 0.01 and measured values a weight of 1.0. Benjamini-Hochberg correction (25) was used to generate FDR-adjusted p-values.

Gene set enrichment analysis: Gene set enrichment analysis was performed using GSEApy (26) with a negative enrichment score minimum cutoff of 1.5 and FDR-adjusted q-value maximum cutoff of 0.25. Gene Ontology terms with semantic similarity greater than 0.7 were collapsed by GOSemSim using the graph based method (27, 28). Bioinformatics analyses were performed using annotations from Gene Ontology (GO:BP, GO:CC, GO:MF) (18, 19), Reactome (29), and KEGG (30, 31). For plots, only terms from GO: Cellular Compartment and GO: Biological Process were used.

Glycosylation analysis: Glycosylation annotations were retrieved from UniProt (15) entries and classified into categories by regular-expression (regex) matching of feature descriptions: N-linked/N-glycosylation, O-linked/O-glycosylation, O-GlcNAc (including O-linked GlcNAc phrasing), C-linked glycosylation, and other sugar-related modifications (phospho-, GlcNAc, xylose, mannose, fucose, galactose, glucose). Proteins with an N-glycosylation regex term either from experimental evidence or predicted were considered to be N-glycosylated.

Enrichment analysis by rank permutation: Enrichment algorithm was inspired by RankProd (32). Enriched samples were compared to unenriched whole cell lysate samples pairwise, restricting only to protein groups that were detected in at least 2 replicates of the enriched condition. For each PG, the difference in endogenous biotin-normalized log2 intensity was computed for every enriched–unenriched replicate pair. Within each pairing, proteins were then ranked by descending $\log_2$ difference, wherein rank 1 had the highest increase in the enriched condition. In cases where $\log_2$ differences were identical, the average rank was assigned to relevant ties. An observed enrichment score was defined as the geometric mean of these ranks across all pairwise comparisons. Significance was assessed by a null hypothesis random permutation. Independent random permutations of ranks 1 to N (where N is the number of analyzed proteins) were generated for each pairwise comparison over 1000 iterations. The geometric mean of these permuted ranks was calculated to build a pooled null distribution of $1000 \times N$ values under the hypothesis that rank order is random. Subsequently, for each protein, empirical FDR was estimated as the proportion of values from the null distribution less than or equal to the observed score. Proteins fulfilling FDR < 0.05 were considered significantly enriched.

Enrichment analysis by robust z-score: $\log_2$ fold change between each enriched condition and unenriched whole cell lysate was calculated from the mean endogenous biotin-normalized log2 intensities. Using protein groups that were quantified in at least 2 replicates of an enriched condition, median $\log_2$FC and median absolute deviation (MAD) of each pairwise condition comparison were computed, with MAD scaled with the consistency constant 1.4826 to approximate a standard deviation under normality (24). A robust z-score was calculated for all

detected proteins as (log_2FC − median) / (MAD x 1.4826), with MAD floored at 1 x 10^{-9} to avoid division by zero. Proteins fulfilling robust z-score magnitude greater than 2 were considered significantly enriched.

Schematics: Schematic figures were created with Biorender.com using either published or predicted structures. Illustration is not to scale and is strictly for visualization. Structure of WGA: PDB 7WGA (33). Structure of ConA: PDB 1TEI (34). Membrane protein structures were generated in AlphaFold 3 (35) and rendered in ChimeraX (36). UCSF ChimeraX was developed by the Resource for Biocomputing, Visualization, and Informatics at the University of California, San Francisco, with support from National Institutes of Health (NIH) R01-GM129325 and the Office of Cyber Infrastructure and Computational Biology, National Institute of Allergy and Infectious Diseases.

Figures

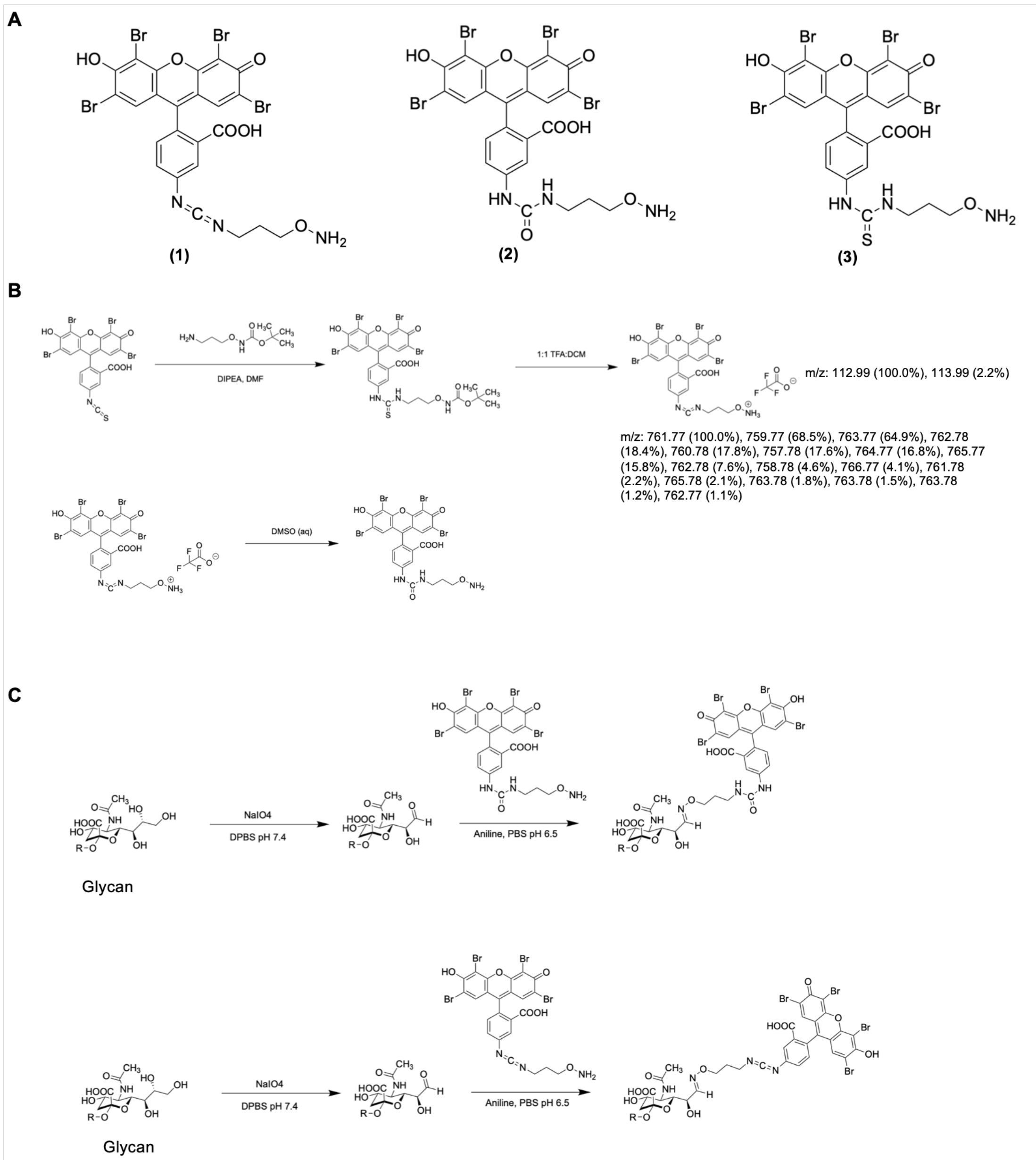

Fig. S1. Preparation of aminooxy-EY for CSC-EY. (**A**) Structure of aminooxy-EY as synthesized **1**, stable hydrolyzed product **2**, and product as intended **3**. Over the course of synthesis desulfurization was noted, potentially catalyzed by Eosin Y itself, yielding a carbodiimide in the linker. This linker variation is not expected to impact photocatalytic activity. (**B**) Synthetic scheme to generate aminooxy-EY **1**. **1** was allowed to incubate in DMSO that had previously been exposed to standard lab environment, protected from light, over multiple days at room temperature for intended hydrolysis into the stable urea linked **2**. However, carbodiimide **1** was still detected by mass spectrometry, suggesting low reactivity with water. (**C**) Conjugation scheme for attaching aminooxy-EY to cell surface glycans.

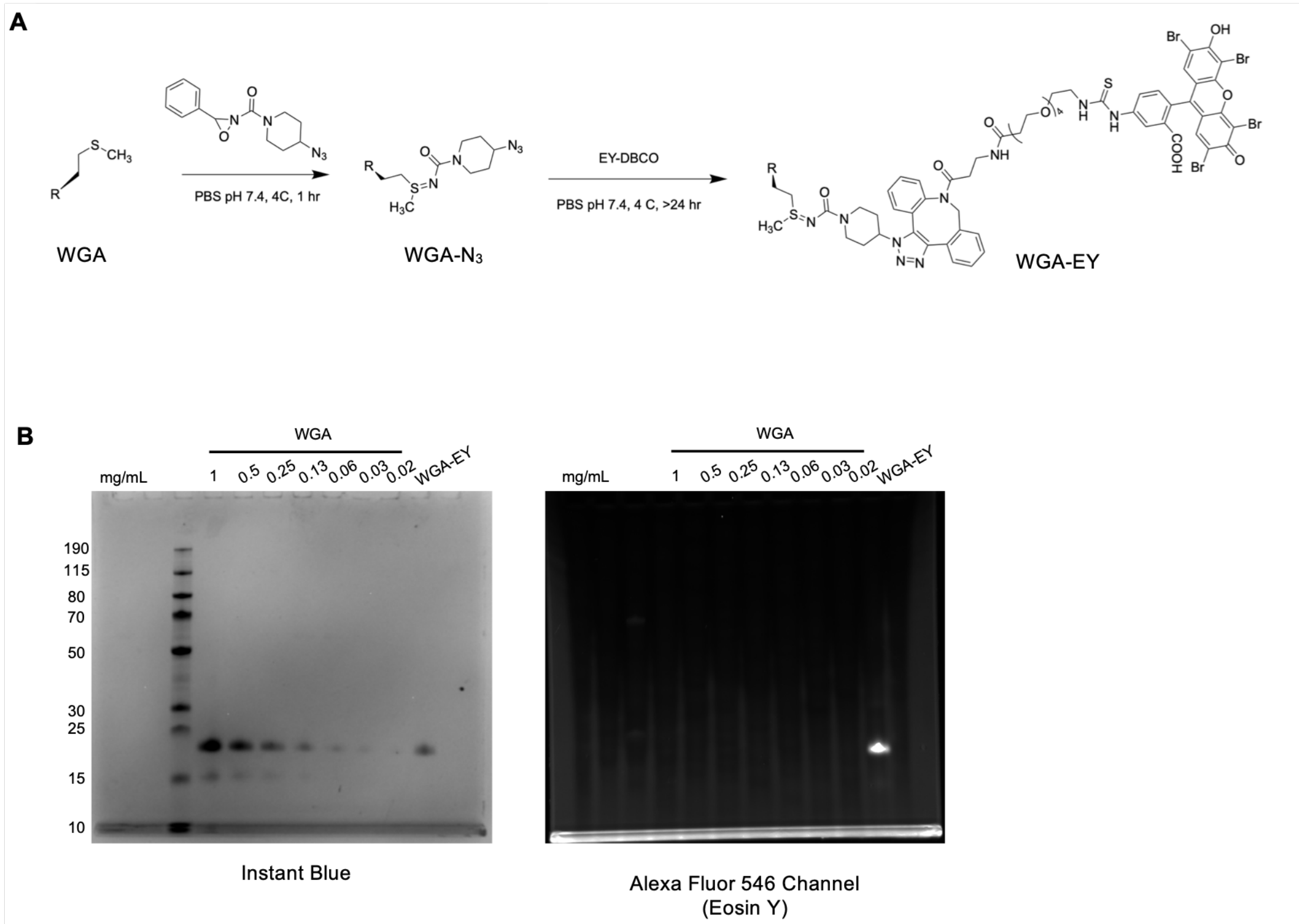

Fig. S2. Preparation of WGA-EY. (**A**) Conjugation scheme for attaching EY to WGA. (**B**) Confirmation of successful conjugation of EY onto WGA. Views of the same gel stained with Instant Blue, left, and imaged for EY fluorescence, right, are shown.

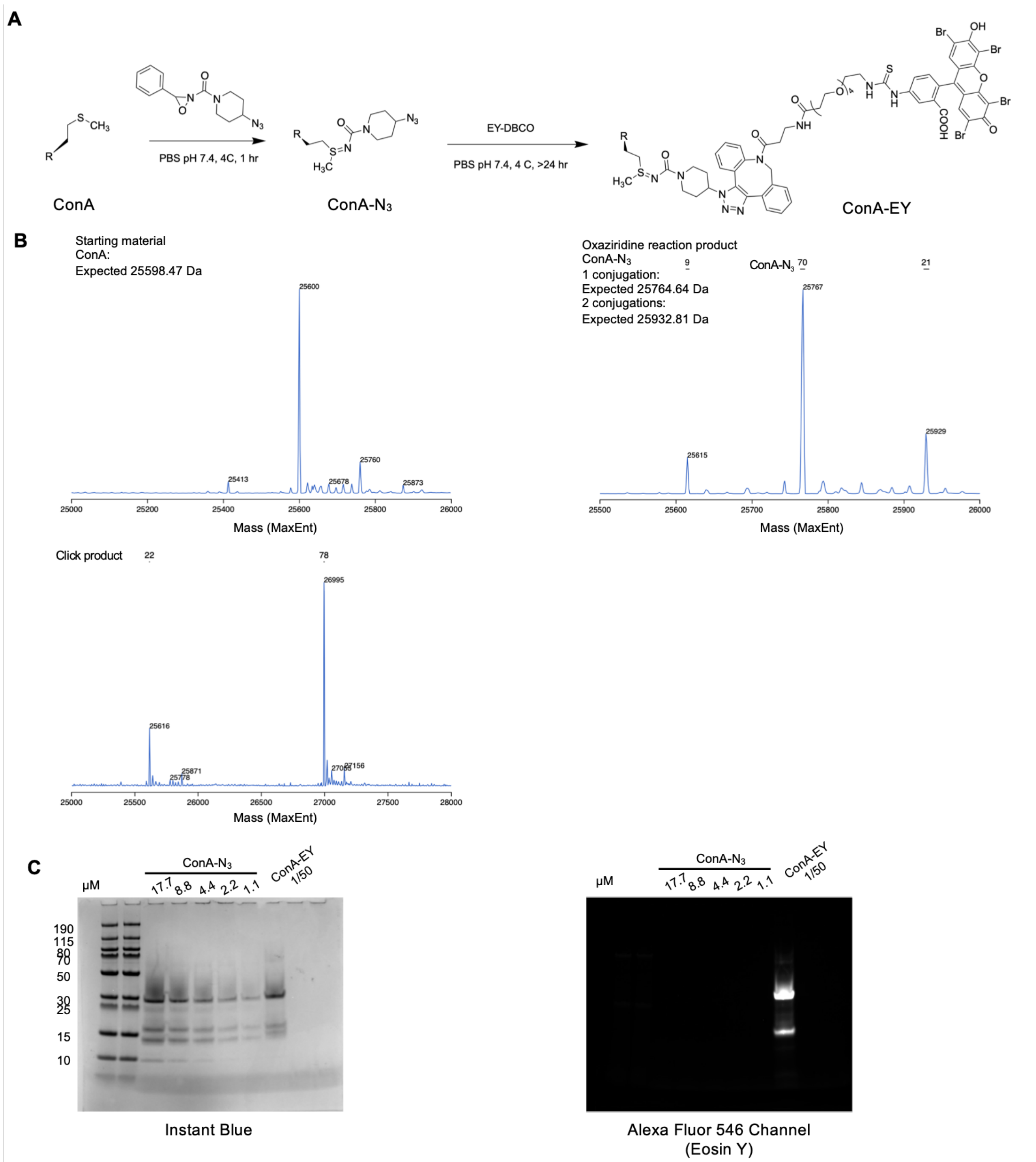

Fig. S3. Preparation of ConA-EY. (**A**) Conjugation scheme for attaching EY to ConA. Note that each ConA monomer has two methionine residues. (**B**) Mass spectrometry traces for ConA conjugation intermediates. Top left, starting material. Top right, oxaziridine conjugate. Note that the majority of ConA has a single oxaziridine-azide added, with a minor population possessing two conjugation handles. Little starting material remains, with some experiencing a single oxidation event without conjugation that presumably indicates methionine oxidation. (**C**) Confirmation of successful conjugation of EY onto ConA. Views of the same gel stained with Instant Blue, left, and imaged for EY fluorescence, right, are shown.

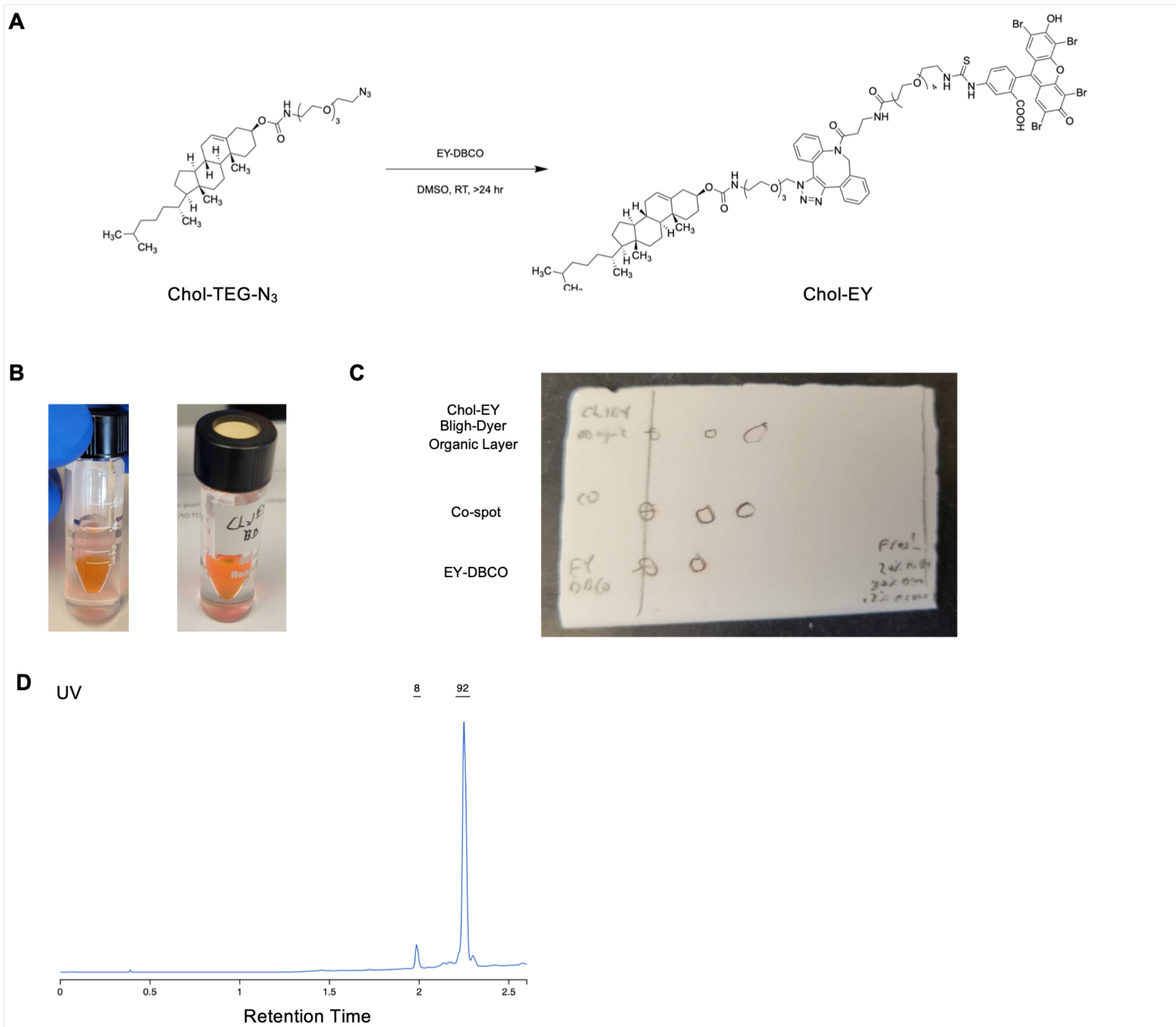

Fig. S4. Preparation of Chol-EY. (**A**) Synthetic scheme for accessing Chol-EY. (**B**) Photographs of Bligh-Dyer separation of click reaction crude. Partitioning of an orange molecule into the organic layer was seen. The aqueous layer was removed. (**C**) Thin layer chromatography of the Bligh-Dyer organic layer compared to the EY-DBCO starting material, developed in 20% MeOH in DCM. All visible spots were circled in pencil. A new spot was identified in the product. (**D**) UV trace of the product following NPLC purification of the Bligh-Dyer organic layer, injected on Xevo. The expected target mass of 1859 Da, the sum of the observed masses of the starting materials cholesterol-TEG-N_3 and EY-DBCO, was seen in the major peak.

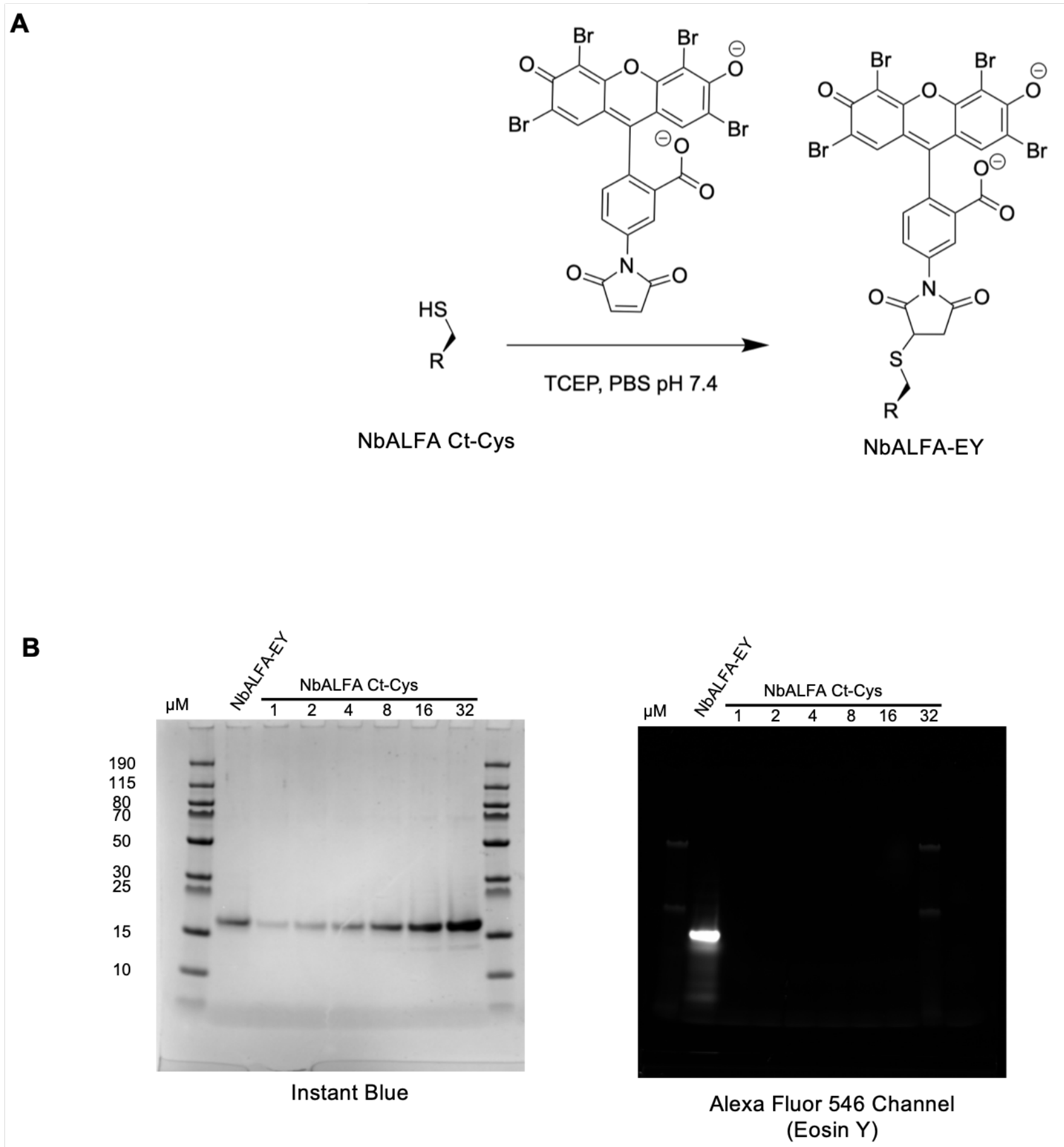

Fig. S5. Preparation of NbALFA-EY. (**A**) Conjugation scheme for attaching EY to NbALFA. (**B**) Confirmation of successful conjugation of EY onto NbALFA. Views of the same gel stained with Instant Blue, left, and imaged for EY fluorescence, right, are shown.

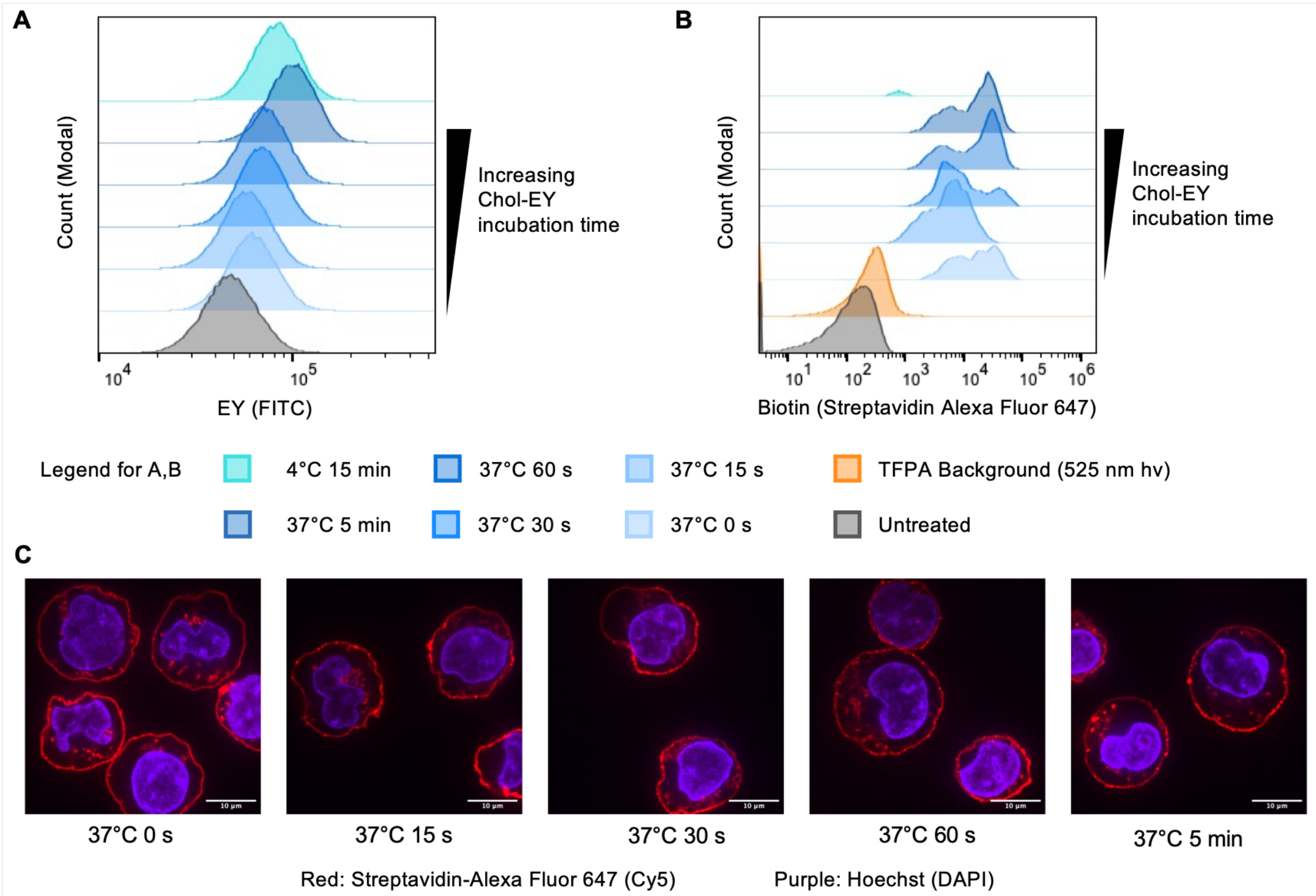

Fig. S6. Chol-EY incorporation into cells is improved at biologic temperature. (**A**) Flow cytometry histograms of nonpermeabilized cells treated with 10 μM Chol-EY either at 37°C for specified times or 4°C for 15 minutes shows greater incorporation at 37°C within 5 minutes than extended 4°C incubation. The fluorescent signal of EY is detected in the FITC channel. (**B**) After incubation with Chol-EY at 37°C for the specified time, cells were immediately placed on ice and cooled to 4°C. The photocatalytic reaction was subsequently performed at 4°C with TFPA-biotin substrate. Flow cytometry on unfixed nonpermeabilized cells, gated for live cells using Ghost Dye Violet 450 and stained with Streptavidin Alexa Fluor 647, shows superior biotinylation by the 37°C 5 min incubation condition. These data support the use of 37°C 5 min incubation for Chol-EY incorporation. (**C**) Confocal microscopy staining for biotin shows that intracellular biotin signal is not absent at lower time points than five minutes. Brightness and contrast were adjusted in each channel per image independently to highlight distribution. Scale bar 10 μm.

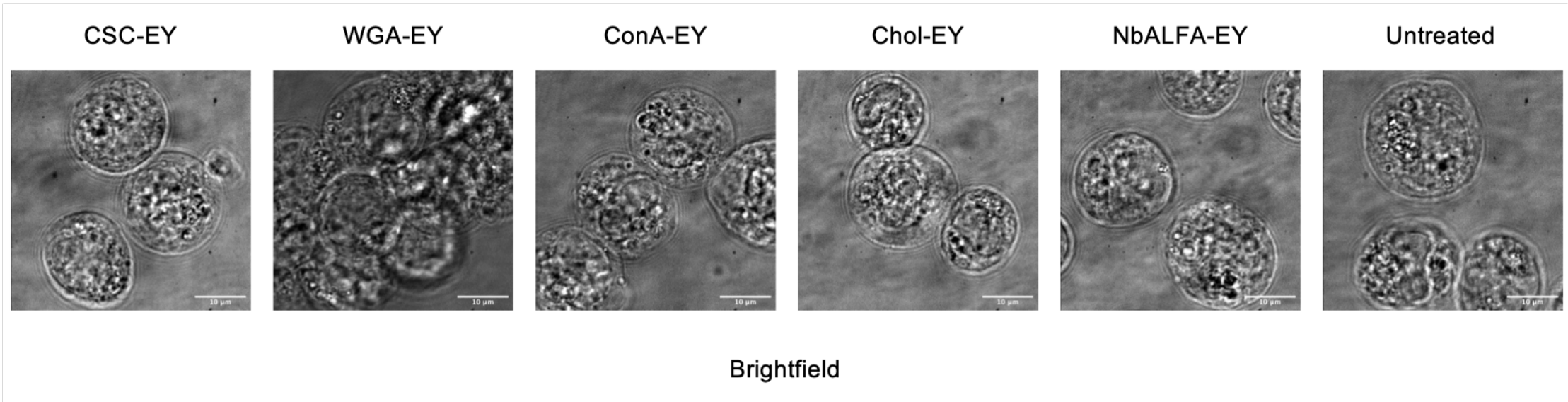

Fig. S7. EY channel intracellular puncta align with fixation blebs. Brightfield images of the fields of view shown in **Fig. 1B**. Brightness and contrast were adjusted per image independently. Scale bar 10 µm.

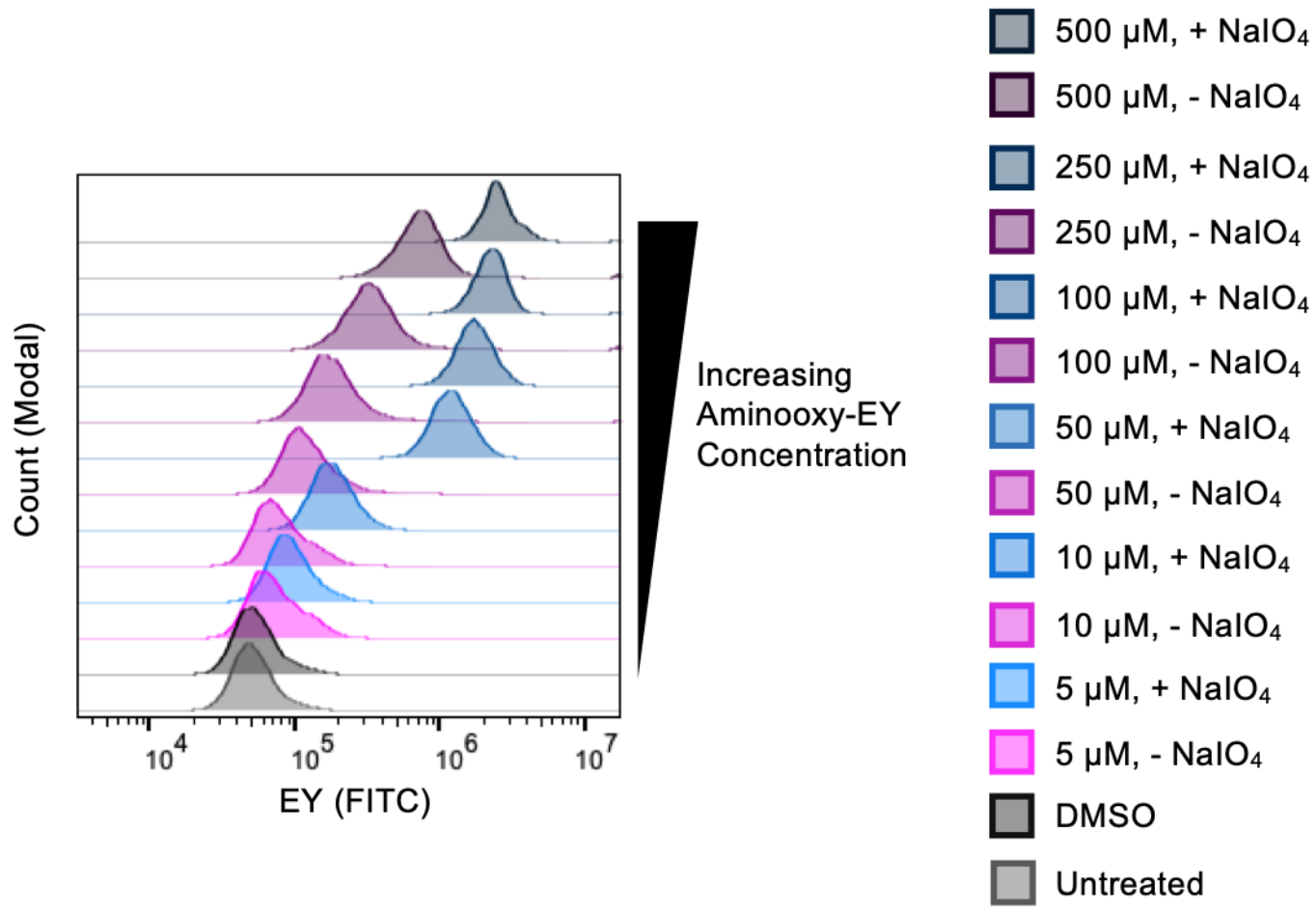

Fig. S8. Aminooxy-EY for CSC-EY binds more robustly to the cell surface after periodate oxidation. Flow cytometry on unfixed nonpermeabilized cells, gated for live cells using Ghost Dye Violet 450, treated with varying concentrations of aminooxy-EY with 1:1000 v/v aniline following periodate oxidation or mock treatment. The largest distinction between nonspecific binding and glycan tethering was observed at 50 µM, and thus CSC-EY experiments were performed with 50 µM aminooxy-EY. The fluorescence of EY was detected via the FITC channel.

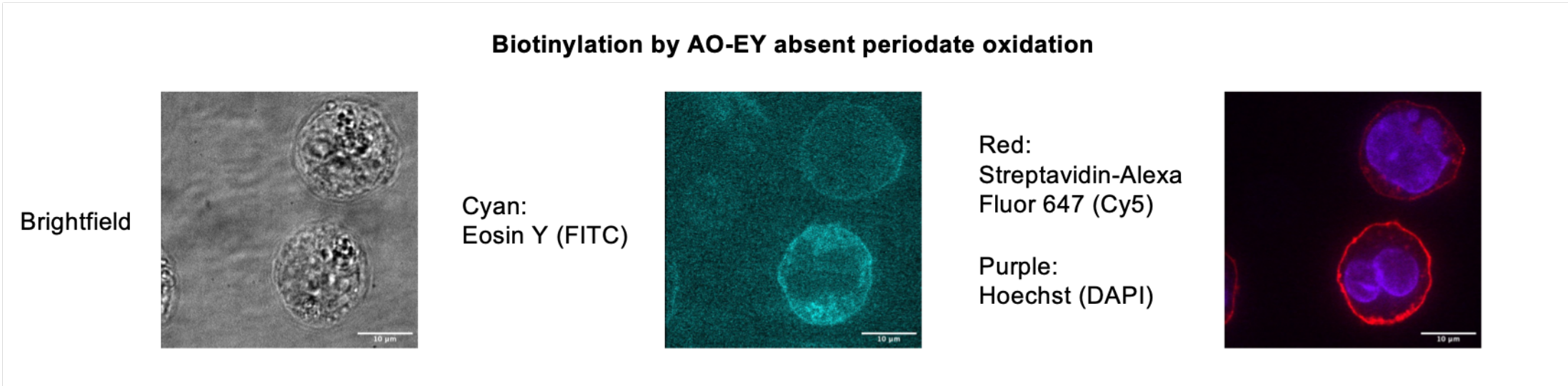

Fig. S9. Aminooxy-EY binding absent periodate oxidation is also cell surface constrained. Confocal microscopy of cells treated with aminooxy-EY at 50 μM in the absence of periodate oxidation shows mild attachment to cells that is predominantly cell surface. The cells were noted to have mild confounding autofluorescence in the FITC channel that could contribute to the appearance of off-target intracellular signal. This residual aminooxy-EY contributes additional biotinylation signal. Brightness and contrast were adjusted in each channel per image independently to highlight distribution. Scale bar 10 μm. Note that these images were taken after the light-induced biotinylation that may induce EY photobleaching.

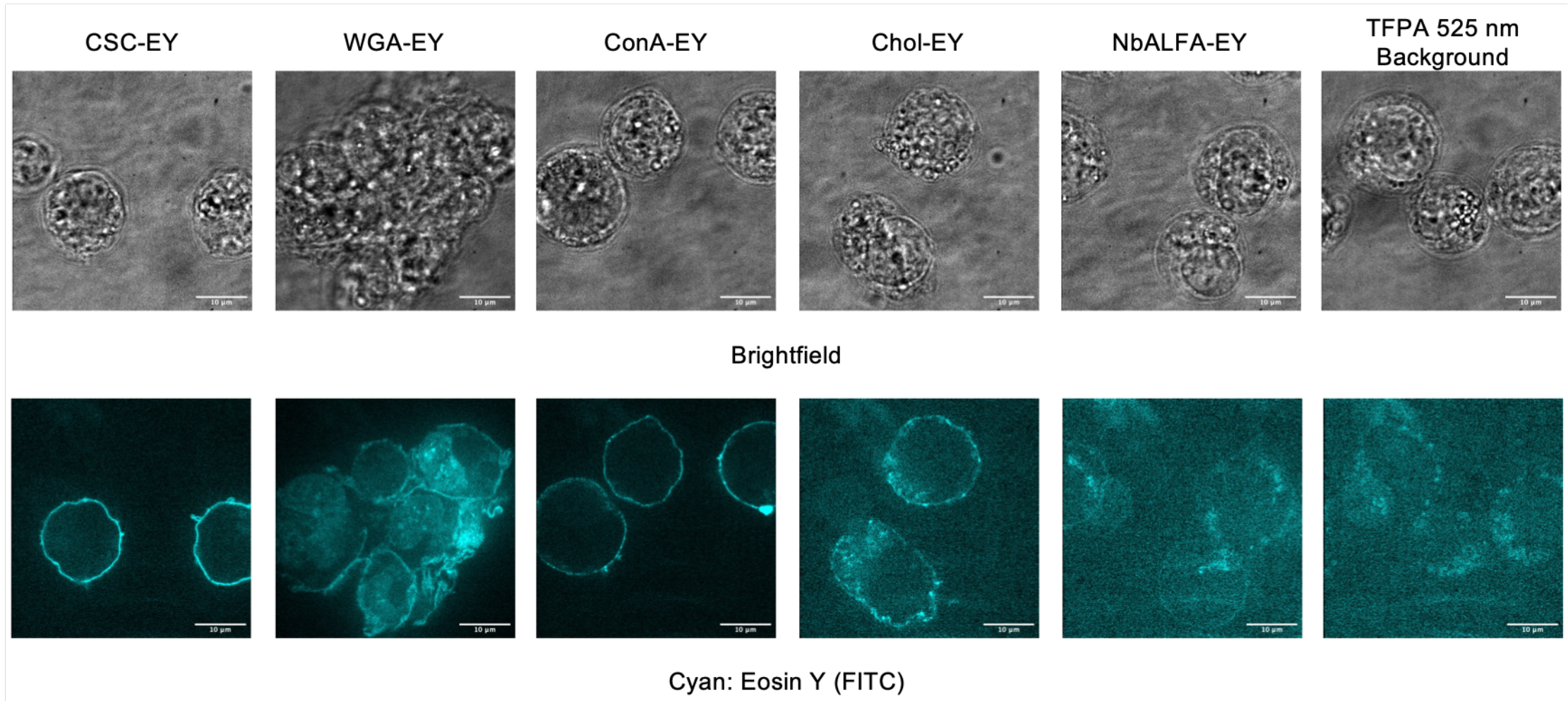

Fig. S10. Residual EY fluorescence aligns with biotinylation signal. Brightfield and EY channel views of the same cells in **Fig. 1C**. Although these images were taken after irradiation that may photobleach EY, some EY signal remained and aligns with the biotin signal. Brightness and contrast were adjusted in each channel per image independently to highlight distribution. Scale bar 10 μm.

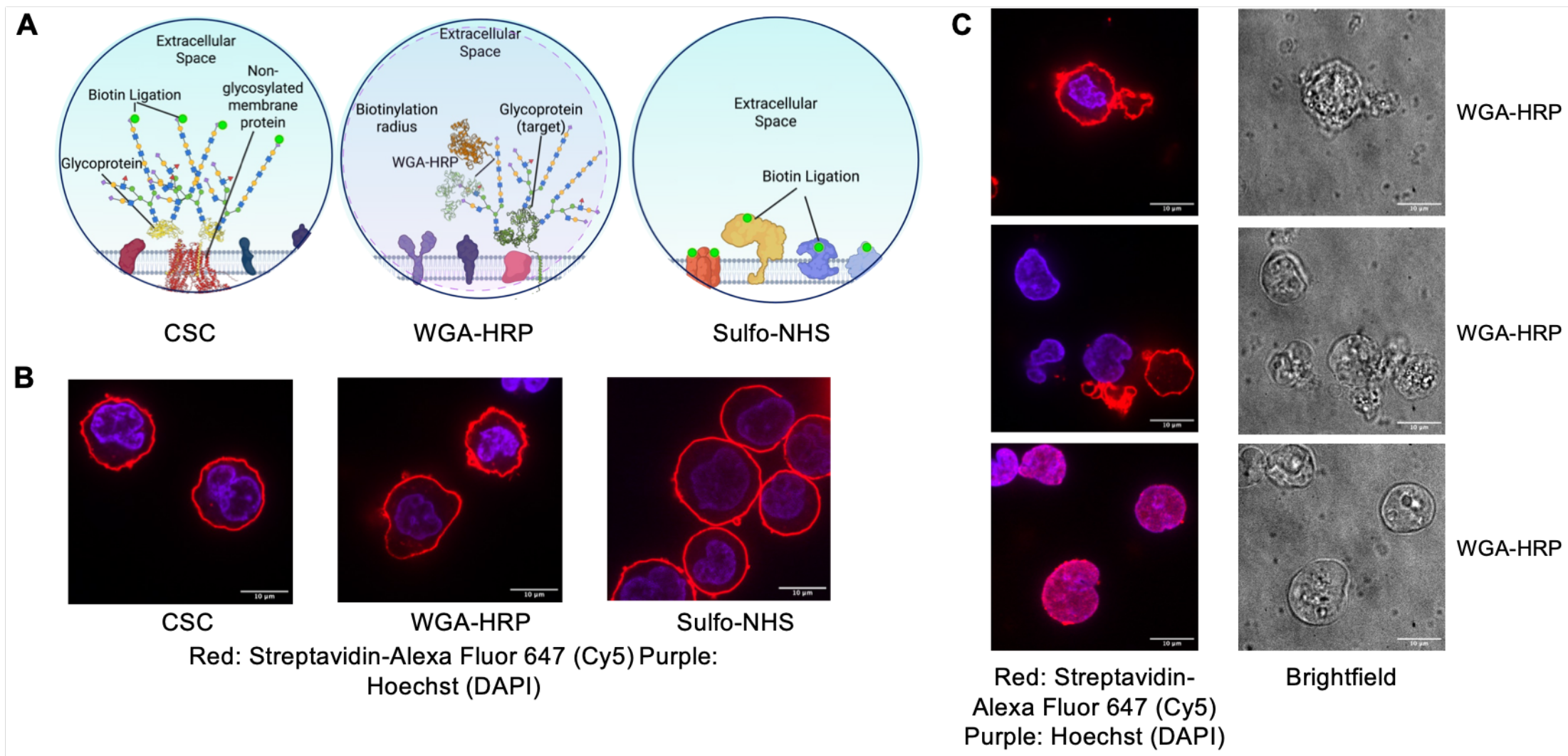

Fig. S11. Established non-photocatalytic surfaceomics methods label the cell surface as expected. (**A**) Schematic of literature control biotinylation strategies. CSC directly biotinylates glycans, WGA-HRP binds glycans and generates biotin-phenolic radicals that primarily insert into tyrosine on proteins, and sulfo-NHS directly biotinylates lysines on proteins. Figure created with Biorender.com. Structure of WGA: PDB 7WGA (33). Membrane protein structures were generated in AlphaFold 3 (35) and rendered in ChimeraX (36). Illustration is not to scale and purely for visualization. (**B**) Confocal microscopy staining for biotin shows that CSC, WGA-HRP, and sulfo-NHS are cell surface selective. Brightness and contrast were adjusted in each channel per image independently to highlight distribution. Scale bar 10 µm. (**C**) Different fields of view from the same WGA-HRP sample reveal that WGA-HRP labeling is heterogeneous, with some cells not labeled and anucleate ghost cells labeled. Brightfield for WGA-HRP also displays irregular phenotype with ample debris. Brightness and contrast were adjusted in each channel per image independently to highlight distribution. Scale bar 10 µm.

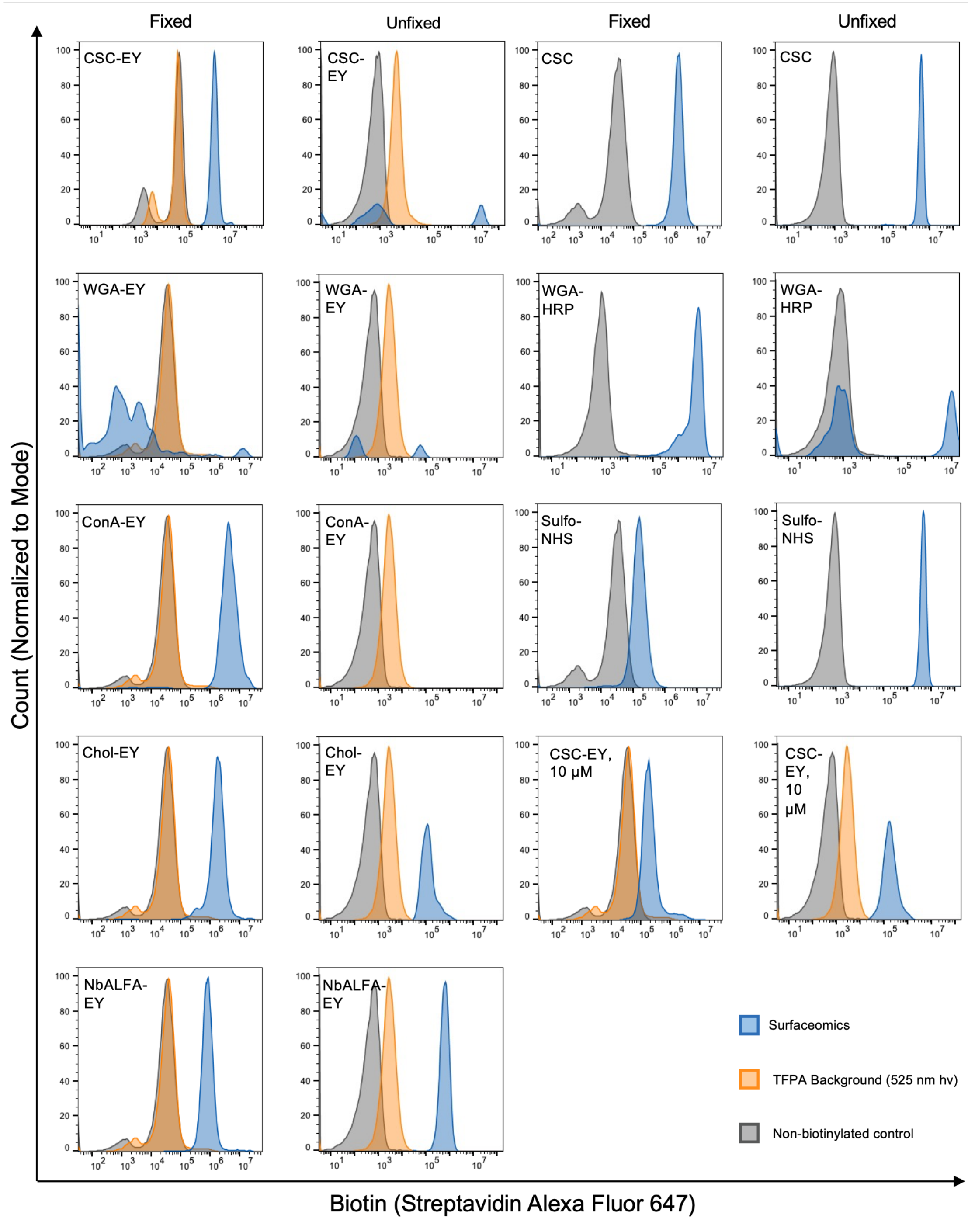

Fig. S12. Flow cytometry shows substantial biotinylation by all surfaceomics techniques. Biotin detection on non-permeabilized cells was performed with and without fixation using paraformaldehyde. Streptavidin-Alexa Fluor 647 distributions normalized to mode are shown after singlet gating. For unfixed cells, a Ghost Dye Violet 450-based gating for live cells was also used. For many photo-PLP and enzymatic strategies, fixation improved cell recovery for flow cytometry. The fixed samples depicted here were used for quantification shown in **Fig. 1E**.

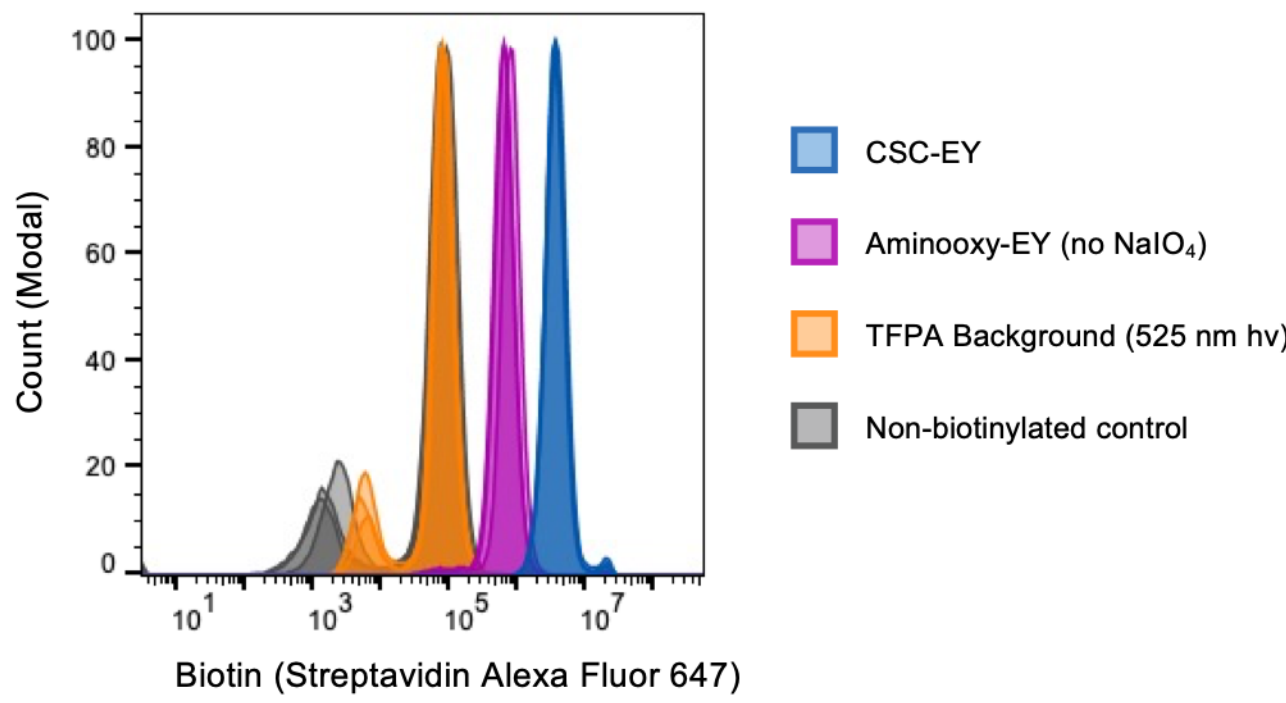

Fig. S13. CSC-EY achieves greater cell surface biotinylation than aminooxy-EY in the absence of periodate oxidation. Flow cytometry of non-permeabilized fixed cells staining for biotin after cell surface biotinylation by CSC-EY, aminooxy-EY without periodate oxidation, or TFPA-biotin probe with green light in the absence of EY. Histograms of biologic triplicate are shown.

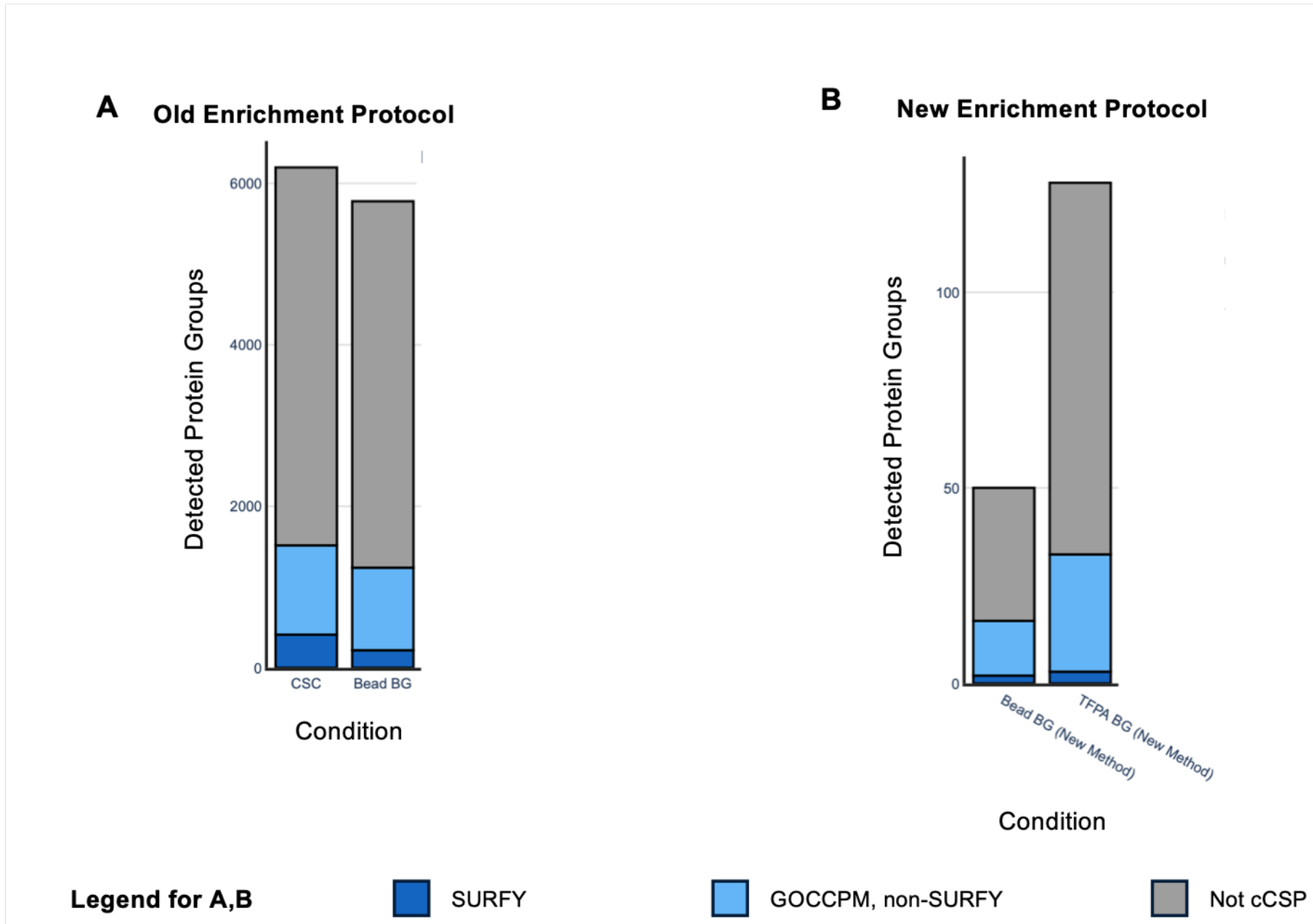

Fig. S14. Improved enrichment protocol substantially reduces nonspecific protein detection. (**A**) K562 cells were biotinylated by CSC or simply lysed, then processed for biotinylated protein enrichment per previously reported enrichment protocols. DIA-PASEF data were acquired and quantitation performed using directDIA in Spectronaut. Protein groups that appeared in at least two biologic replicates for each condition are shown. Note the substantial background in the Bead BG sample. (**B**) Optimized protein enrichment protocol used in this study substantially reduces background binding of non-biotinylated proteins. Data reflect DIA-PASEF data searched against pooled high pH fractionated library. Protein groups that appeared in at least two biologic replicates for each condition are shown. Note the difference in y axis scale between this plot and the plot in A.

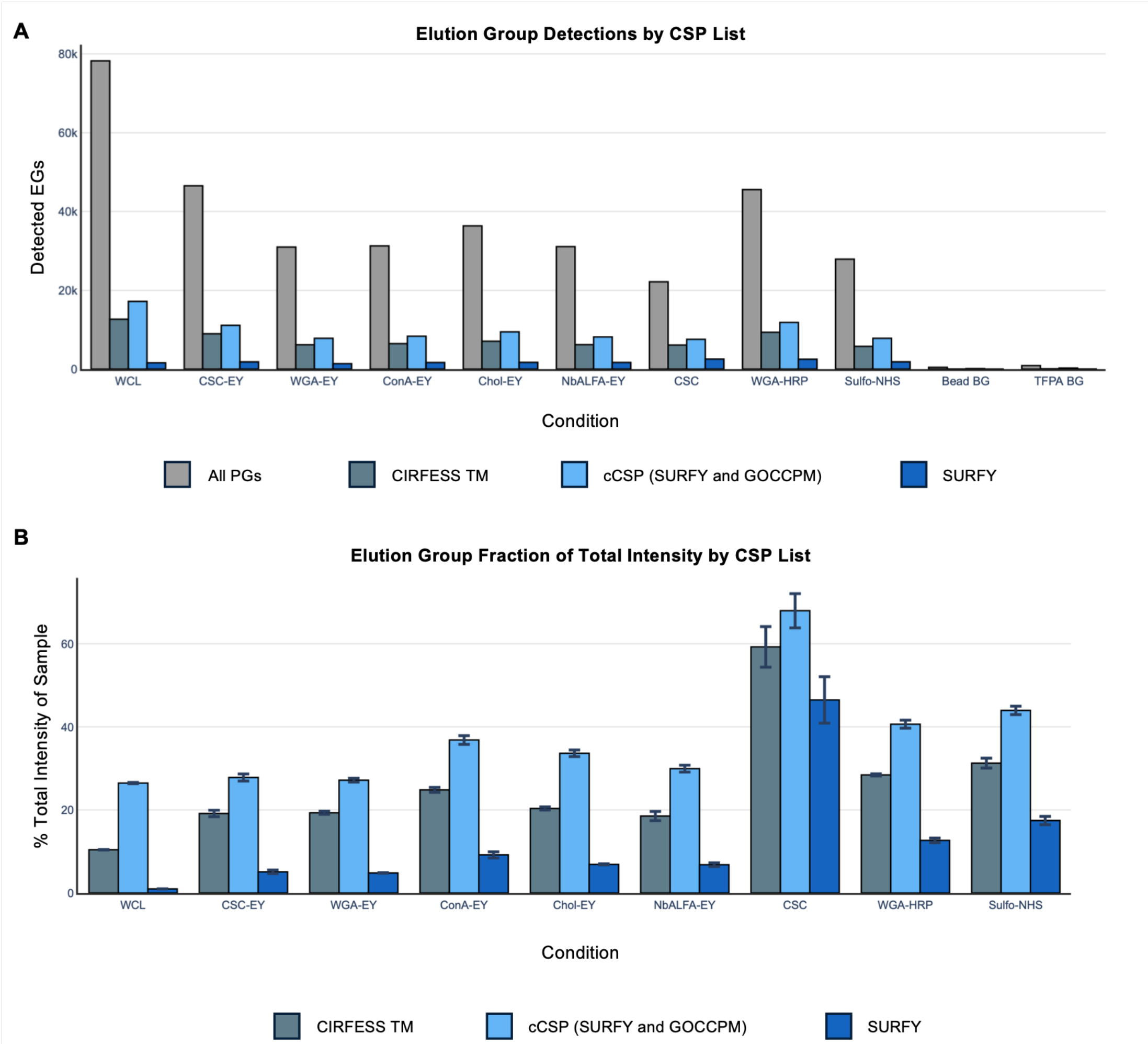

Fig. S15. Elution group level analysis shows cCSP enrichment by all surfaceomics methods. (**A**) The number of EGs that were detected in at least two biologic replicates of each condition is shown. Note substantial reduction of all EGs in surfaceomics compared to unenriched whole cell lysate, as well as cleanliness of negative controls Bead BG and TFPA BG. (**B**) Fractional LFQ intensity at the EG level contributed by proteins from cCSP lists and CIRFESS TM. Pre-imputation total intensity quantities at the EG level were summed for all EGs attributed to PGs belonging to specified CSP lists, then divided by the summed intensity of all EGs detected with a sample. Data are mean of biological triplicate with standard deviation error bars.

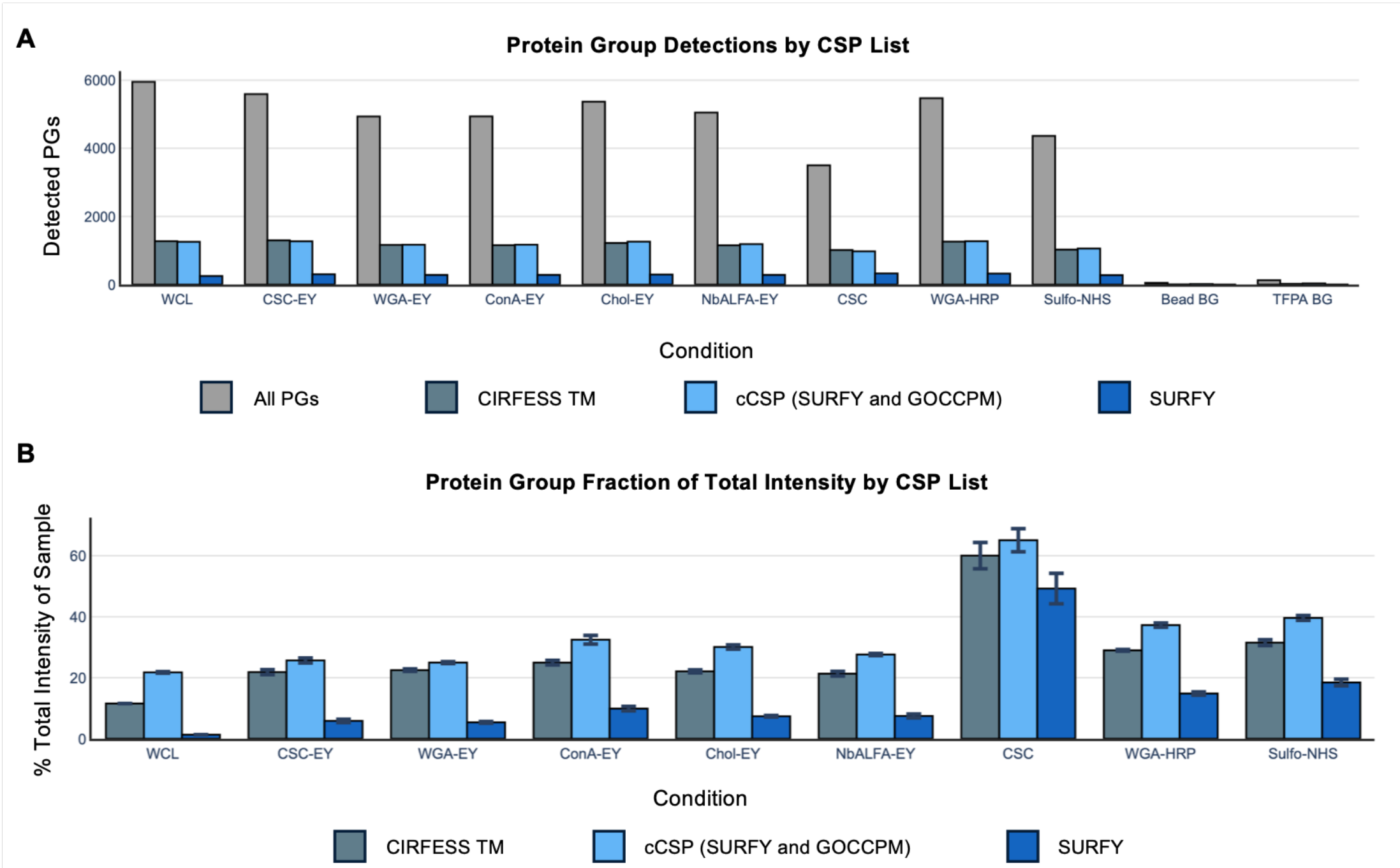

Fig. S16. Protein group level analyses with both cCSP lists and CIRFESS TM reflect increases in CSP detection by surfaceomics (**A**) The number of PGs that were detected in at least two biologic replicates of each condition is shown. Note the cleanliness of negative controls Bead BG and TFPA BG as well as the relative parity of cCSP detections across all surfaceomics methods and WCL. (**B**) Fractional LFQ intensity at the PG level contributed by proteins from cCSP lists and CIRFESS TM. Pre-imputation total intensity quantities at the PG level were summed for all PGs belonging to specified CSP lists then divided by the summed intensity of all PGs detected with a sample. Note that WCL shows a smaller increase from SURFY to CIRFESS TM, relative to the increase from SURFY to all cCSPs, than surfaceomics methods do. Data are mean of biological triplicate with standard deviation error bars.

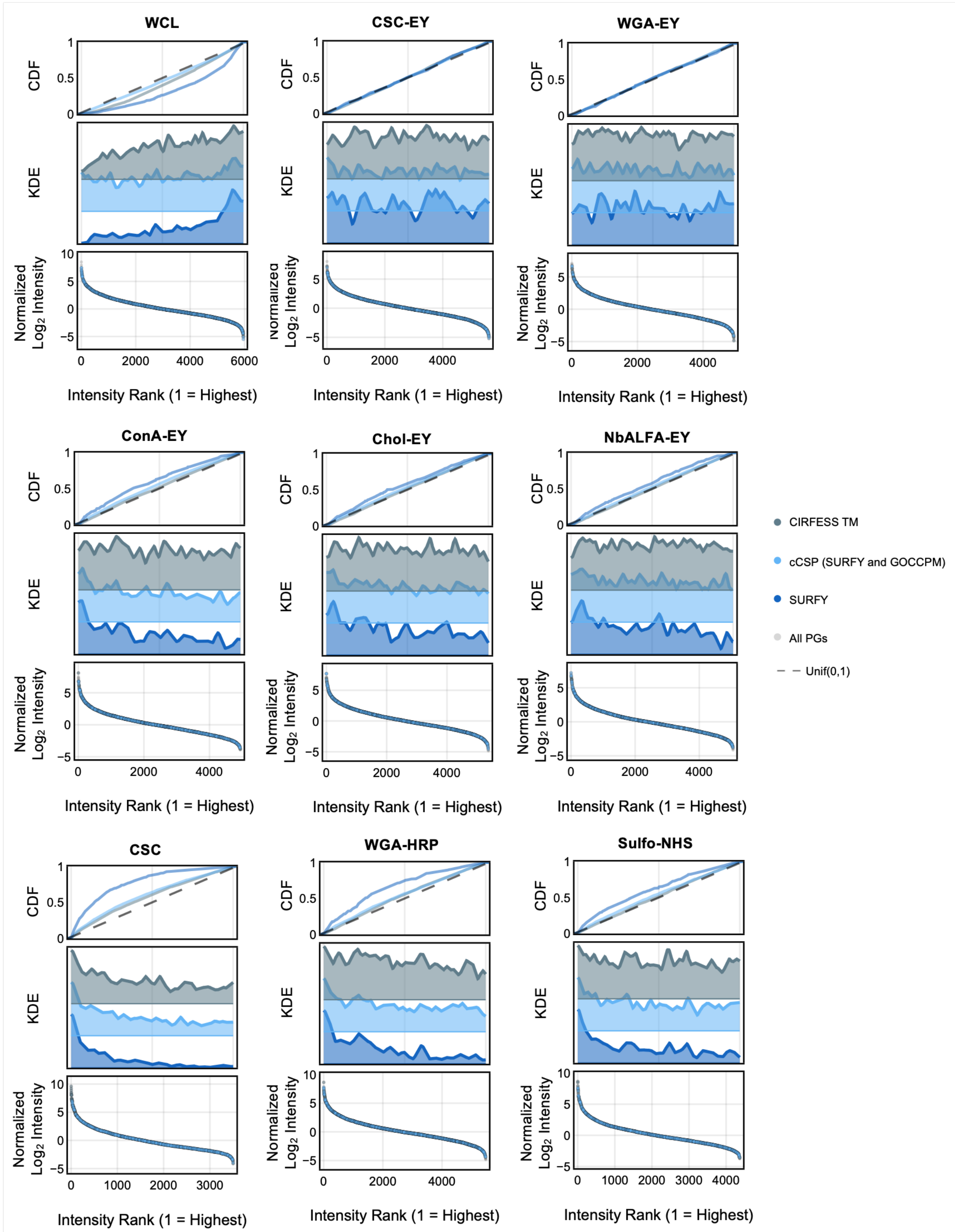

Fig. S17. cCSPs are more highly quantified by all surfaceomics methods compared to unenriched whole cell lysate (WCL). Top panel: The empirical cumulative distribution function (CDF) of cCSP and CIRFESS TM PGs was quantified over intensity rank (solid lines) and compared to a null hypothesis of uniform distribution across ranks (dashed diagonal line). **Center panel:** Corresponding kernel density estimates (KDE) were calculated (filled curves). All surfaceomics methods showed far greater left shift of KDE than the unenriched WCL. **Bottom panel:** Waterfall plots are shown, demonstrating distribution of protein group intensities across

rank. Highlighted are cCSPs. Intensity measurements reflect the mean of biologic triplicate, normalized to the median intensity. Missing values were imputed by MNAR algorithm for averaging of detected proteins, but only PGs that were detected in at least two biologic replicates of a condition are considered for these plots.

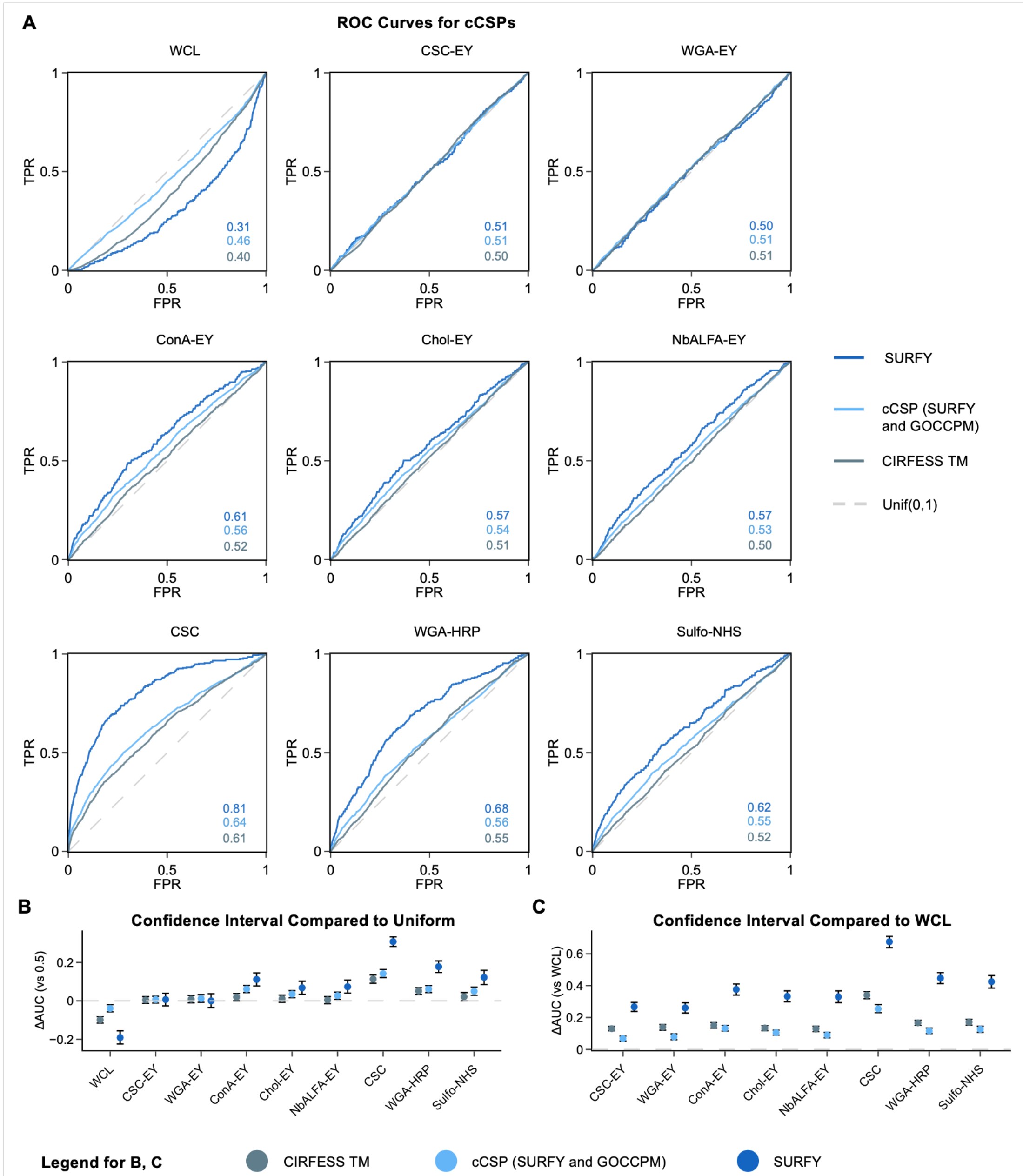

Fig. S18. Receiver operating characteristic curves confirm superior recovery of cCSPs by surfaceomics compared to unenriched whole cell proteomics. (**A**) ROC curves for each surfaceomics method and WCL were plotted, considering SURFY, cCSPs, or CIRFESS TM to be true positives. The null hypothesis (uniform) is shown as a dashed line. The AUC of each curve is listed on the bottom right of the plot in the color corresponding to which list was considered positive (top: SURFY, middle: cCSP, bottom: CIRFESS TM). Intensity measurements used to order the proteins reflect the mean of biologic triplicate with imputed values. Only PGs that were detected in at least two biologic replicates of a condition are considered for these plots. TPR, true positive rate. FPR, false positive rate. (**B**) Nonparametric bootstrap 95% confidence intervals were computed on the difference between ROC AUC of each sample and uniform (0.5), supporting cCSP enrichment by all surfaceomics methods except CSC-EY and WGA-EY. (**C**) Nonparametric bootstrap 95% confidence intervals were computed on the difference between

ROC AUC of each sample and the WCL ROC AUC, supporting cCSP enrichment by all surfaceomics methods relative to whole cell proteomics.

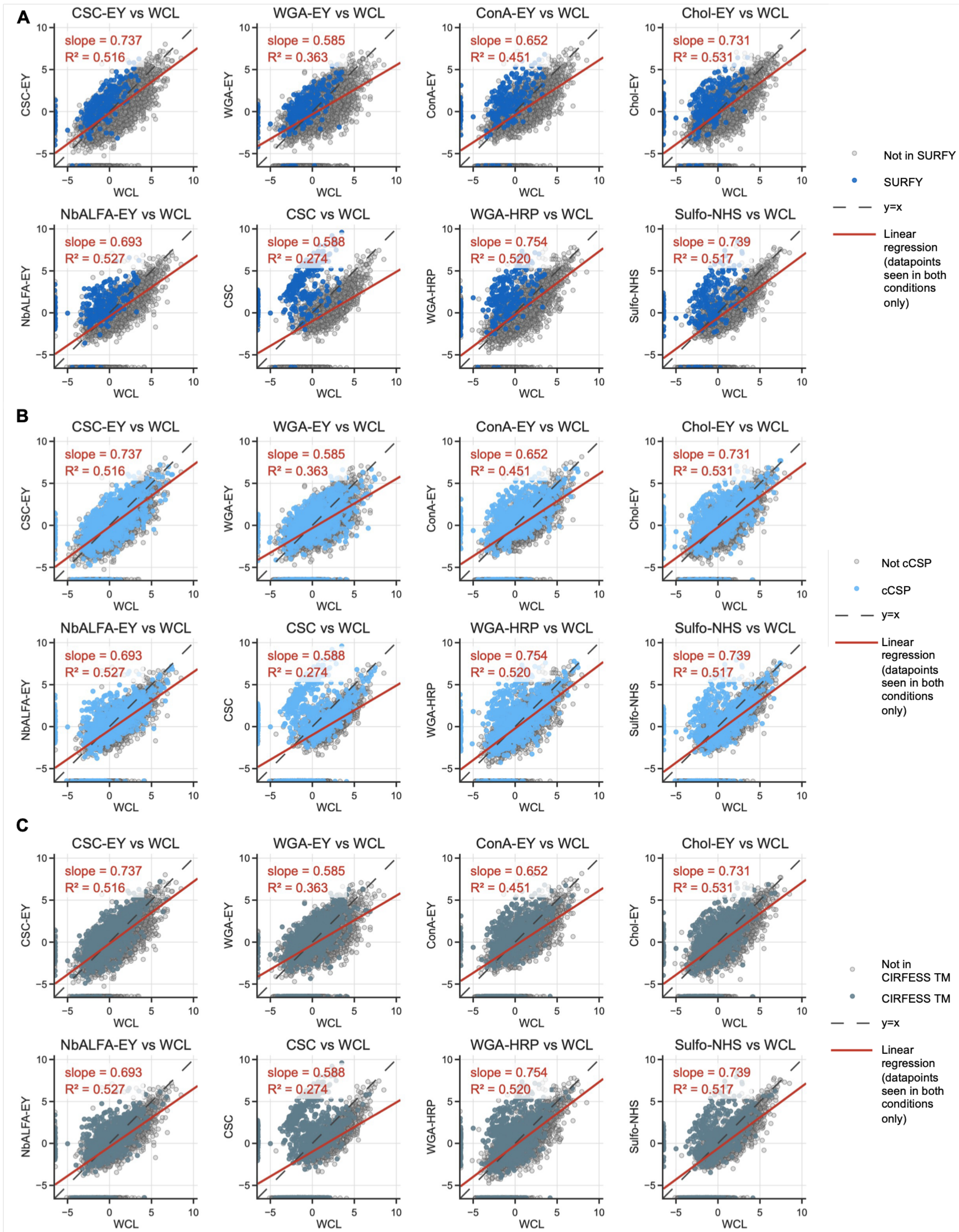

Fig. S19. Scatterplot comparison between surfaceomics methods and WCL show enhanced cCSP quantitation by surfaceomics. (**A**) Median normalized intensities of PGs detected by each surfaceomics method are plotted against their intensities in WCL. PGs that were detected in the surfaceomics condition but not WCL are clamped to the y axis. PGs detected only in WCL but not the surfaceomics condition are clamped to the x axis. The line of equality is shown as a dashed line and the linear regression for PGs detected in both conditions is shown as a solid red line. SURFY PGs are colored blue and skew above the linear regression

line, indicating increased relative detection by surfaceomics over WCL. Intensities are the average of biological triplicate with MNAR imputed values for missing replicates. (**B**) Plot in A, but with cCSPs colored light blue. (**C**) Plot in A, but with CIRFESS TM PGs colored blue-grey.

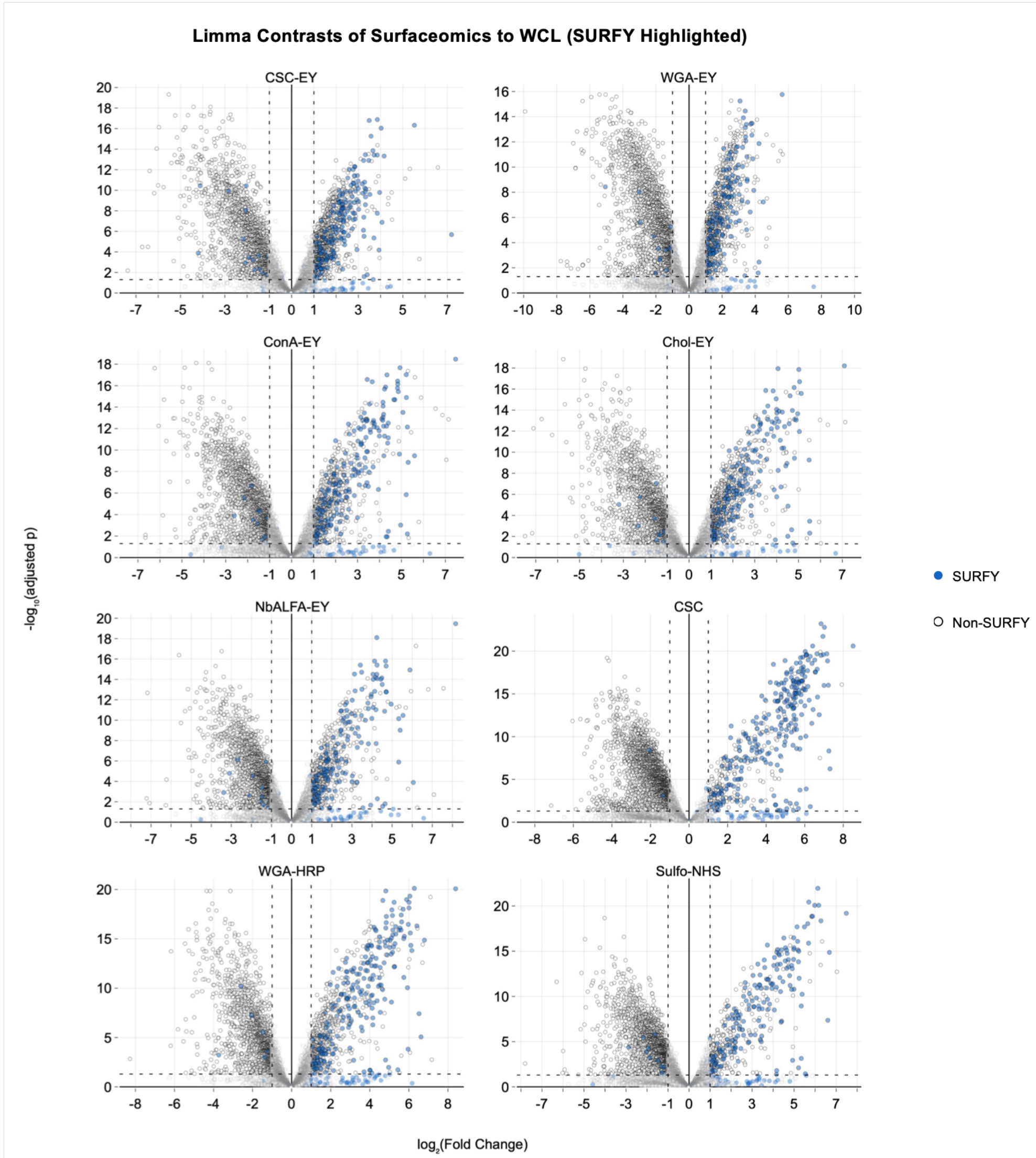

Fig. S20. Limma enrichment comparisons between surfaceomics methods and WCL highlight enrichment of SURFY PGs by surfaceomics. Limma contrasts were performed between each surfaceomics condition and WCL using median normalized intensities with MNAR imputation. On each plot, the right side corresponds to the surfaceomics method and the left to WCL. Dashed lines are shown for the enrichment thresholds, $|\log_2(\text{fold change})| \geq 1$ and adjusted $p < 0.05$. SURFY PGs are colored blue and heavily favor the surfaceomics enriched region. Note that imputed values were given reduced weights in limma, yielding suppressed adjusted p values for PGs that were fully missing in one of the compared conditions. Data reflect mean of biologic triplicate.

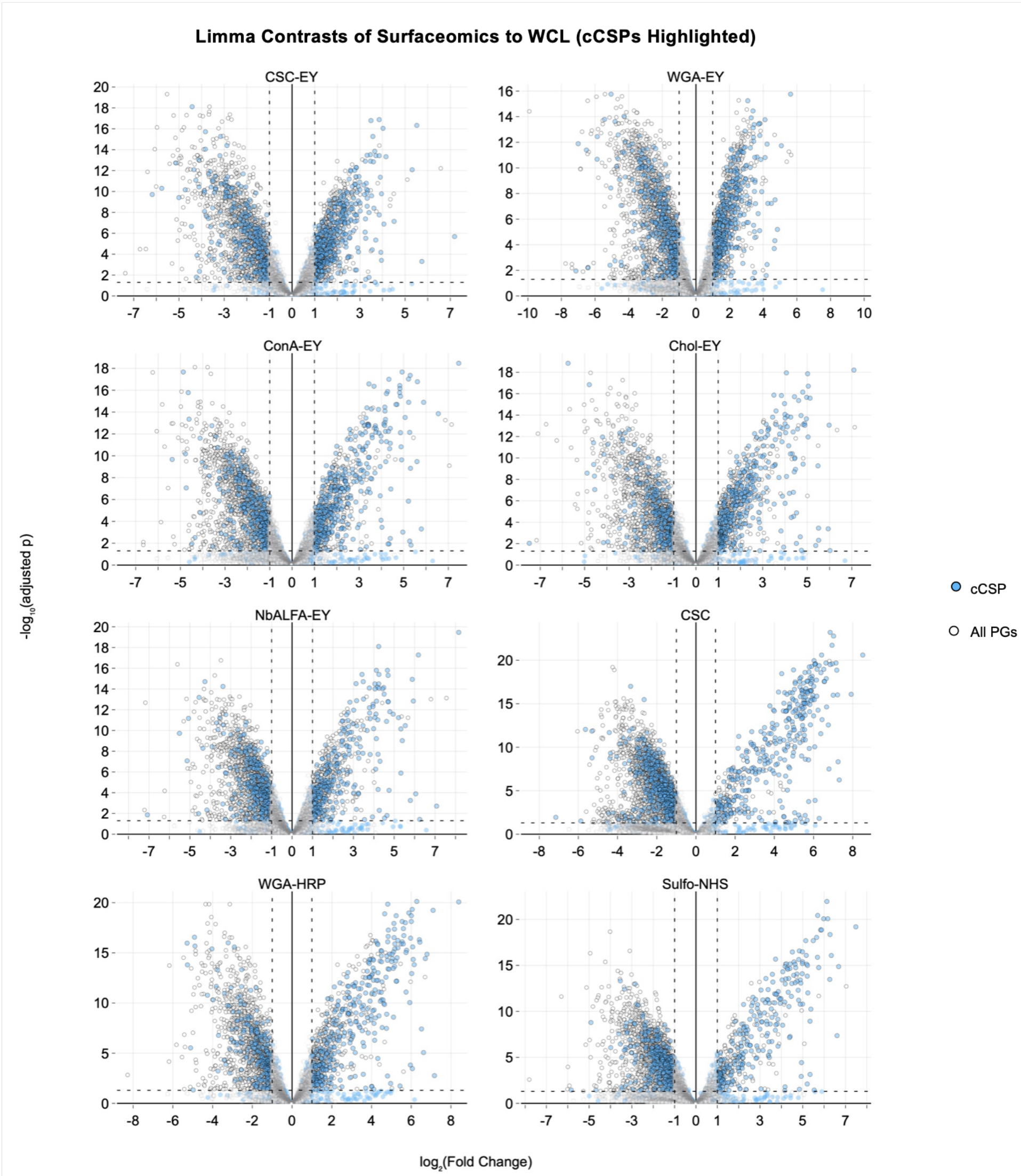

Fig. S21. Limma enrichment comparisons between surfaceomics methods and WCL with cCSP highlighted. Limma contrasts were performed between each surfaceomics condition and WCL using median normalized intensities with MNAR imputation. On each plot, the right side corresponds to the surfaceomics method and the left to WCL. Dashed lines are shown for the enrichment thresholds, $|\log_2(\text{fold change})| \geq 1$ and adjusted $p < 0.05$. All cCSP PGs are colored light blue. Note that imputed values were given reduced weights in limma, yielding suppressed adjusted p values for PGs that were fully missing in one of the compared conditions. Data reflect mean of biologic triplicate.

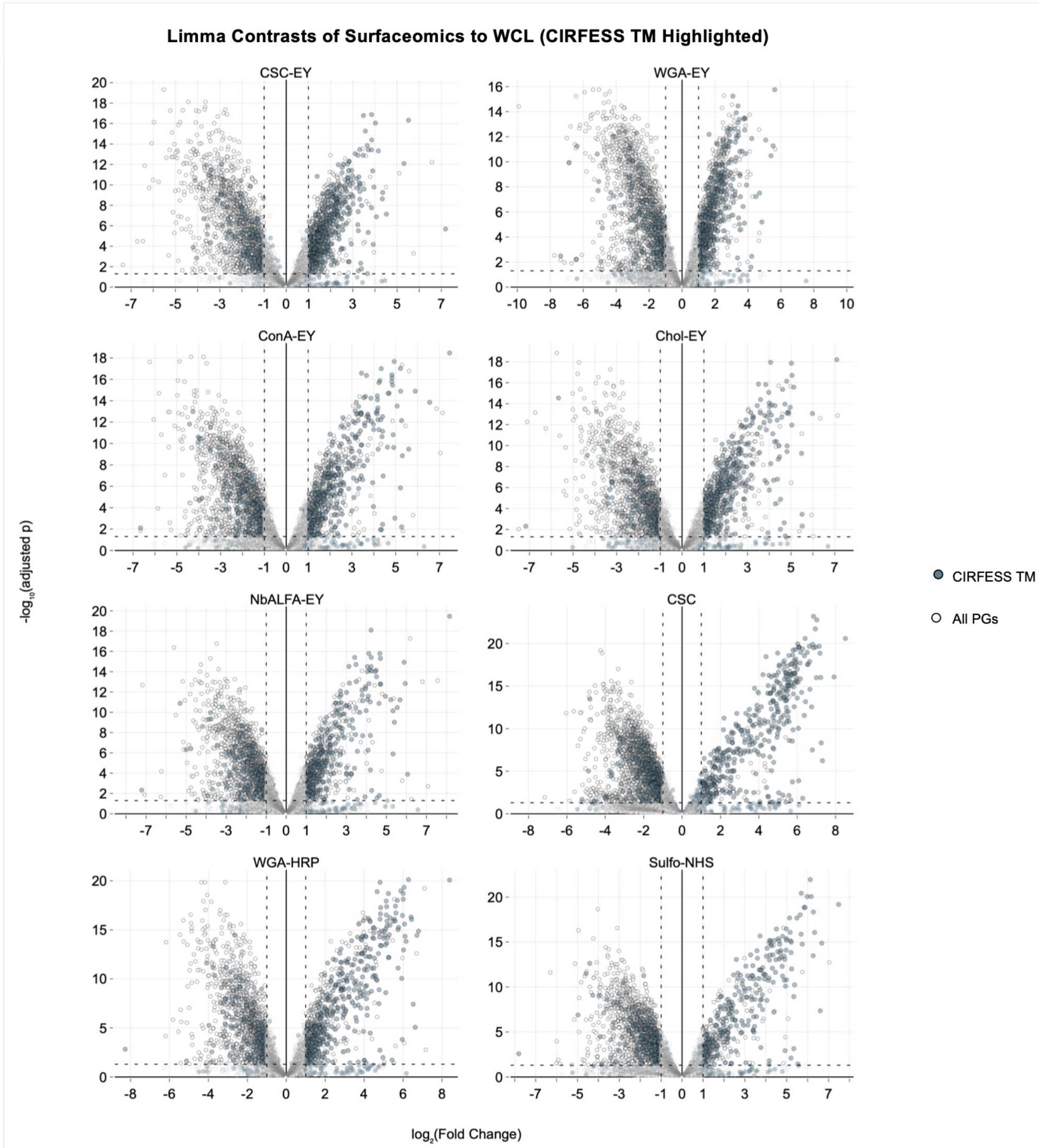

Fig. S22. Limma enrichment comparisons between surfaceomics methods and WCL with CIRFESS TM highlighting corroborates cCSP results. Limma contrasts were performed between each surfaceomics condition and WCL using median normalized intensities with MNAR imputation. On each plot, the right side corresponds to the surfaceomics method and the left to WCL. Dashed lines are shown for the enrichment thresholds, $|\log_2(\text{fold change})| \geq 1$ and adjusted $p < 0.05$. CIRFESS TM PGs are colored blue-grey. Note that imputed values were given reduced weights in limma, yielding suppressed p values for PGs that were fully missing in one of the compared conditions. Data reflect mean of biologic triplicate.

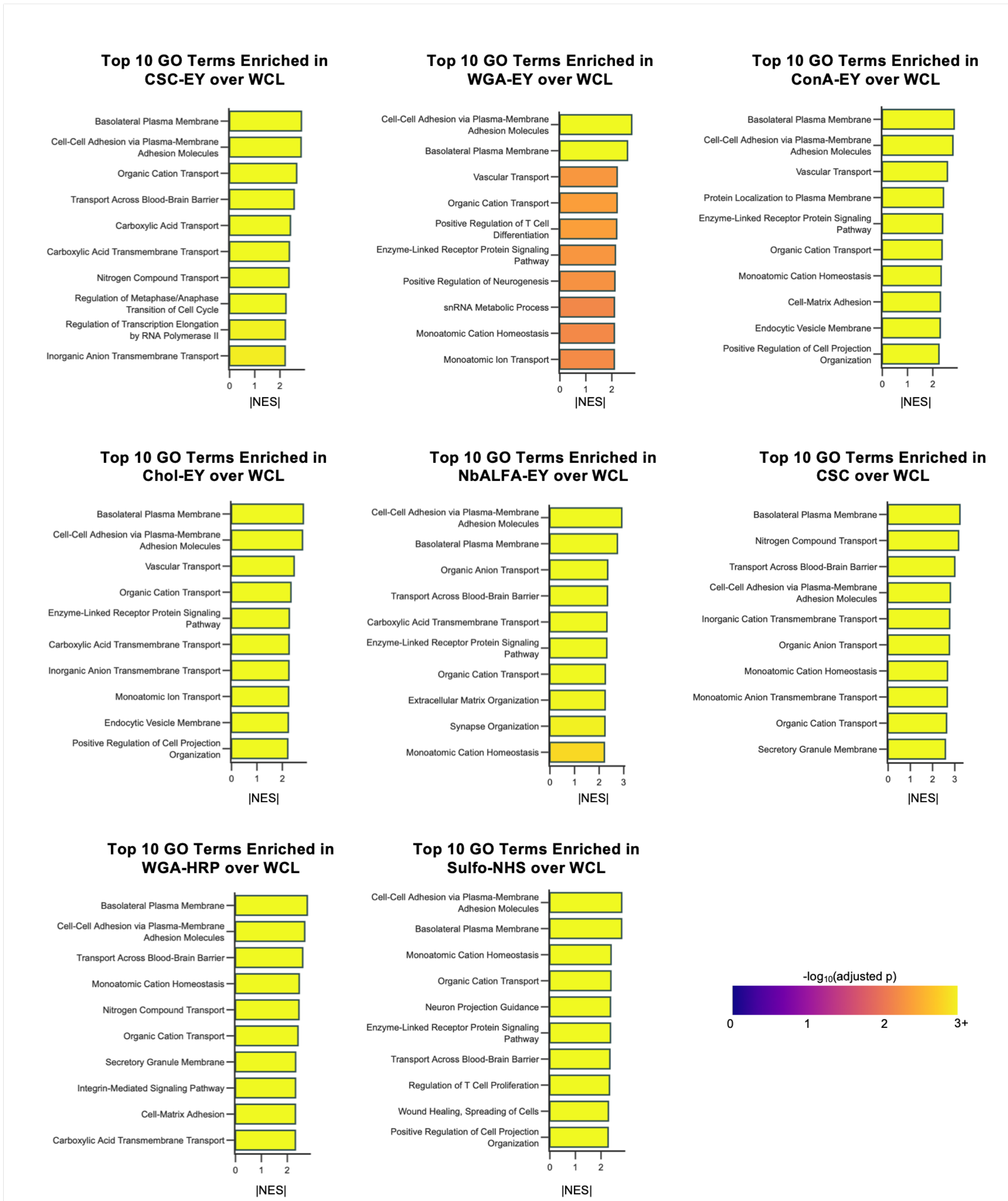

Fig. S23. Gene set enrichment analysis affirms PM enrichment for all surfaceomics methods. Median normalized intensities for all surfaceomics methods were compared pairwise to WCL for GSEA. The top 10 terms enriched by the surfaceomics methods with FDR < 0.05 and ordered by normalized enrichment score (NES) magnitude greater than 1.5 are shown. Note the presence of PM or PM-relevant annotations for every surfaceomics method with FDR < 0.001.

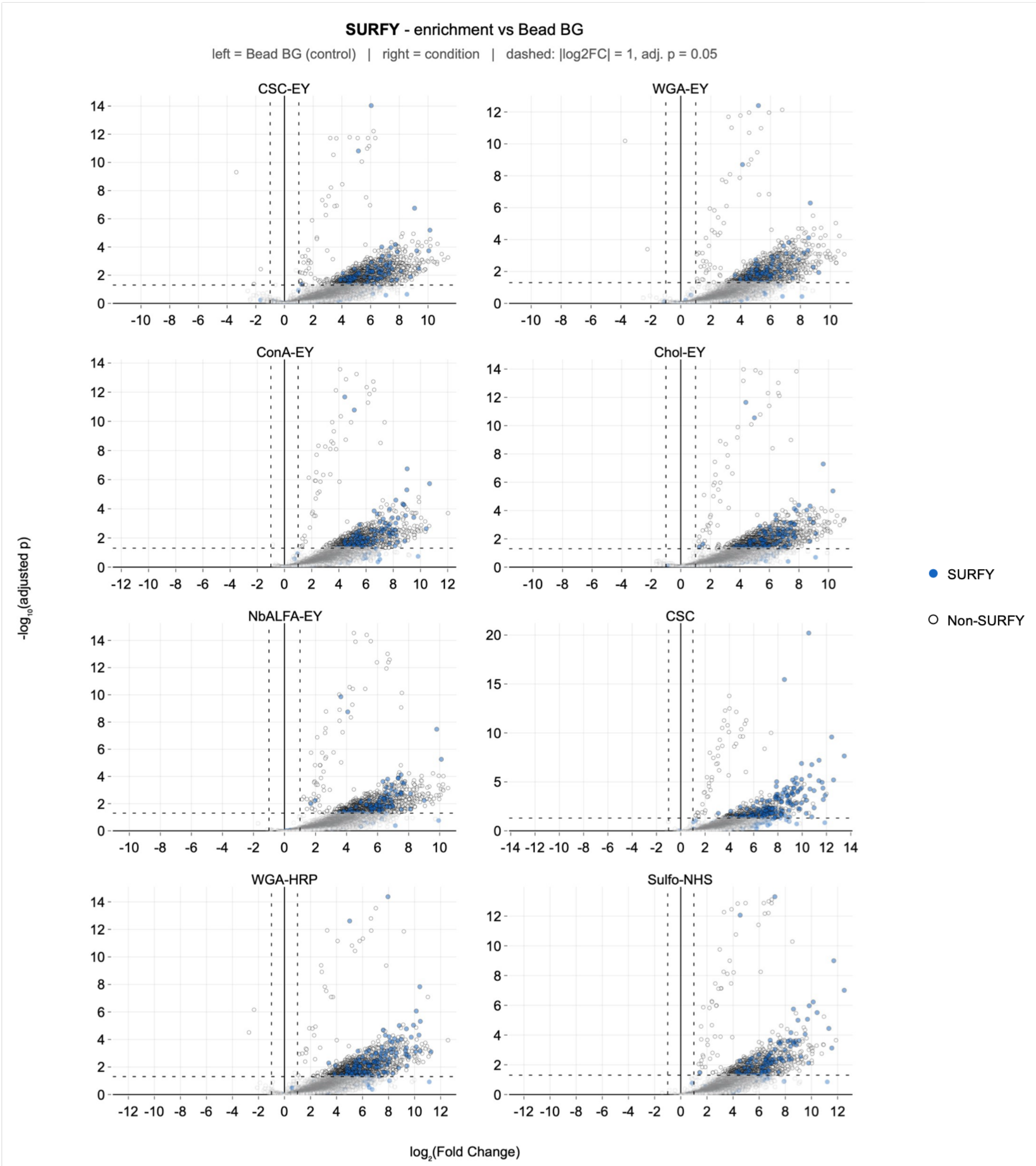

Fig. S24. Limma enrichment comparisons between surfaceomics methods and Bead BG with SURFY highlighted. Limma contrasts were performed between each surfaceomics condition and Bead BG using median normalized intensities with MNAR imputation. On each plot, the right side corresponds to the surfaceomics method and the left to Bead BG. Dashed lines are shown for the enrichment thresholds, $|\log_2(\text{fold change})| \geq 1$ and adjusted $p < 0.05$. SURFY PGs are colored blue. Note that imputed values were given reduced weights in limma, yielding suppressed p values for PGs that were fully missing in one of the compared conditions. Data reflect mean of biologic triplicate.

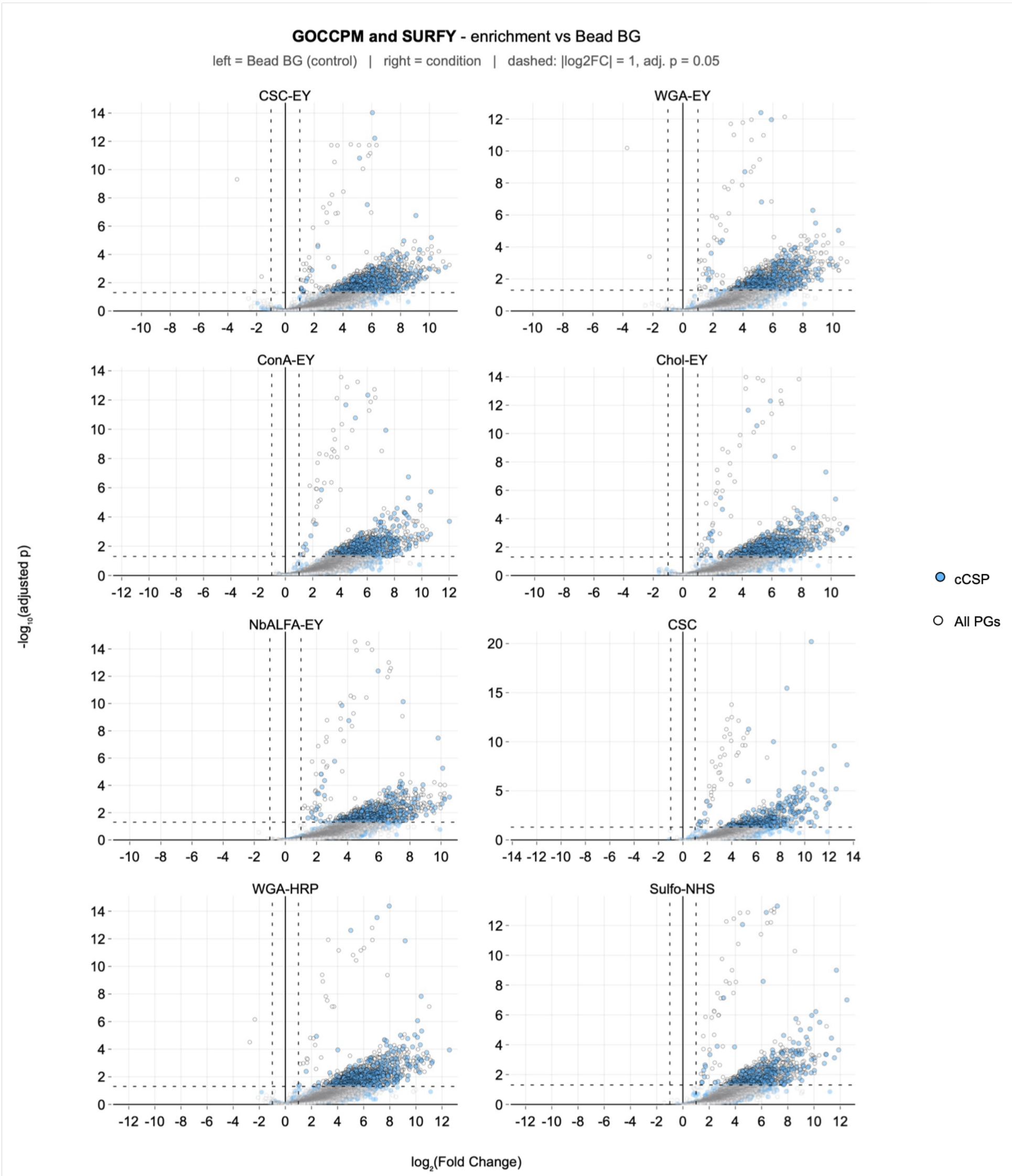

Fig. S25. Limma enrichment comparisons between surfaceomics methods and Bead BG with cCSPs highlighted. Limma contrasts were performed between each surfaceomics condition and Bead BG using median normalized intensities with MNAR imputation. On each plot, the right side corresponds to the surfaceomics method and the left to Bead BG. Dashed lines are shown for the enrichment thresholds, $|\log_2(\text{fold change})| \geq 1$ and adjusted $p < 0.05$. SURFY and GOCCPM PGs are colored light blue. Note that imputed values were given reduced weights in limma, yielding suppressed p values for PGs that were fully missing in one of the compared conditions. Data reflect mean of biologic triplicate.

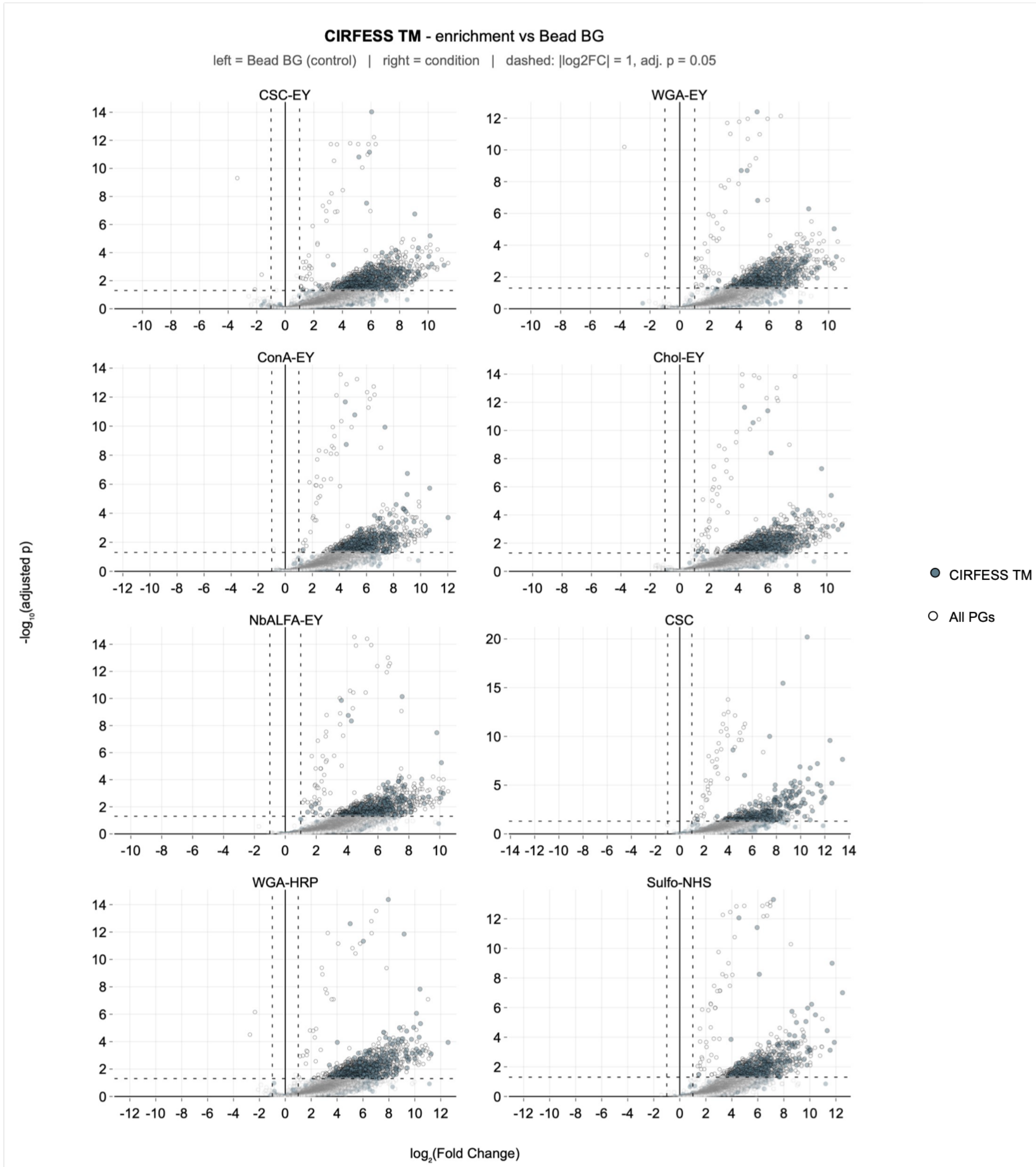

Fig. S26. Limma enrichment comparisons between surfaceomics methods and Bead BG with CIRFESS TM highlighted. Limma contrasts were performed between each surfaceomics condition and Bead BG using median normalized intensities with MNAR imputation. On each plot, the right side corresponds to the surfaceomics method and the left to Bead BG. Dashed lines are shown for the enrichment thresholds, $|\log_2(\text{fold change})| \geq 1$ and adjusted $p < 0.05$. CIRFESS TM PGs are colored blue-grey. Note that imputed values were given reduced weights in limma, yielding suppressed p values for PGs that were fully missing in one of the compared conditions. Data reflect mean of biologic triplicate.

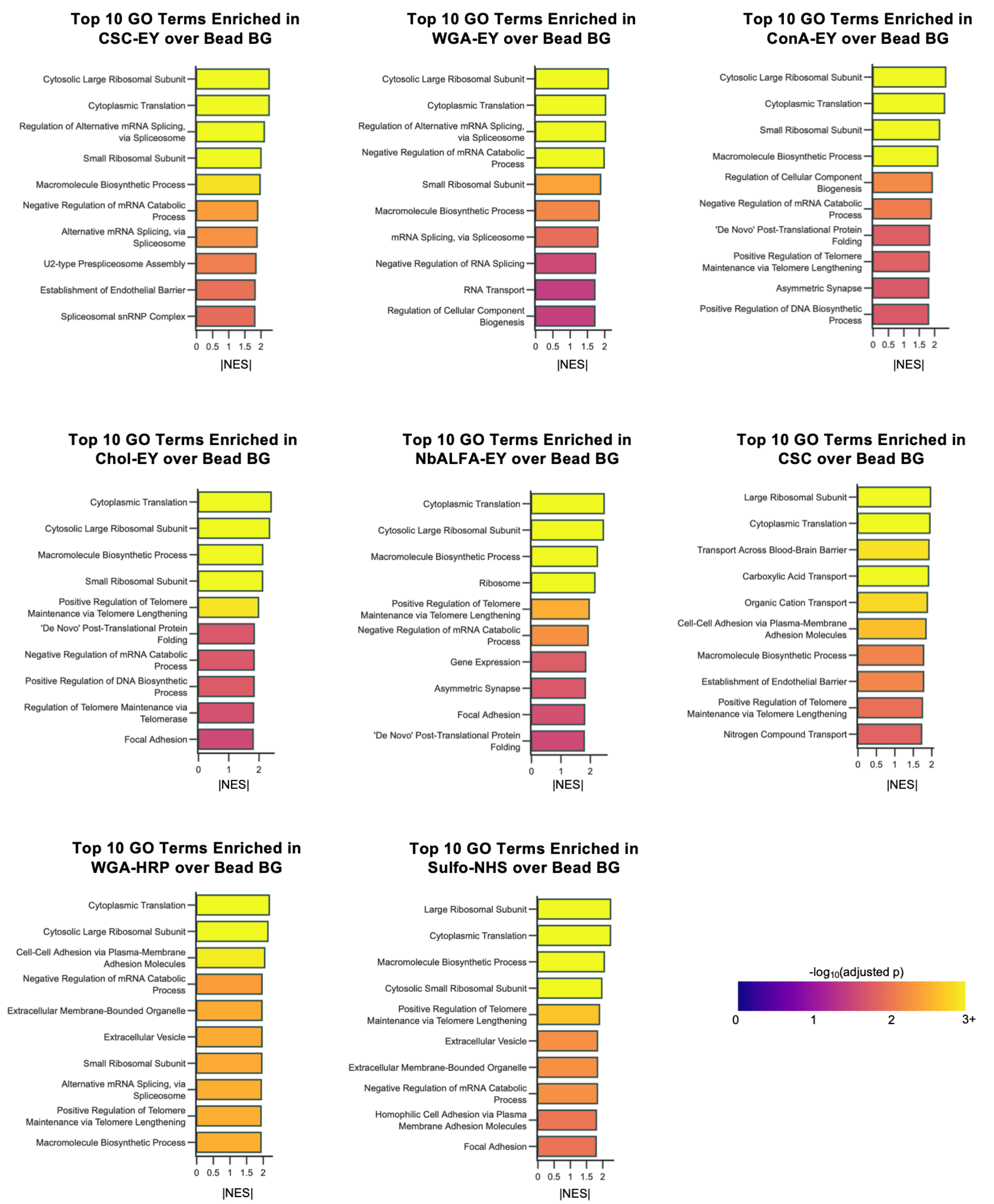

Fig. S27. Gene set enrichment analysis between surfaceomics methods and Bead BG. Median normalized intensities for all surfaceomics methods were compared pairwise to Bead BG for GSEA. The top 10 terms enriched by the surfaceomics methods with FDR < 0.05 and ordered by normalized enrichment score (NES) magnitude greater than 1.5 are shown. Note that because imputed values were given reduced weights in limma (as seen in the two plumed volcano plots in previous figures), proteins that were only seen in surfaceomics but missing in Bead BG contribute less towards which GO terms were considered top—many of such proteins are expected to be true surface proteins.

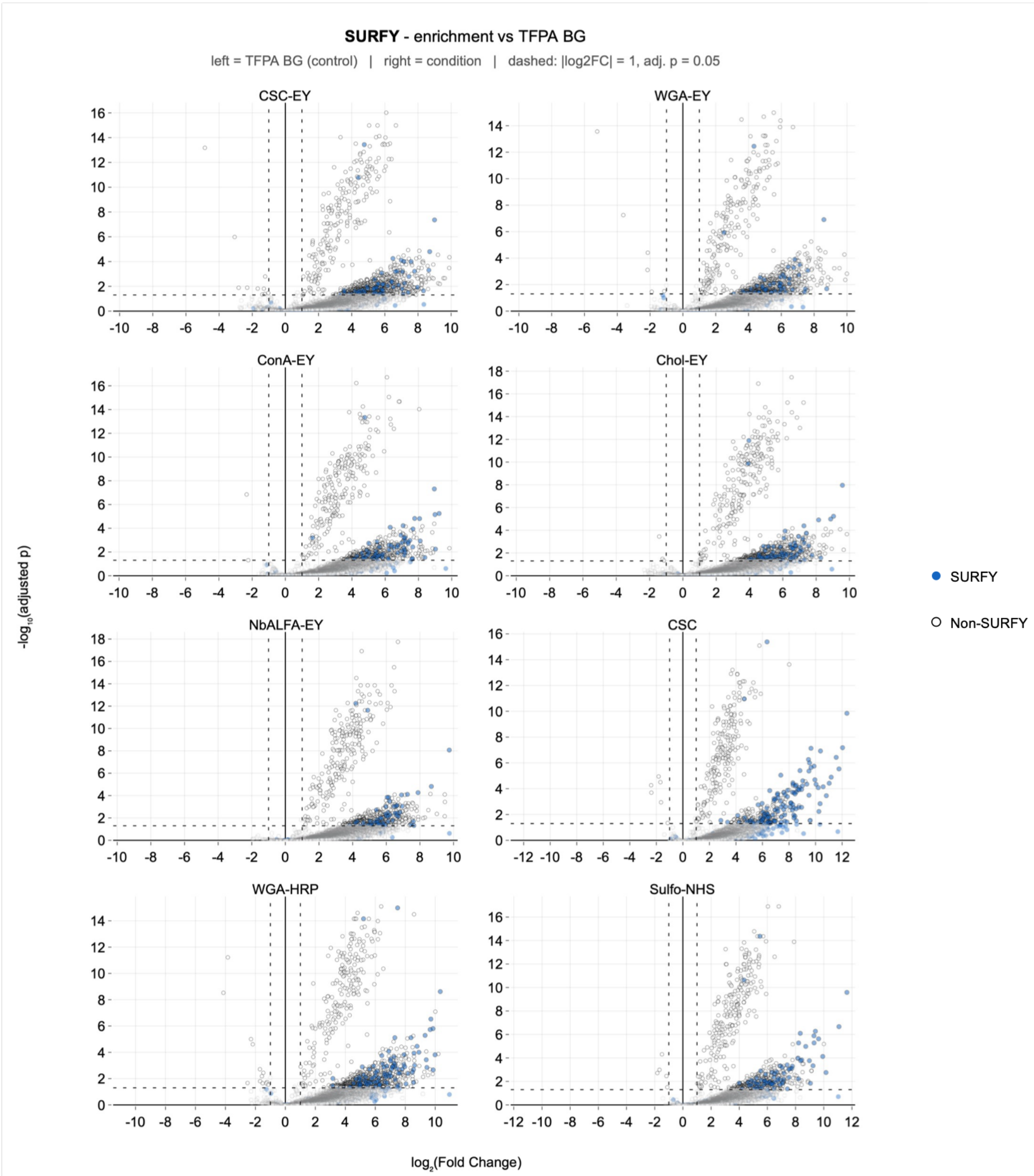

Fig. S28. Limma enrichment comparisons between surfaceomics methods and TFPA BG with SURFY highlighted. Limma contrasts were performed between each surfaceomics condition and TFPA BG using median normalized intensities with MNAR imputation. On each plot, the right side corresponds to the surfaceomics method and the left to TFPA BG. Dashed lines are shown for the enrichment thresholds, $|\log_2(\text{fold change})| \geq 1$ and adjusted $p < 0.05$. SURFY PGs are colored blue. Note that imputed values were given reduced weights in limma, yielding suppressed p values for PGs that were fully missing in one of the compared conditions. Data reflect mean of biologic triplicate.

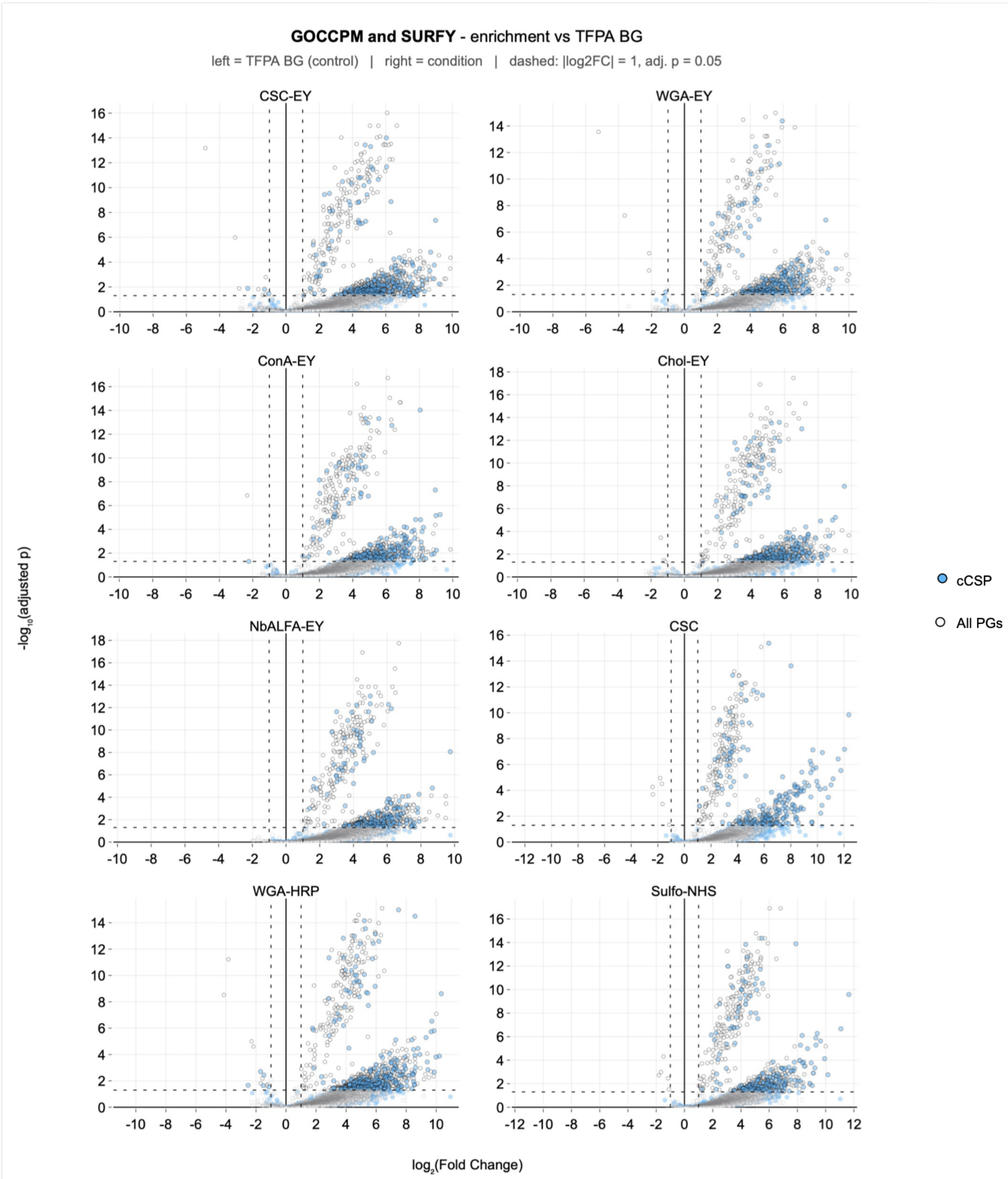

Fig. S29. Limma enrichment comparisons between surfaceomics methods and TFPA BG with cCSPs highlighted. Limma contrasts were performed between each surfaceomics condition and TFPA BG using median normalized intensities with MNAR imputation. On each plot, the right side corresponds to the surfaceomics method and the left to TFPA BG. Dashed lines are shown for the enrichment thresholds, $|\log_2(\text{fold change})| \geq 1$ and adjusted $p < 0.05$. SURFY and GOCCPM PGs are colored light blue. Note that imputed values were given reduced weights in limma, yielding suppressed p values for PGs that were fully missing in one of the compared conditions. Data reflect mean of biologic triplicate.

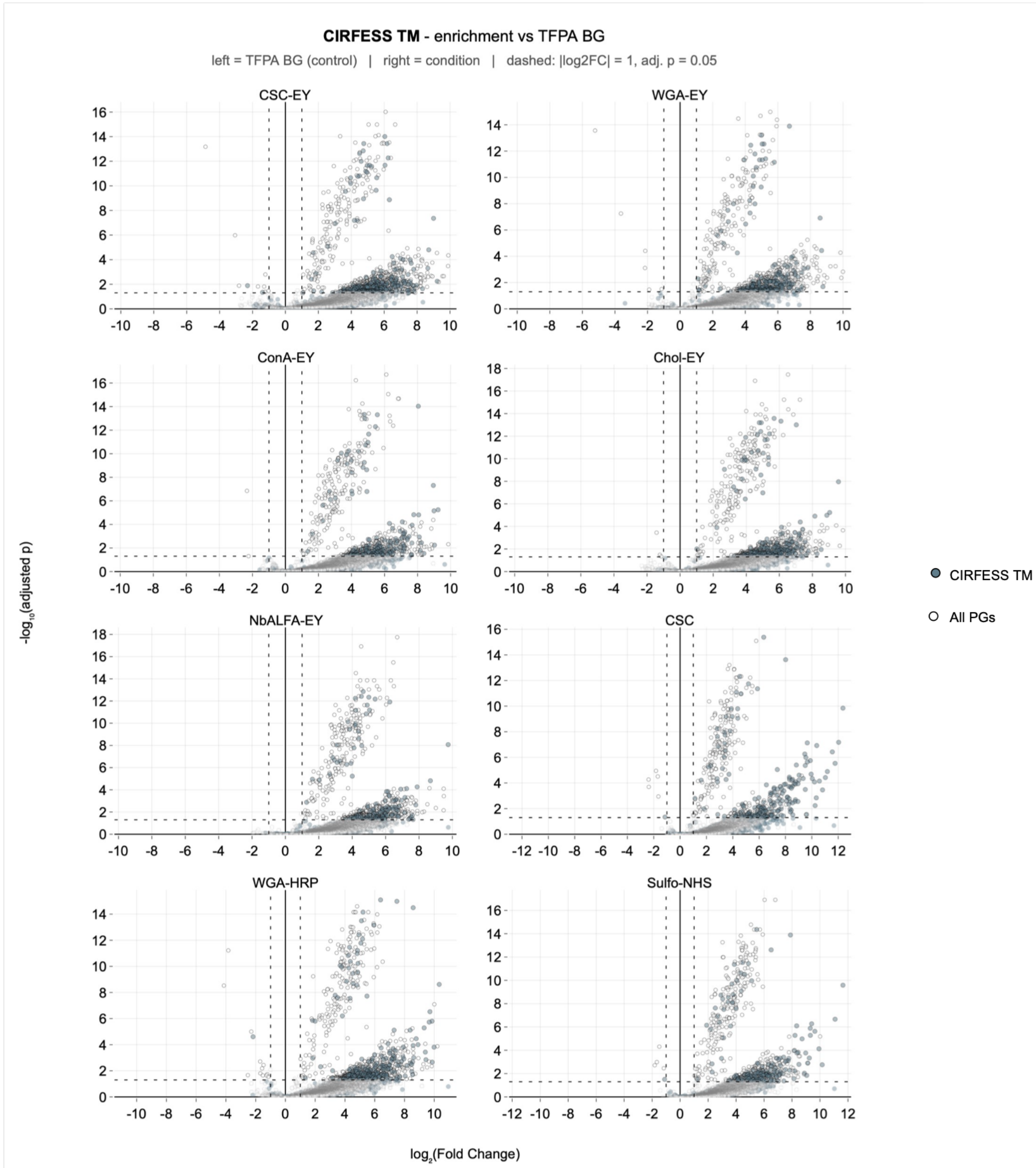

Fig. S30. Limma enrichment comparisons between surfaceomics methods and TFPA BG with CIRFESS TM highlighted. Limma contrasts were performed between each surfaceomics condition and TFPA BG using median normalized intensities with MNAR imputation. On each plot, the right side corresponds to the surfaceomics method and the left to TFPA BG. Dashed lines are shown for the enrichment thresholds, $|\log_2(\text{fold change})| \geq 1$ and adjusted $p < 0.05$. CIRFESS TM PGs are colored blue-grey. Note that imputed values were given reduced weights in limma, yielding suppressed p values for PGs that were fully missing in one of the compared conditions. Data reflect mean of biologic triplicate.

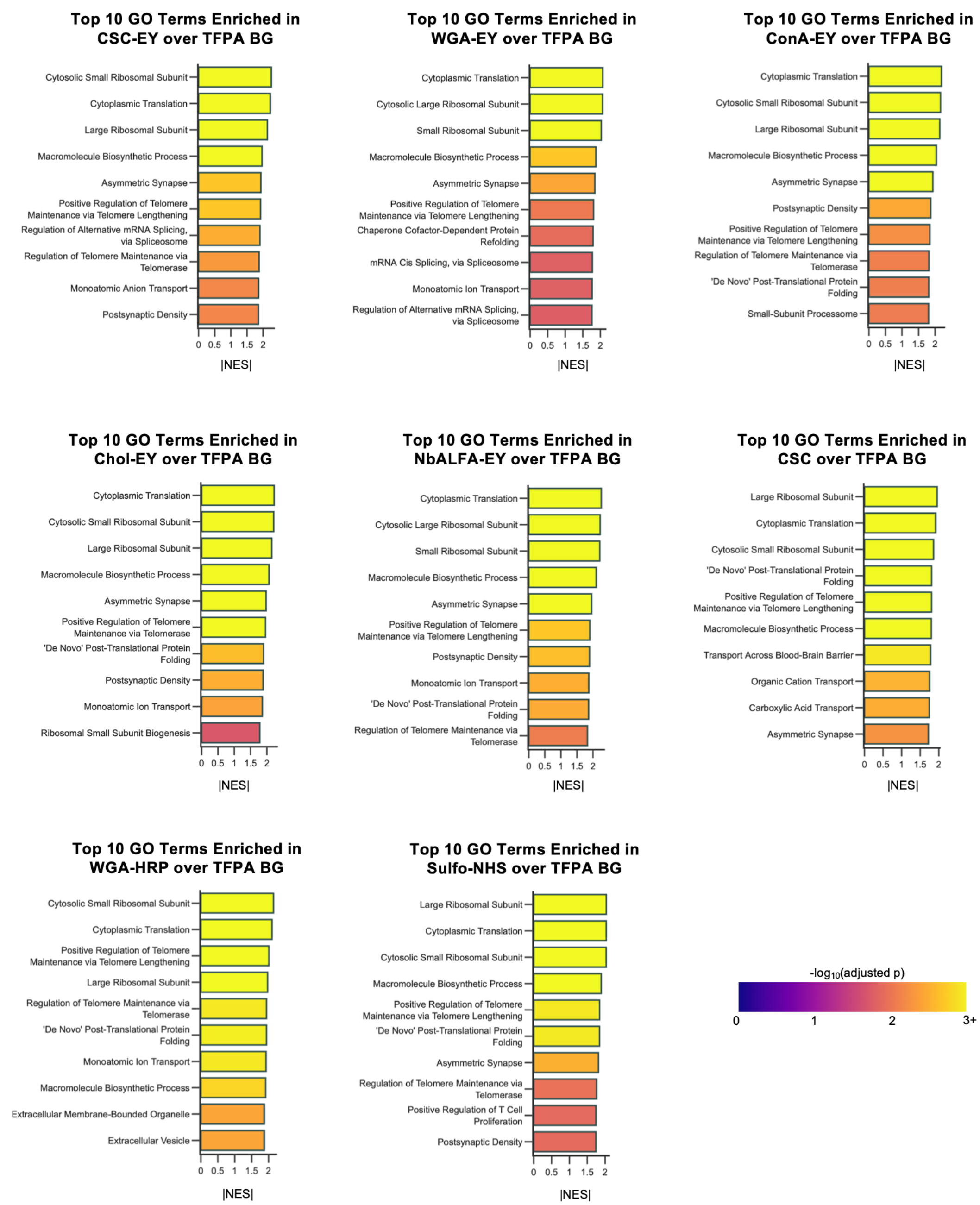

Fig. S31. Gene set enrichment analysis between surfaceomics methods and TFPA BG. Median normalized intensities for all surfaceomics methods were compared pairwise to TFPA BG for GSEA. The top 10 terms enriched by the surfaceomics methods with FDR < 0.05 and ordered by normalized enrichment score (NES) magnitude greater than 1.5 are shown. Note that because imputed values were given reduced weights in limma (as seen in the two plumed volcano plots in previous figures), proteins that were only seen in surfaceomics but missing in TFPA BG contribute less towards which GO terms were considered top—many of such proteins are expected to be true surface proteins.

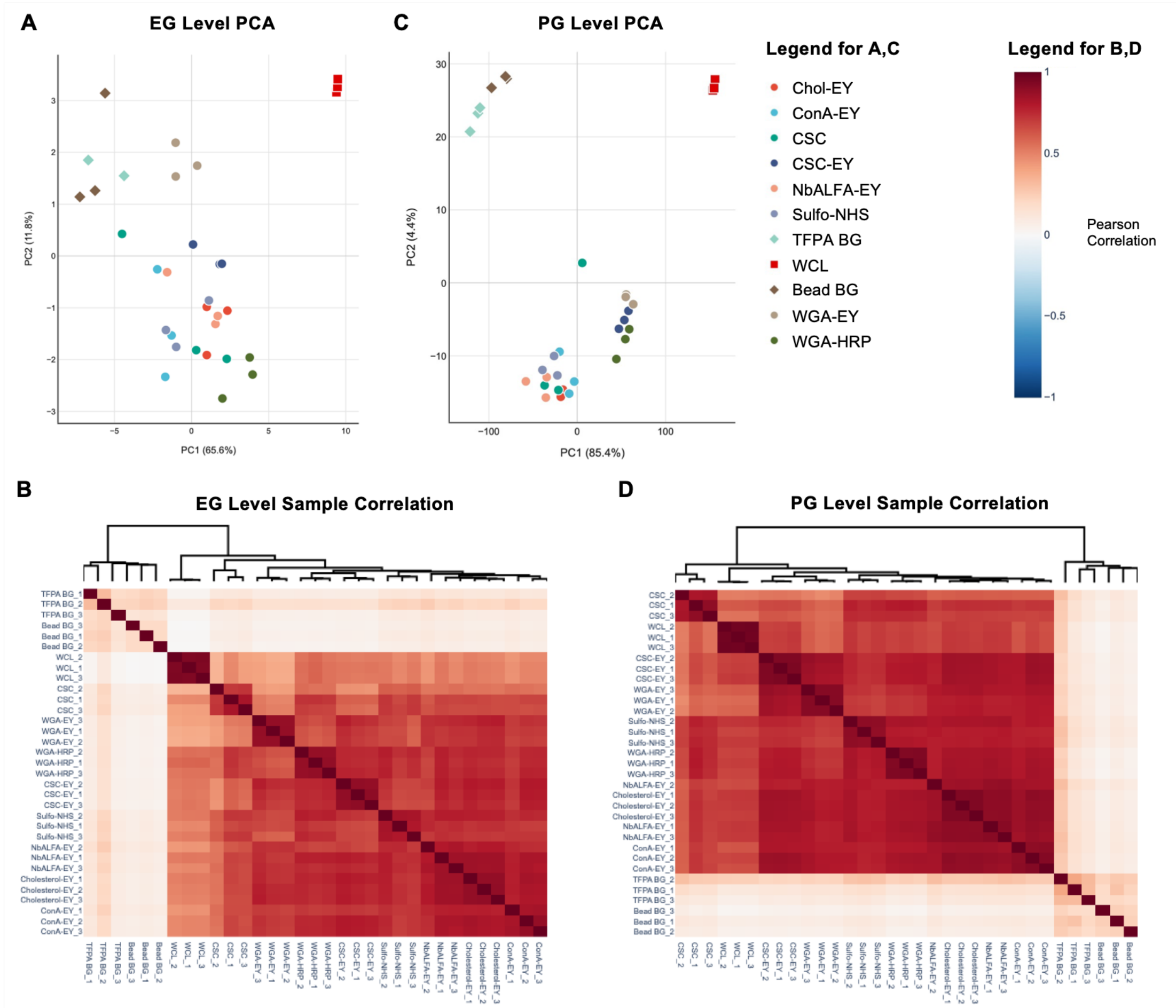

Fig. S32. Surfaceomics methods are reproducible and distinguishable from unenriched sample and technical background controls. (**A**) Principal component analysis of EG level data shows robust replicate level clustering and clustering by sample type. Surfaceomics, circles; unenriched, squares; background controls, diamonds. MNAR imputation was used for missing values. (**B**) Heatmap of EG-level Pearson correlation between samples highlights dissimilarity of background controls and unenriched WCL from all surfaceomics methods. (**C**) PG-level PCA separates unenriched WCL and background controls from surfaceomics methods in PC1 and PC2. Surfaceomics, circles; unenriched, squares; background controls, diamonds. MNAR imputation was used for missing values. (**D**) PG-level Pearson correlations recapitulate EG-level distinction of background controls from surfaceomics samples, although CSC shows even greater dissimilarity to other surfaceomics methods than WCL.

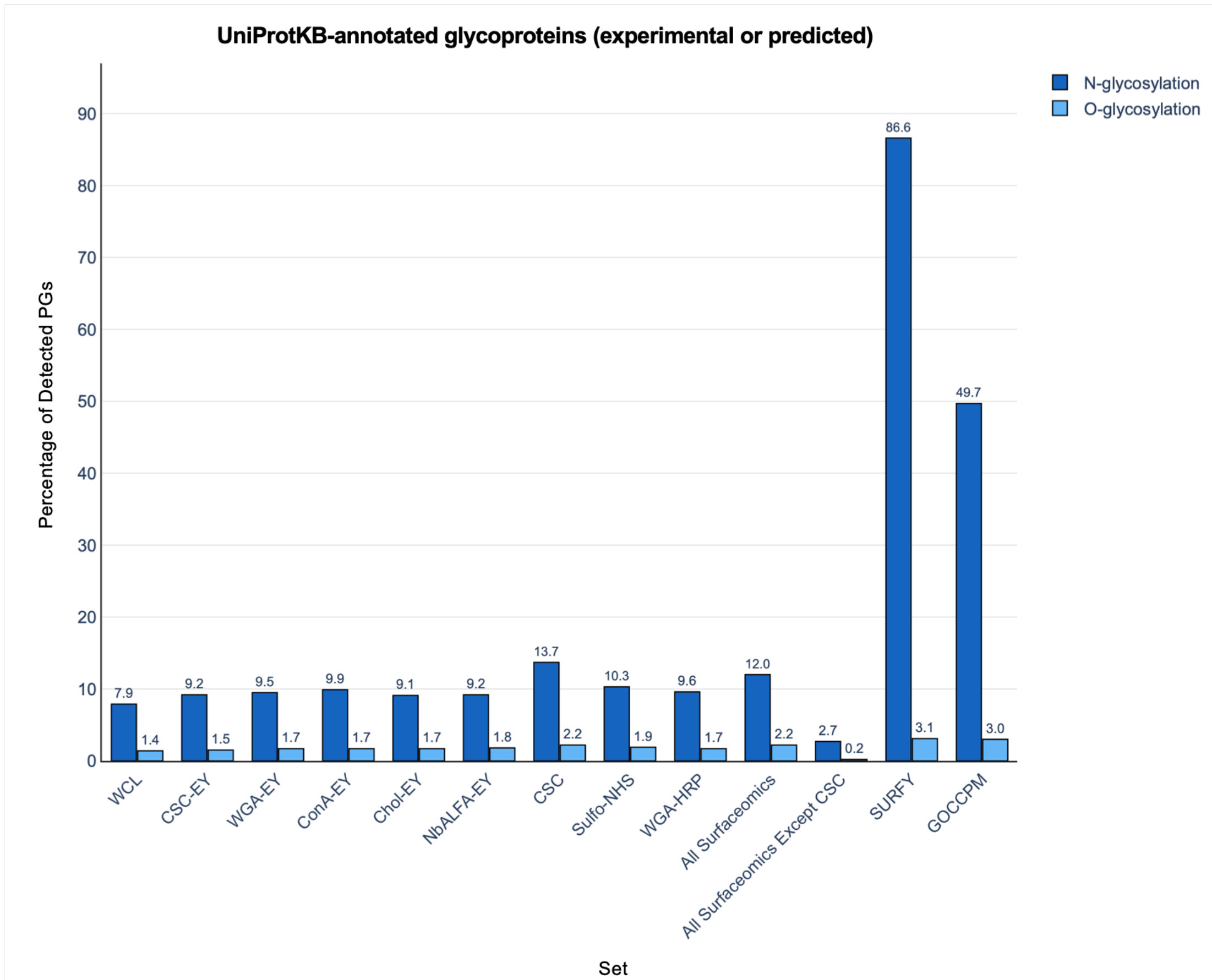

Fig. S33. Surfaceomics-detected PGs are more likely to be N-glycosylated than WCL-detected PGs. PGs detected in each condition were searched for N-glycosylation or O-glycosylation annotations in UniProtKB (15). The percentage of all PGs by identity within that condition that had such an annotation is plotted. WCL showed lower N-glycosylation percentage than any surfaceomics method, with CSC showing the highest N-glycosylation percentage. The PGs shared by all surfaceomics methods except CSC had a particularly low N-glycosylation rate. The cCSP reference lists were heavily N-glycosylated, as expected.

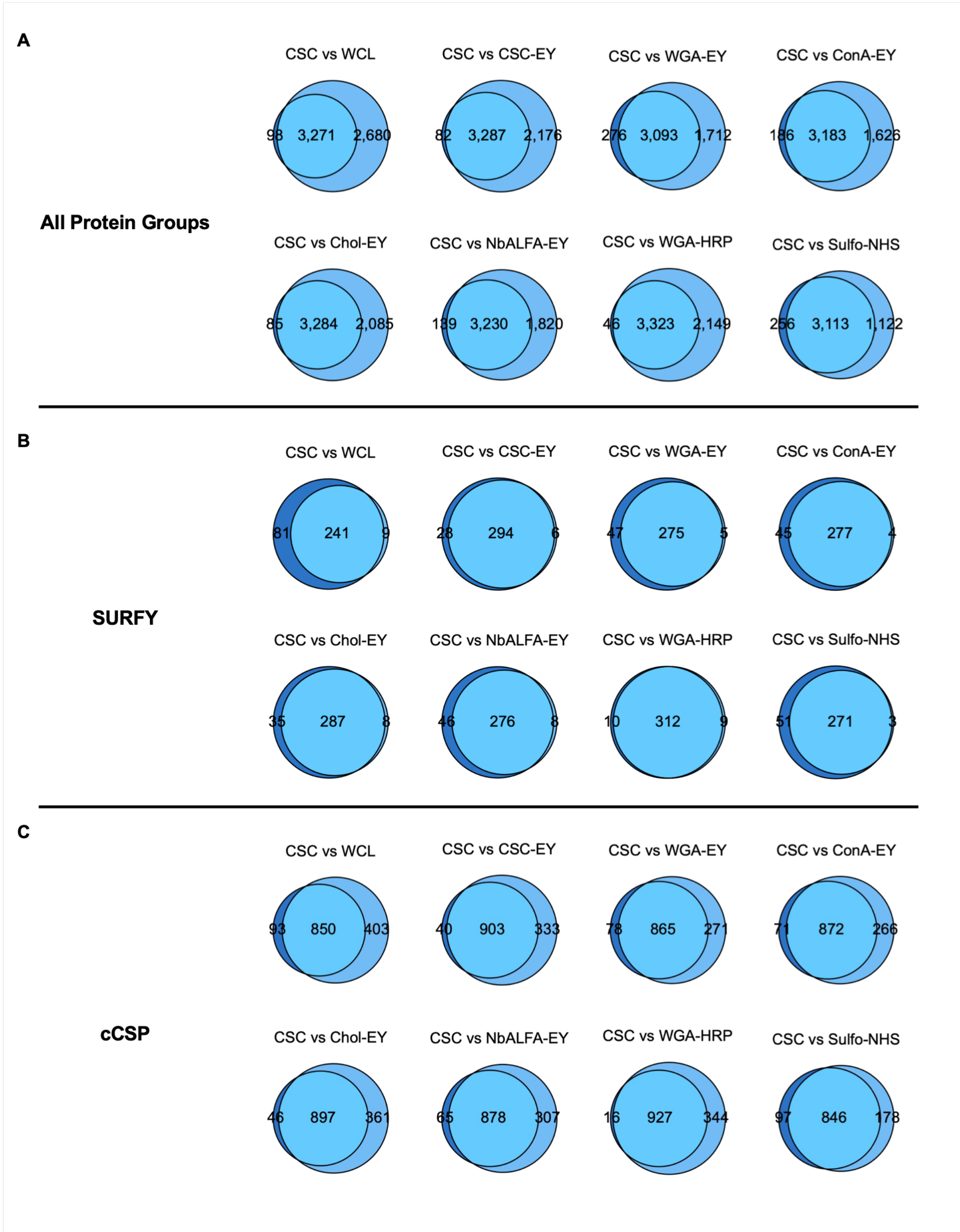

Fig. S34. CSC detects fewer PGs than other surfaceomics methods overall but superiorly detects SURFY PGs. Venn diagram comparison of detected PGs in CSC compared to WCL and every other surfaceomics method, restricting to (**A**) All PGs, (**B**) SURFY PGs, or (**C**) cCSPs. No method is ever a complete subset of another, but when restricted to SURFY, CSC detects more proteins than other surfaceomics methods or WCL.

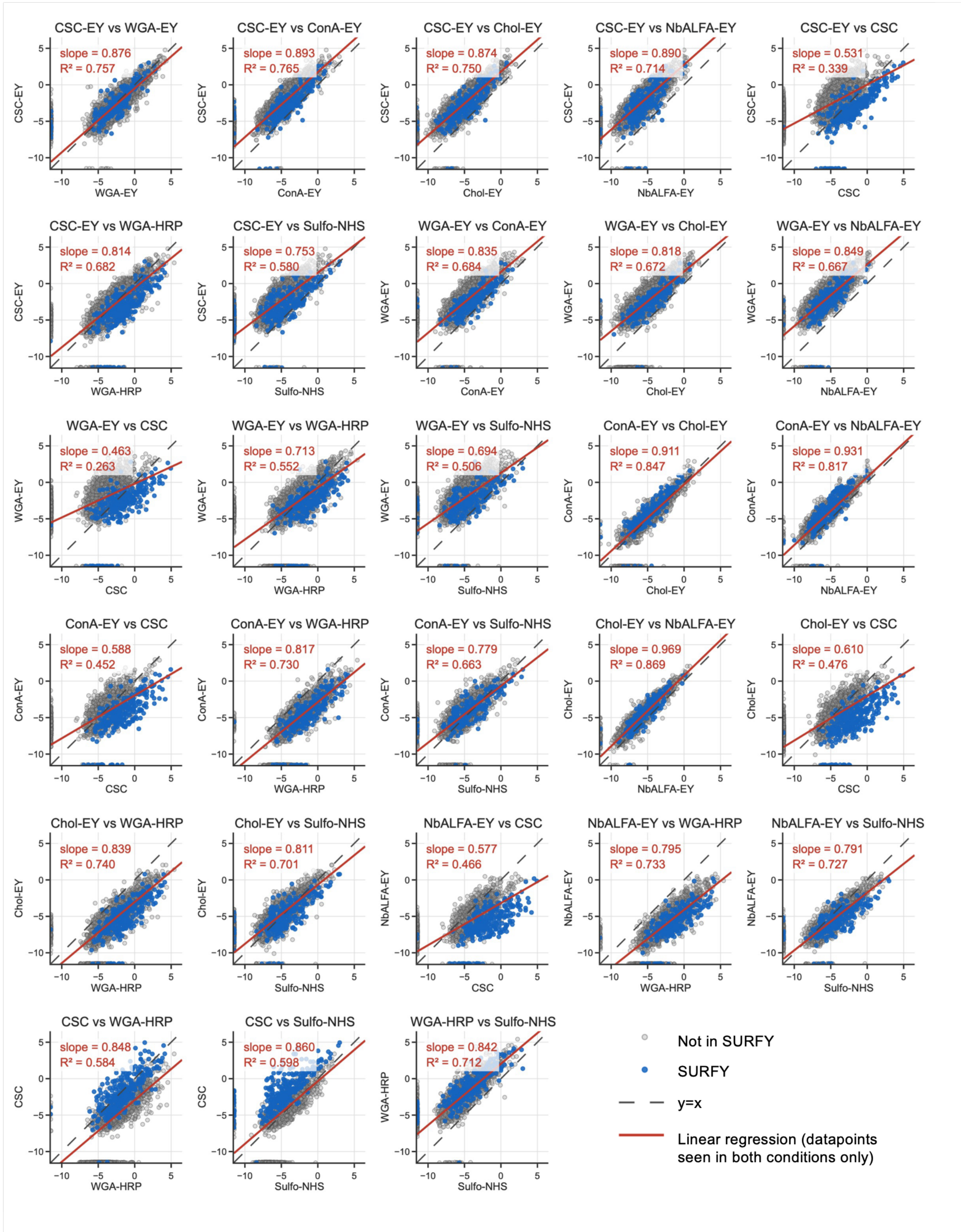

Fig. S35. Scatterplots reveal unequal labeling efficiency and skewed SURFY detection across surfaceomics methods. Endogenous biotin normalized intensities of PGs detected by each surfaceomics method are plotted against their intensities in another surfaceomics method. PGs that were detected in one surfaceomics condition but not the other are clamped to axis. The line of equality is shown as a dashed line and the linear regression for PGs detected by both surfaceomics methods is shown as a solid red line. Disparities in biotinylation efficiency are visible in deviation of the linear regression from the line of equality. SURFY PGs are colored blue

and skew on one side of the linear regression line reflects increased relative detection by a surfaceomics method, e.g. with CSC. Intensities are the average of biological triplicate with MNAR imputed values for missing replicates.

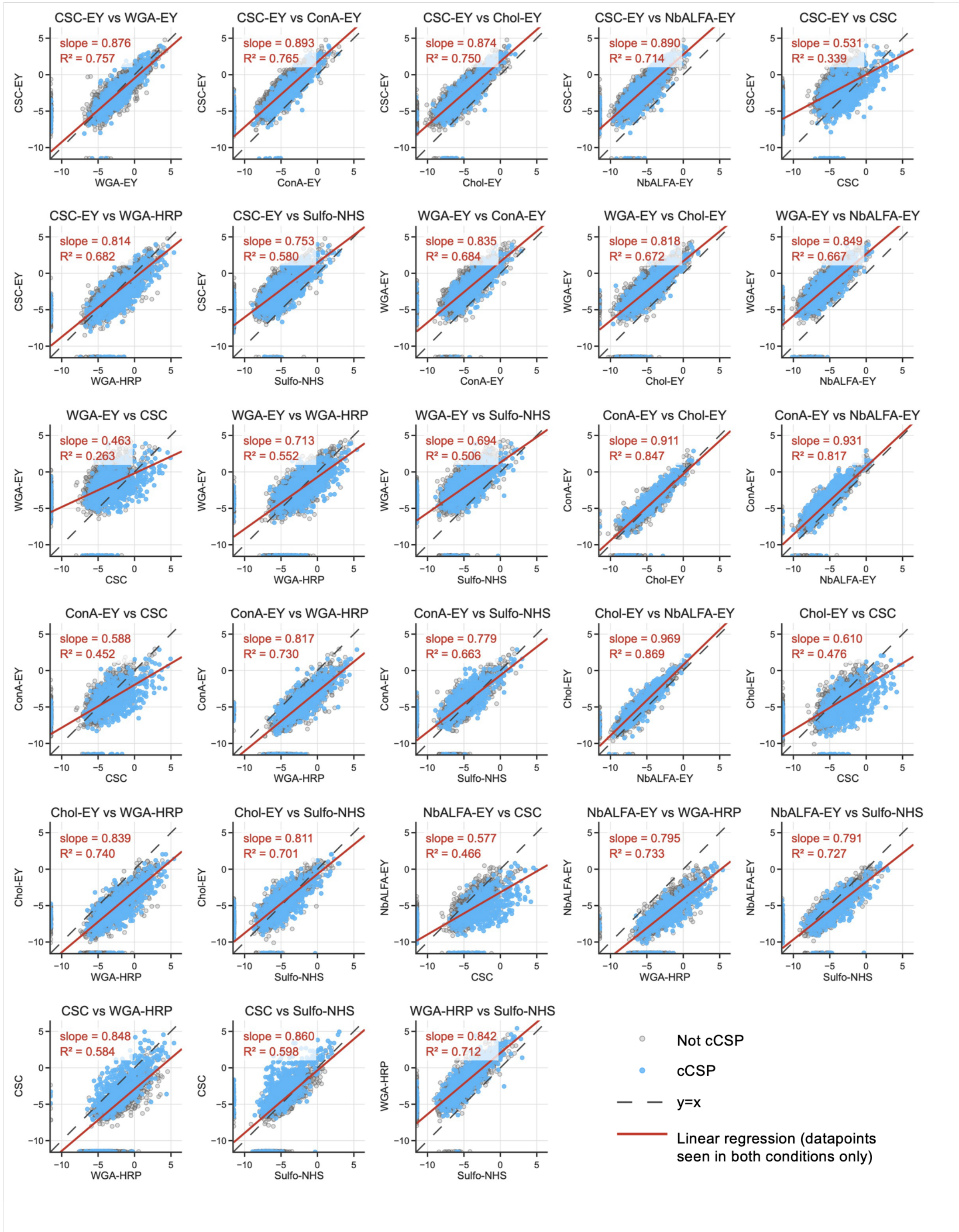

Fig. S36. Scatterplots comparing surfaceomics methods, colored by cCSPs, show nonequivalence between surfaceomics methods that all detect cCSPs. Endogenous biotin normalized intensities of PGs detected by each surfaceomics method are plotted against their intensities in another surfaceomics method. PGs that were detected in one surfaceomics condition but not the other are clamped to axis. The line of equality is shown as a dashed line and the linear regression for PGs detected by both surfaceomics methods is shown as a solid red line. Disparities in biotinylation efficiency are visible in deviation of the linear regression from the line of

equality. cCSP PGs are colored light blue but unlike with SURFY PGs do not strongly skew on one side of the linear regression line. Intensities are the average of biological triplicate with MNAR imputed values for missing replicates.

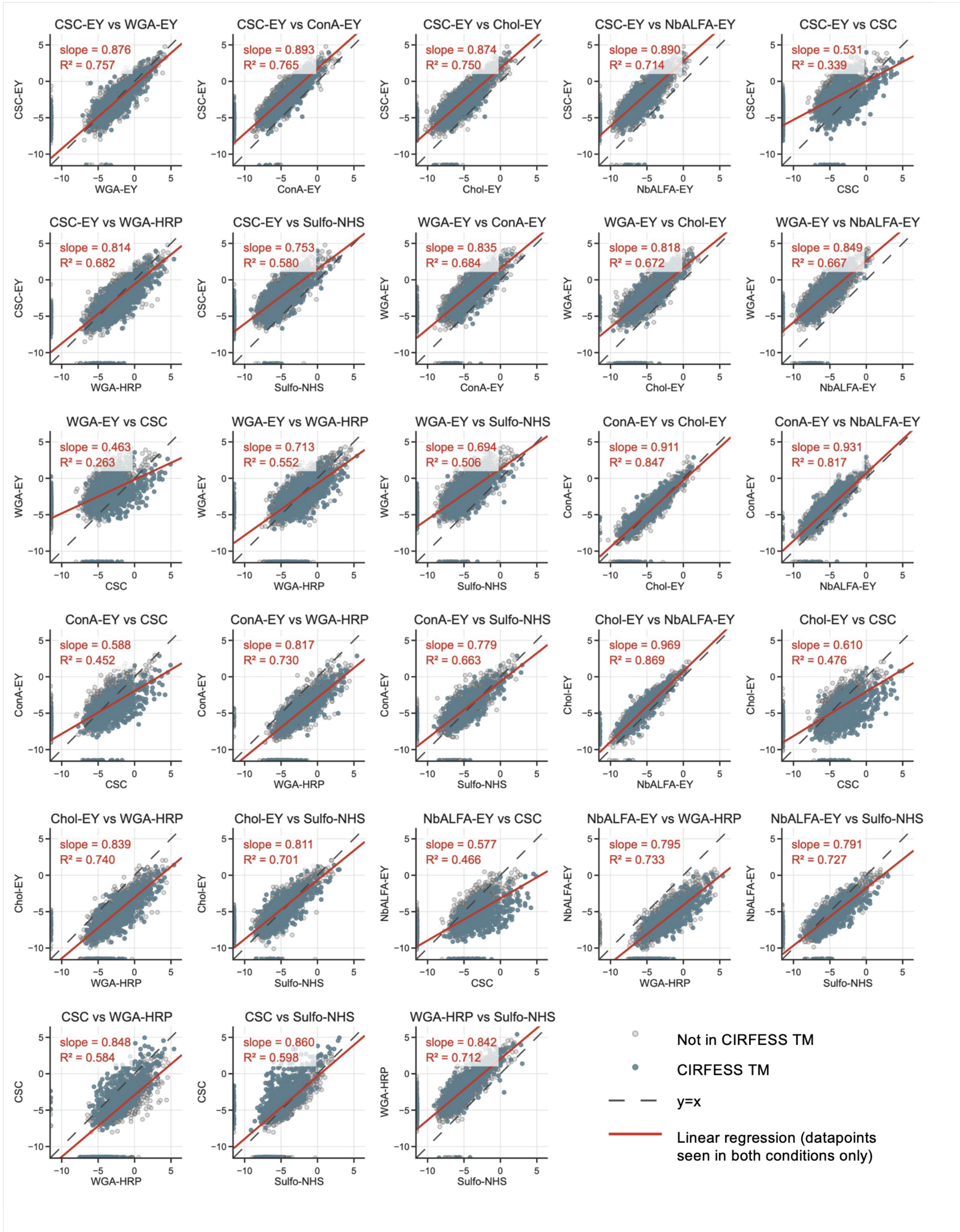

Fig. S37. Scatterplots comparing surfaceomics methods highlighting CIRFESS TM PGs recapitulate cCSP trends. Endogenous biotin normalized intensities of PGs detected by each surfaceomics method are plotted against their intensities in another surfaceomics method. PGs that were detected in one surfaceomics condition but not the other are clamped to axis. The line of equality is shown as a dashed line and the linear regression for PGs detected by both surfaceomics methods is shown as a solid red line. Disparities in biotinylation efficiency are visible in deviation of the linear regression from the line of equality. CIRFESS TM PGs are

colored blue-grey and distribute on both sides of linear regression, akin to cCSPs. Intensities are the average of biological triplicate with MNAR imputed values for missing replicates.

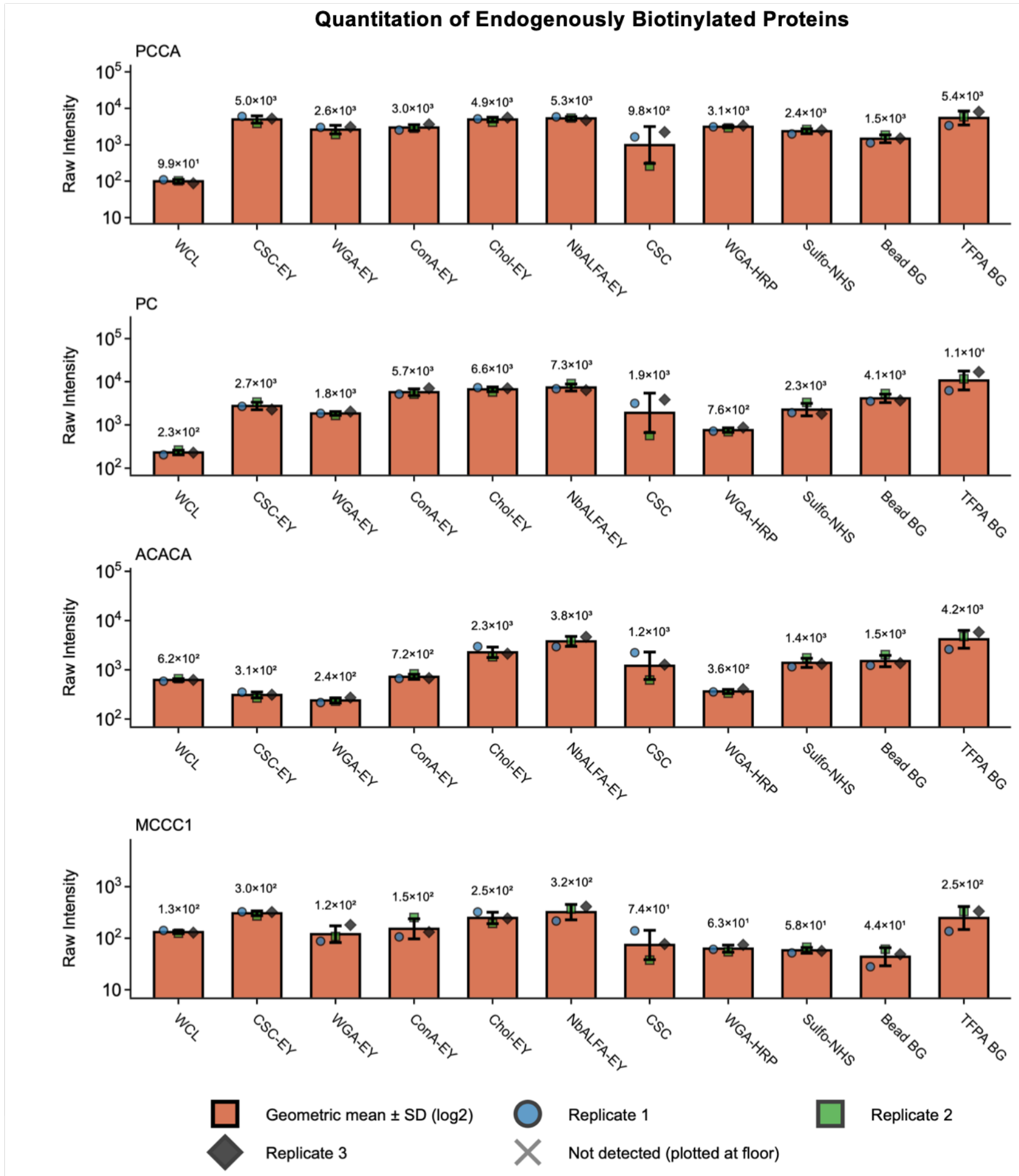

Fig. S38. Quantitation of endogenously biotinylated proteins varies per protein across samples. The raw intensities of known endogenously biotinylated proteins in each sample are plotted along with their geometric mean. Note that for each sample, 200 ng total peptide from the on-bead digest was injected, meaning that enrichment samples with greater artificial biotinylation are expected to have decreased endogenous biotin quantification. As an unenriched sample, WCL is expected to show relatively lower levels of these endogenously biotinylated proteins. Data are reflective of biologic triplicates. Standard deviation error bars were calculated in $\log_2$ space then reverse transformed into linear scale. All presented endogenously biotinylated proteins were quantified in every replicate of every sample.

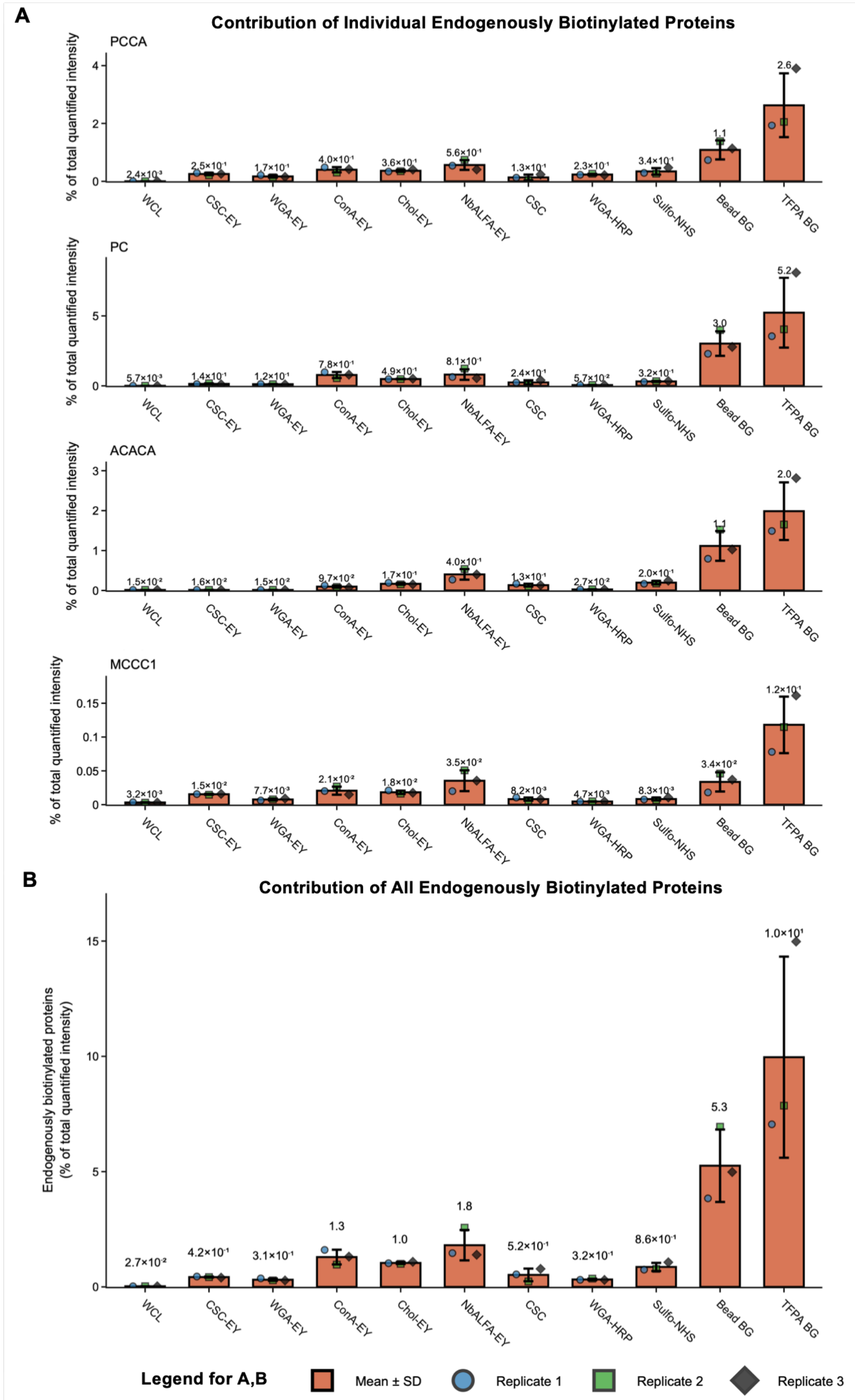

Fig. S39. Endogenously biotinylated proteins contribute a small fraction of total quantified intensity for surfaceomics samples. (**A**) The percentage of raw total PG intensity in each sample contributed by each endogenously biotinylated protein is plotted. The mean percentage of biologic triplicates with standard deviation is shown. (**B**) The summed contribution of endogenously biotinylated proteins is shown for each condition. As expected, the negative BG controls show the highest percentage of endogenous biotinylation. Note that this analysis was performed on unfiltered raw data, meaning that common contaminants were also considered in

the total intensity denominator. The mean percentage of biologic triplicates with standard deviation is shown.

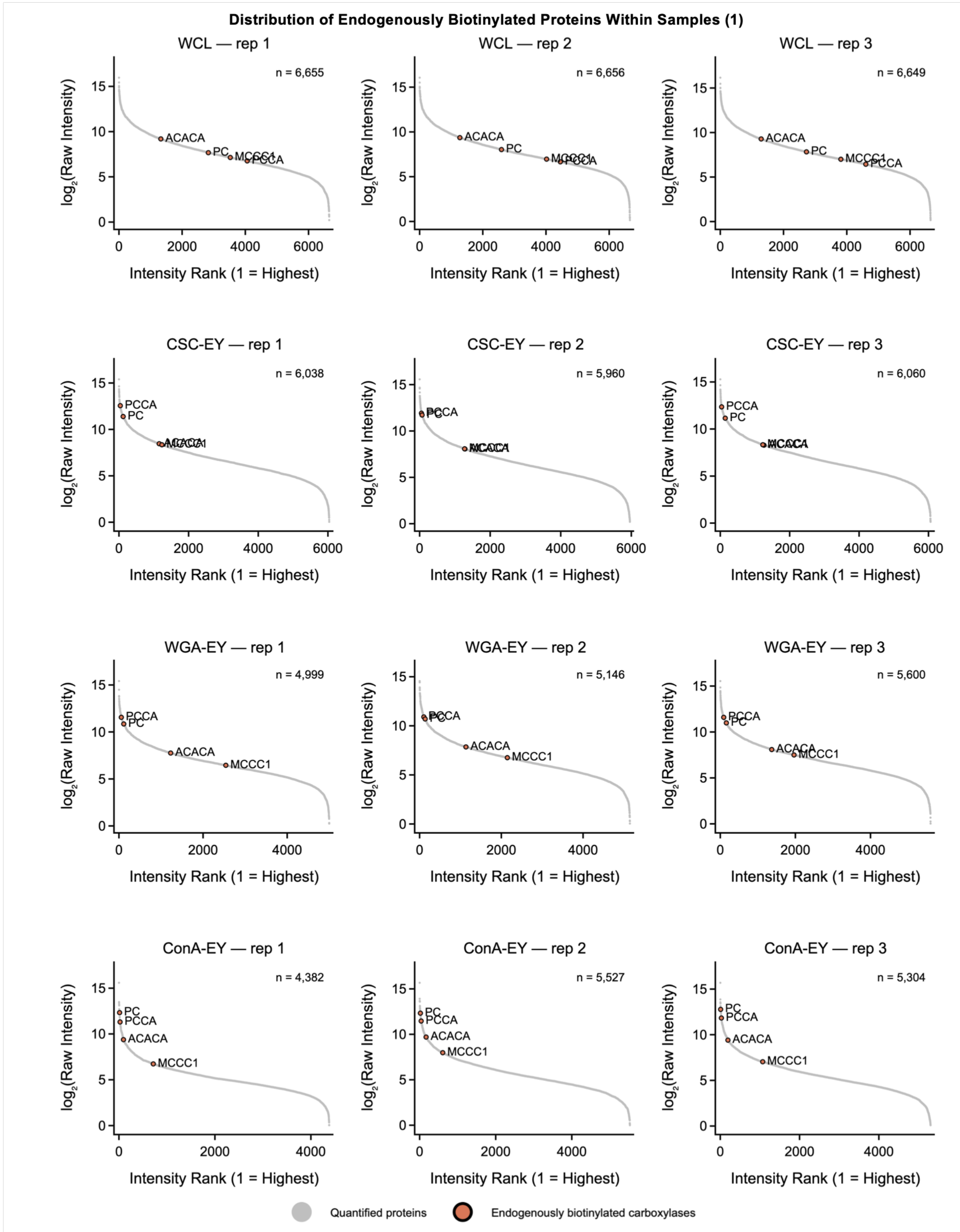

Fig. S40. Endogenously biotinylated proteins are high abundance in enriched samples. The raw intensities in each individual sample are shown as a waterfall plot. Endogenously biotinylated proteins are highlighted in orange. Shown here are the waterfalls for WCL, CSC-EY, WGA-EY, and ConA-EY.

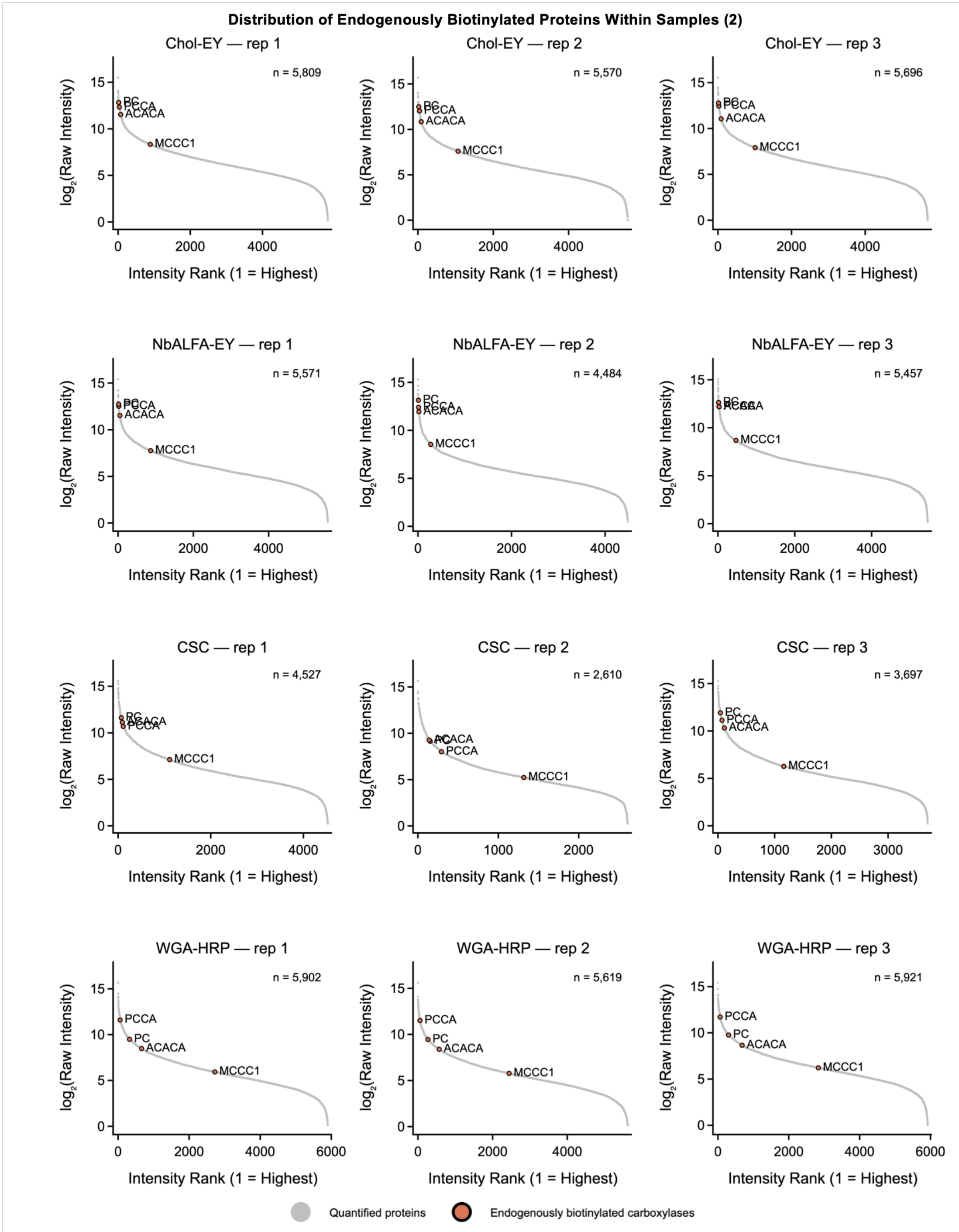

Fig. S41. Endogenously biotinylated proteins are high abundance in enriched samples. The raw intensities in each individual sample are shown as a waterfall plot. Endogenously biotinylated proteins are highlighted in orange. Shown here are the waterfalls for Chol-EY, NbALFA-EY, CSC, and WGA-HRP.

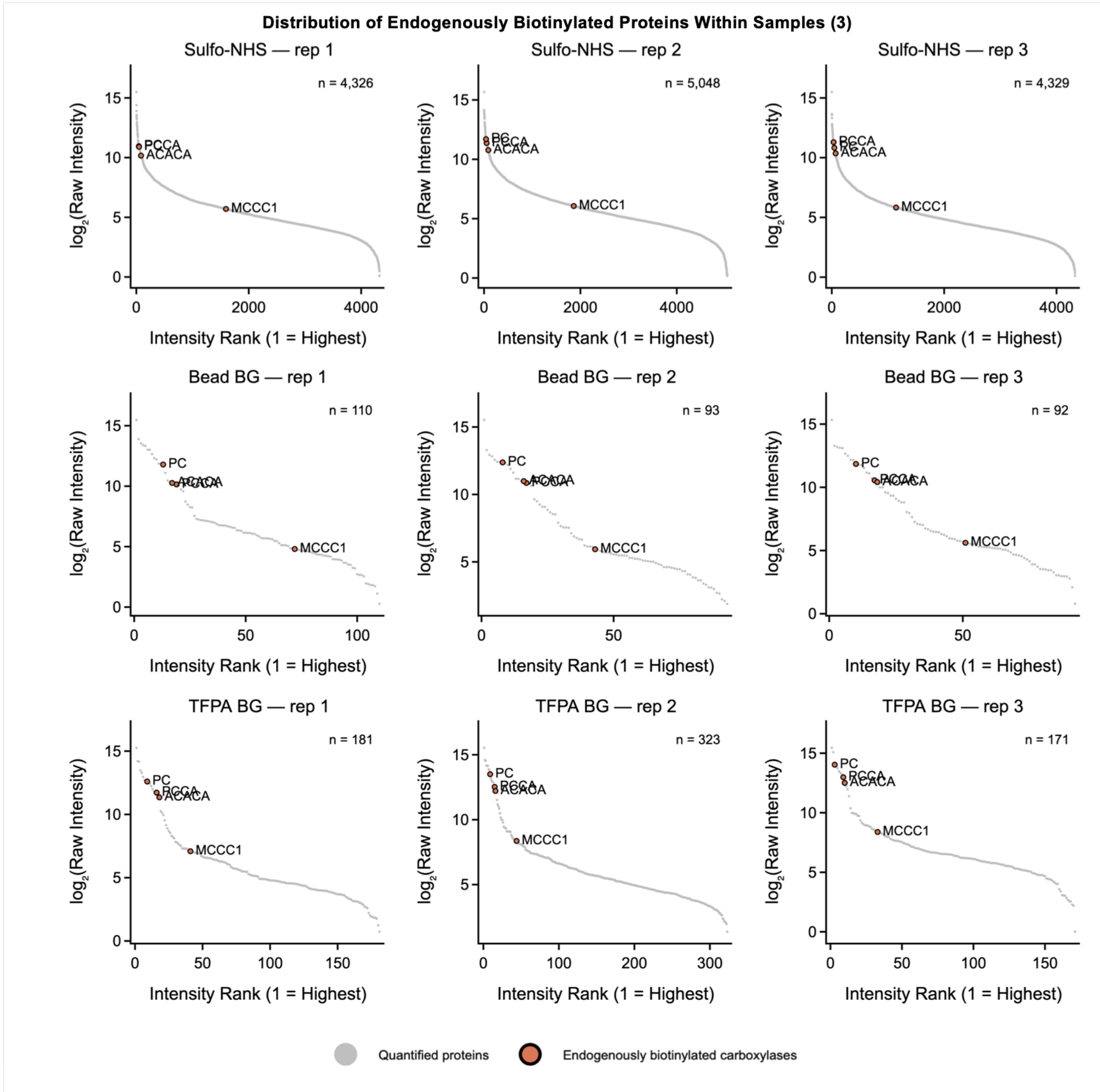

Fig. S42. Endogenously biotinylated proteins are high abundance in enriched samples. The raw intensities in each individual sample are shown as a waterfall plot. Endogenously biotinylated proteins are highlighted in orange. Shown here are the waterfalls for Sulfo-NHS and the negative controls Bead BG and TFPA BG.

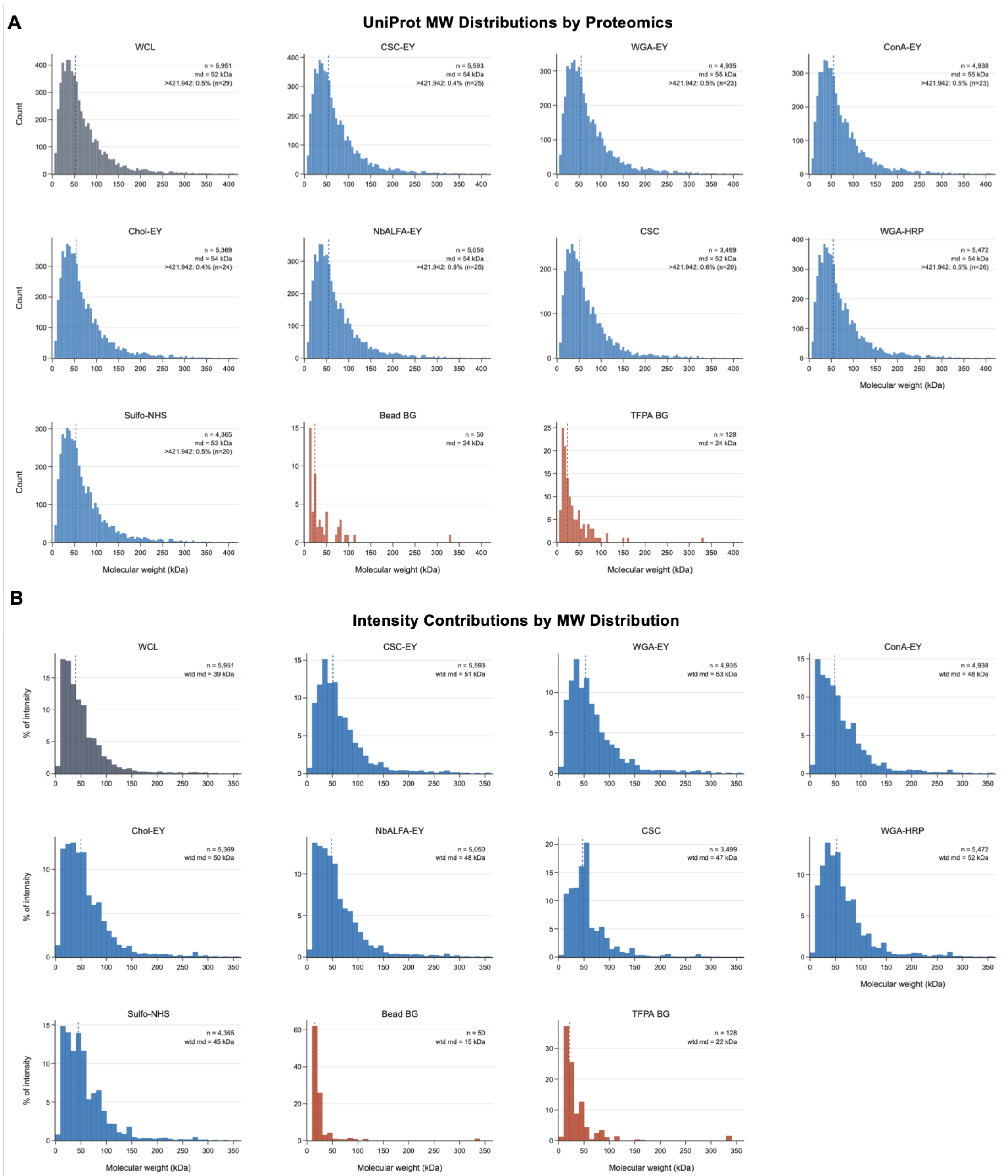

Fig. S43. Molecular weight distributions vary across surfaceomics samples by quantitation but not detection. (**A**) PGs detected in each sample are plotted by UniProtKB sourced molecular weights (MWs). The overall distribution of detected proteins is similar across all surfaceomics samples and WCL, although it differs notably for the BG negative controls. (**B**) The fraction of total sample intensity contributed by PGs with UniProtKB annotated MW within defined bins is plotted. When intensity is considered, the distributions across different samples differ, although they are not reflective of the distributions previously seen by Western blot of the same samples.

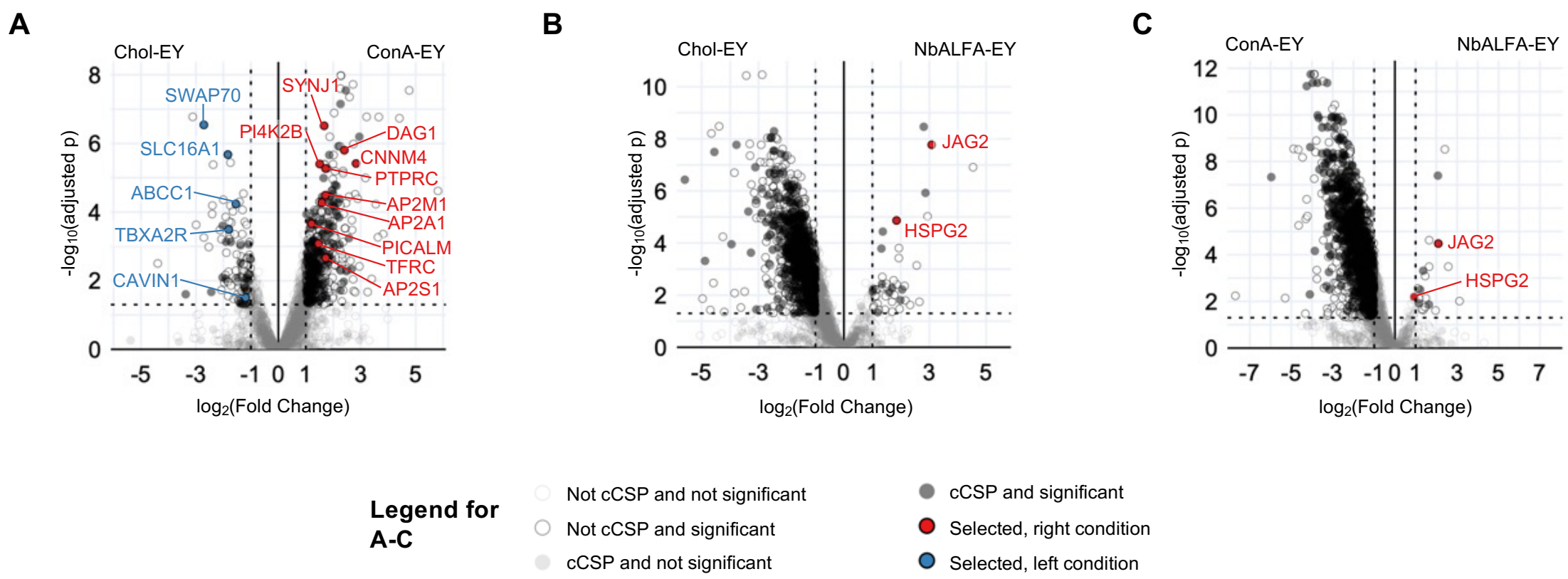

Fig. S44. Volcano plot analysis affirms mechanistic differences and disparate labeling efficiencies among Chol-EY, ConA-EY, and NbALFA-EY. (**A**) Volcano plot comparing ConA-EY and Chol-EY shows that Chol-EY relatively enriches proteins known to associate with cholesterol-rich microdomains, while ConA-EY relatively enriches endocytosis-relevant proteins and proteins. (**B**) Volcano plot comparing NbALFA-EY to Chol-EY shows that many proteins are significantly enriched by Chol-EY over NbALFA-EY. Among the few proteins NbALFA-EY preferentially detects are JAG2 and HSPG2. (**C**) Volcano plot comparing NbALFA-EY to ConA-EY indicates superior identification of proteins by ConA-EY, many of which are cCSPs.

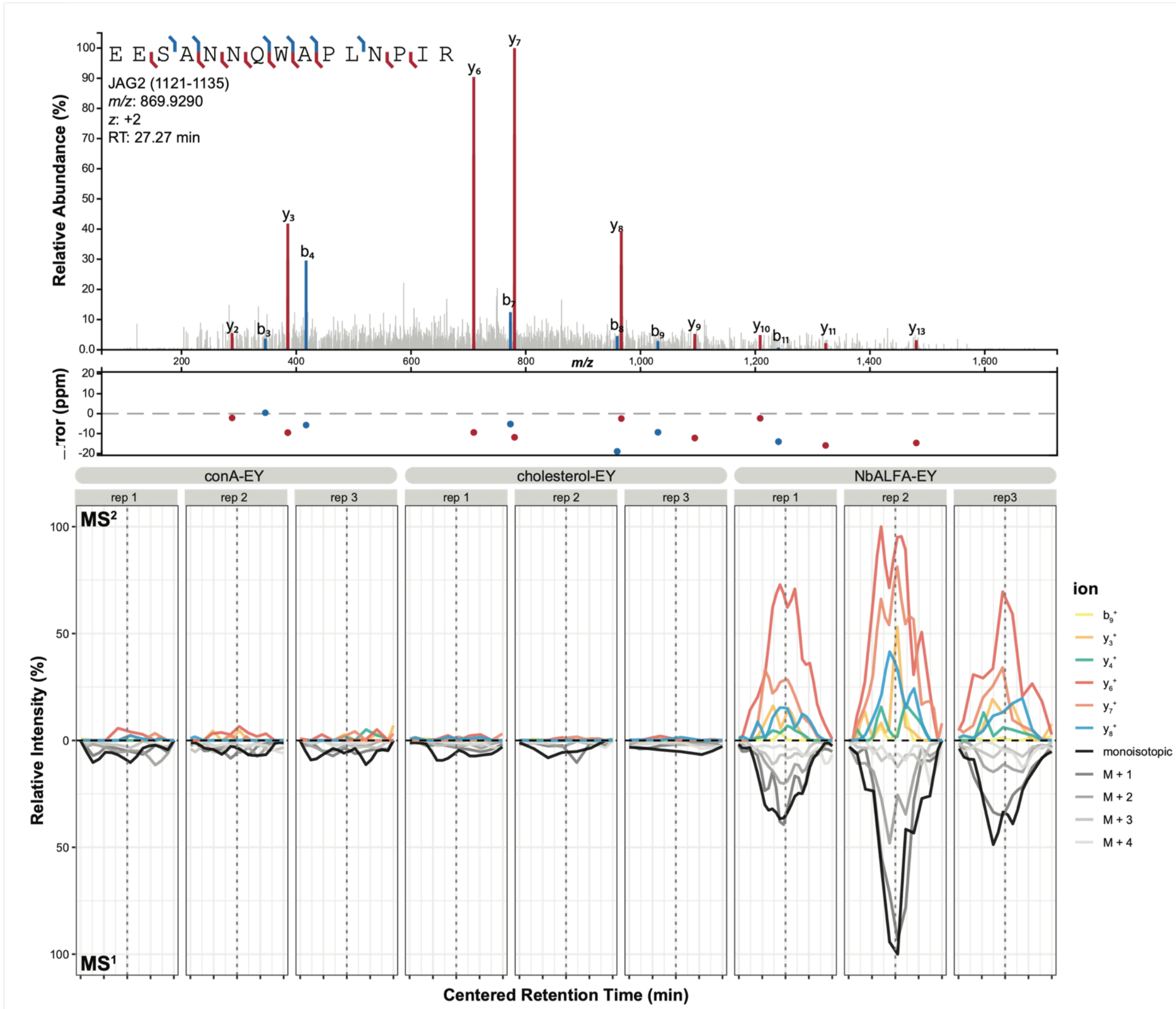

Fig. S45. The protrusive cCSP JAG2 is well captured by NbALFA-EY. Top: Mass spectrum of JAG2 peptide EESANNQWAPLNPIR with identified b and y ions highlighted. **Middle:** Parts per million (ppm) error of each detected b and y ion. **Bottom:** Ion intensities normalized to the highest intensity seen from JAG2 peptide EESANNQWAPLNPIR across ConA-EY, Chol-EY, and NbALFA-EY. Top y axis is MS2, whereas bottom y axis is MS1. X axis is retention time centered on the peak elution time for each ion and each sample.

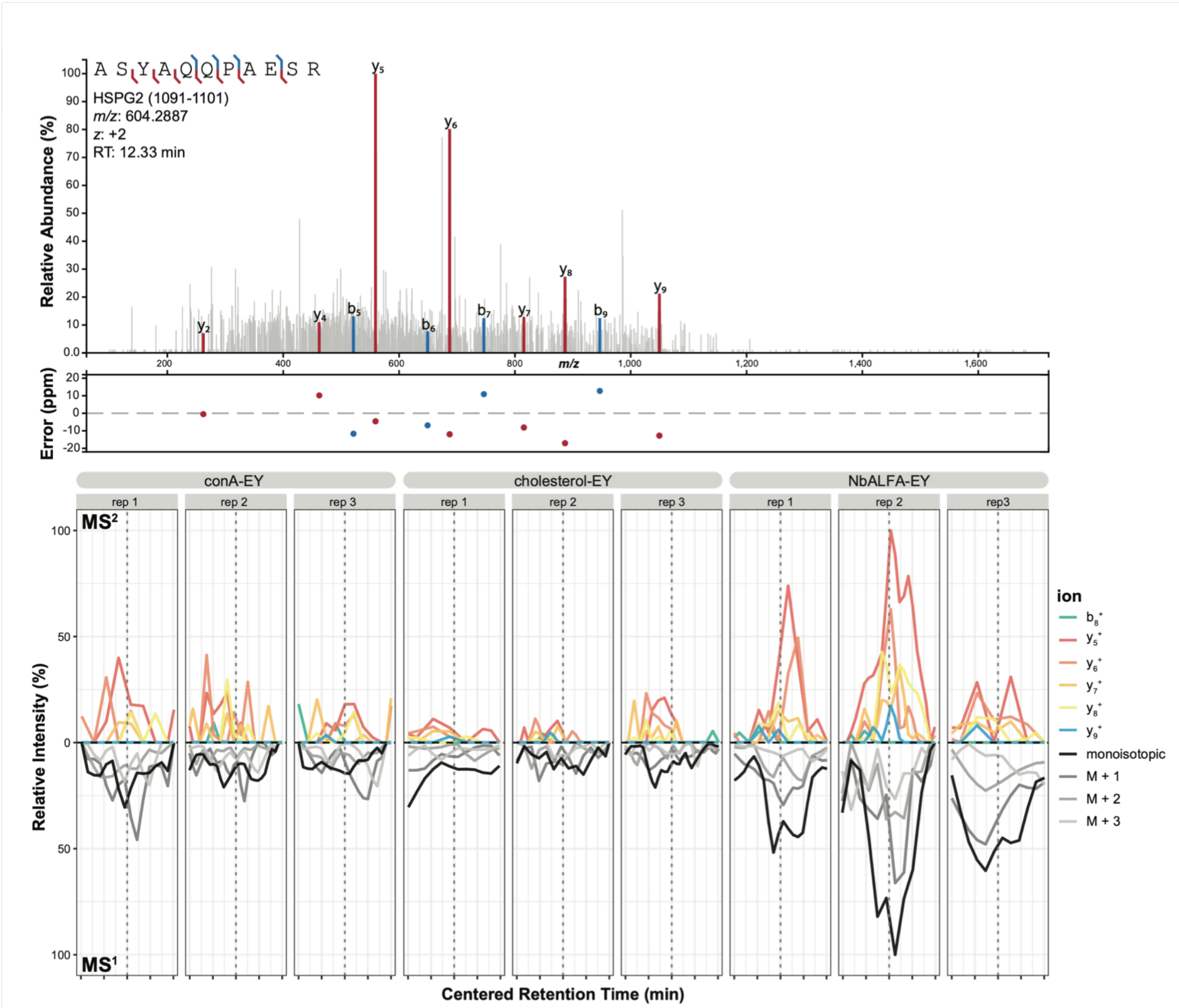

Fig. S46. Large cCSP HSPG2 is better captured by NbALFA-EY than ConA-EY or Chol-EY despite being glycosylated. Top: Mass spectrum of HSPG2 peptide ASYAQQPAESR with identified b and y ions highlighted. **Middle:** Parts per million (ppm) error of each detected b and y ion. **Bottom:** Ion intensities normalized to the highest intensity seen from ASYAQQPAESR across ConA-EY, Chol-EY, and NbALFA-EY. Top y axis is MS2, whereas bottom y axis is MS1. X axis is retention time centered on the peak elution time for each ion and each sample. Note that ConA-EY also quantifies this peptide, albeit to a lesser degree; HSPG2 is glycosylated and also quite large.

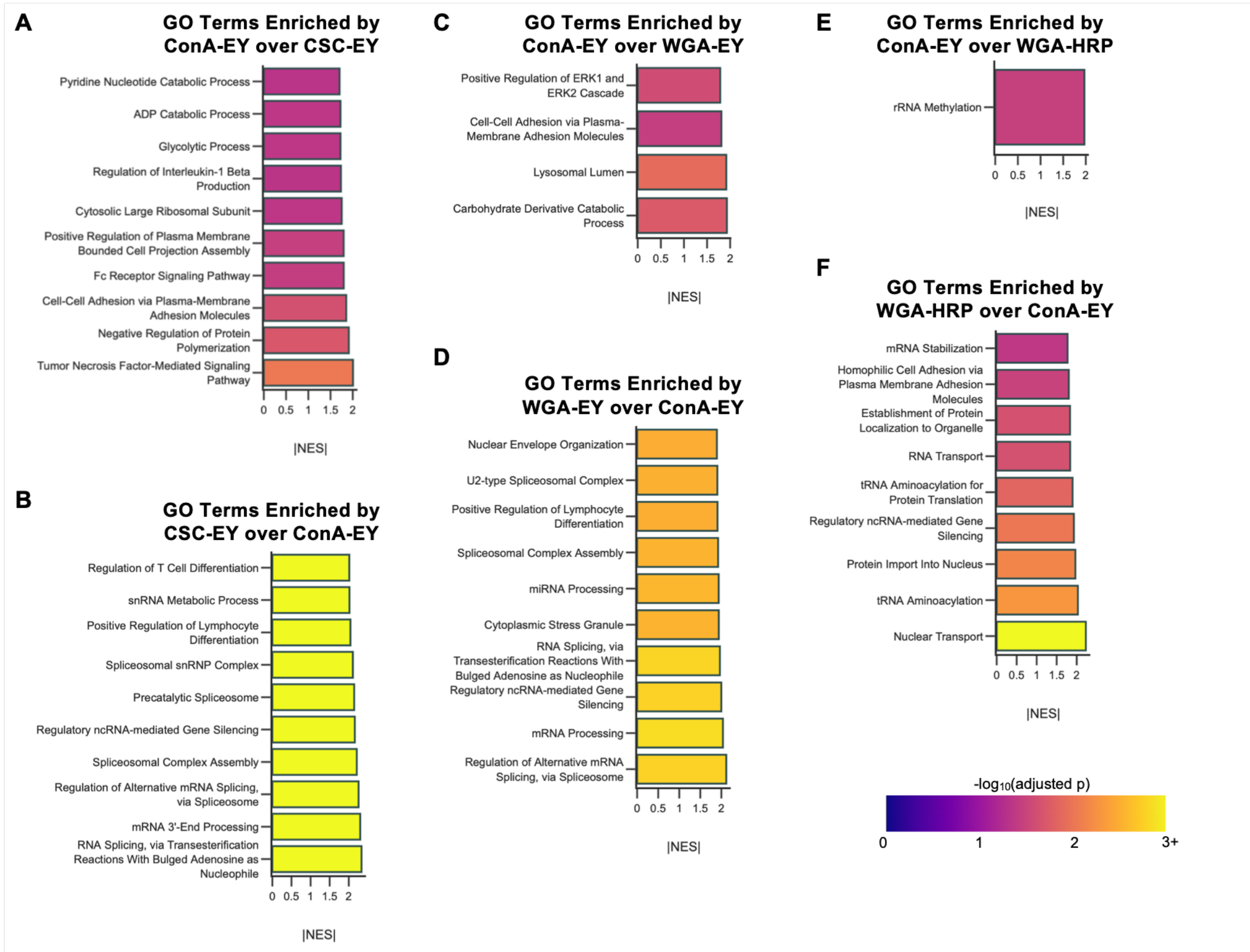

Fig. S47. Ribosomal, RNA, and spliceosome annotations are enriched by CSC-EY, WGA-EY, and WGA-HRP relative to ConA-EY . Endogenous biotin normalized intensities were compared between ConA-EY and other glycan-targeting diffusive surfaceomics methods for GSEA. The top terms (maximum 10; all shown if less than 10) enriched with FDR < 0.05 and ranked by normalized enrichment score (NES) magnitude greater than 1.5 are shown. Note the abundance of canonically intracellular annotations with proteins known to have targetable PTMs relevant for CSC-EY, WGA-EY, and WGA-HRP.

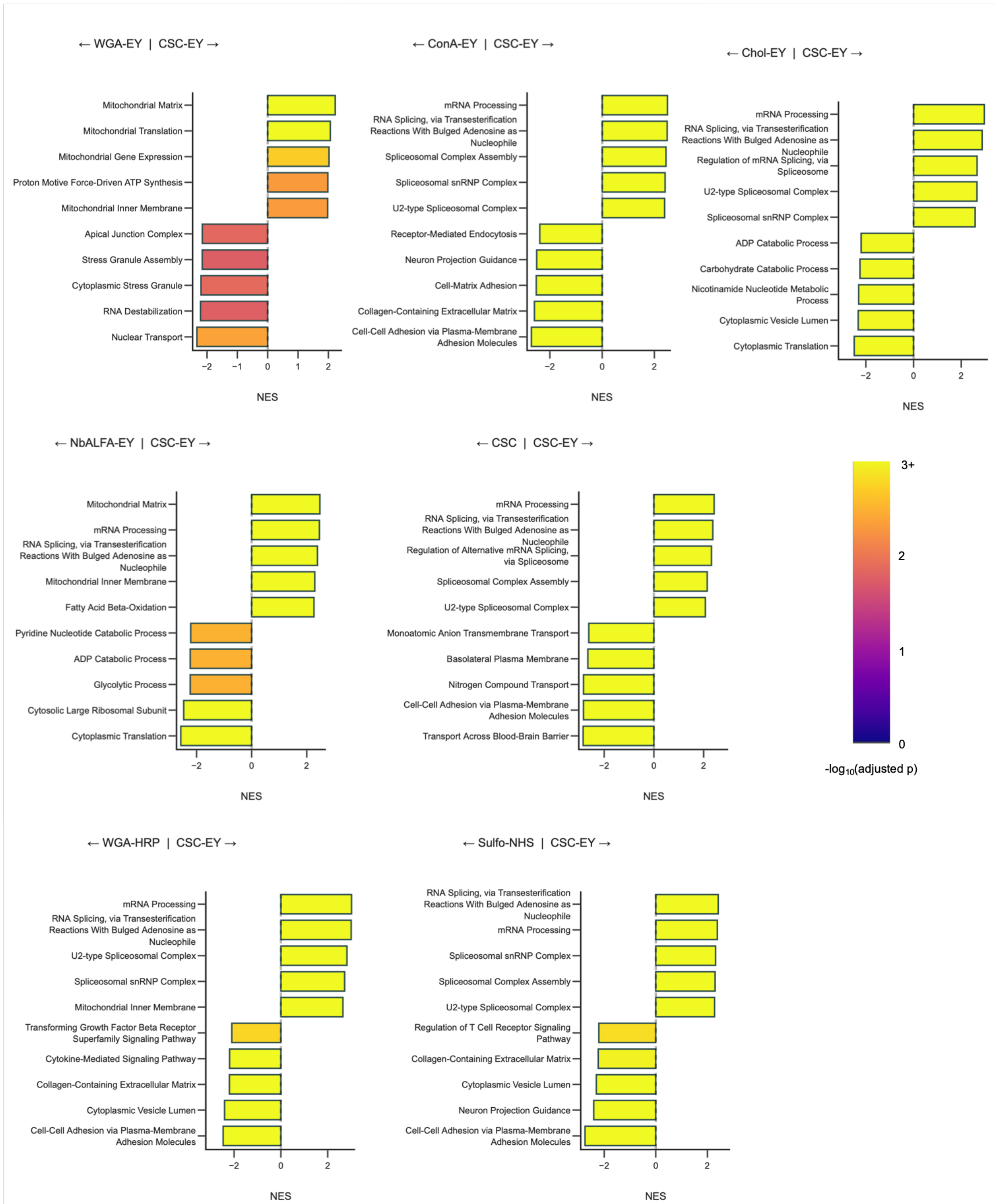

Fig. S48. Gene set enrichment analysis comparisons of surfaceomics methods to CSC-EY. Median normalized intensities for all surfaceomics methods were compared pairwise by GSEA to highlight differences in distribution irrespective of biotinylation efficiency. The top terms (maximum top five in each direction) enriched with FDR < 0.05 and ranked by normalized enrichment score (NES) magnitude greater than 1.5 are shown. CSC-EY enriched terms are on the right.

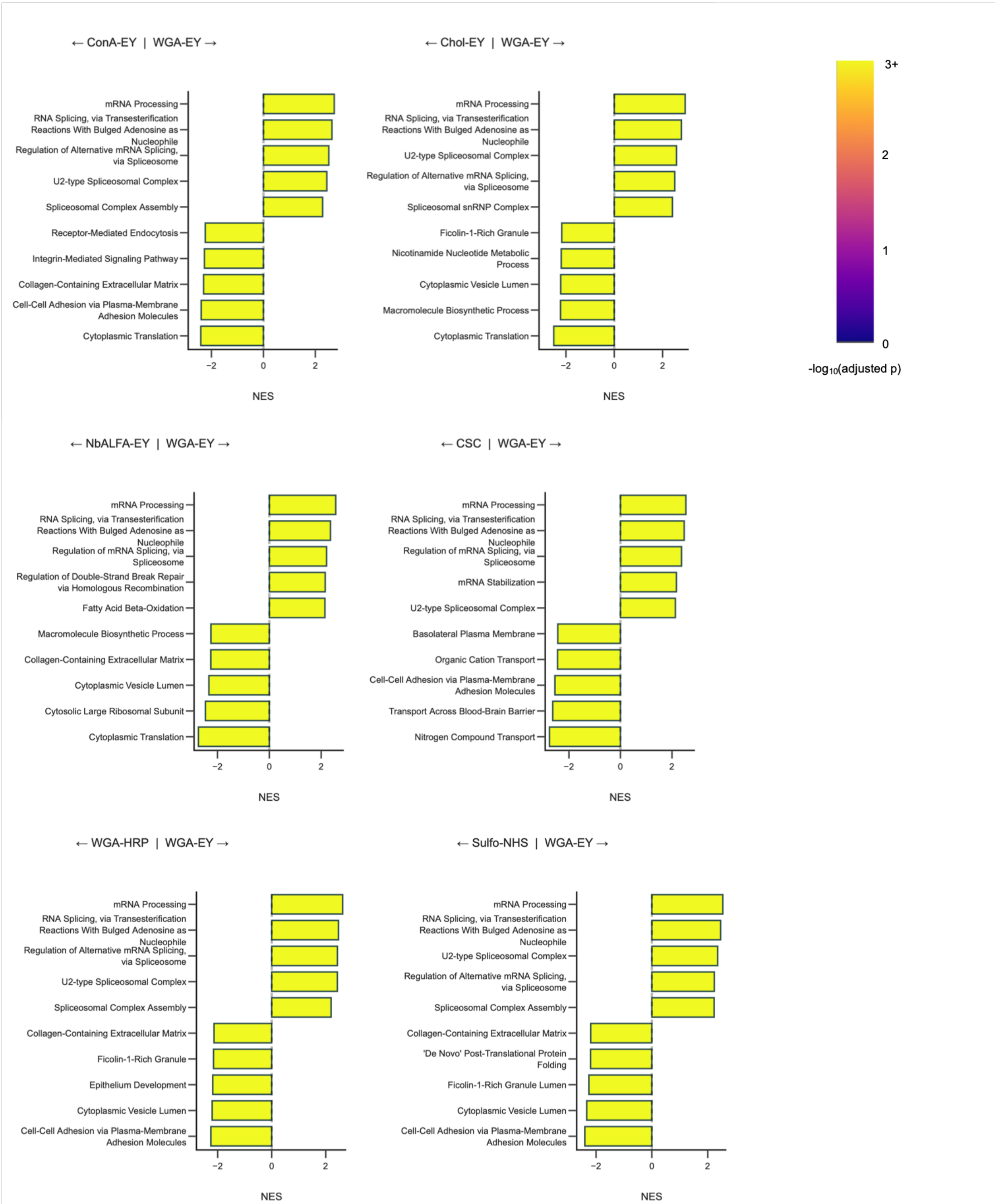

Fig. S49. Gene set enrichment analysis comparisons of surfaceomics methods to WGA-EY. Median normalized intensities for all surfaceomics methods were compared pairwise by GSEA to highlight differences in distribution irrespective of biotinylation efficiency. The top terms (maximum top five in each direction) enriched with FDR < 0.05 and ranked by normalized enrichment score (NES) magnitude greater than 1.5 are shown. WGA-EY enriched terms are on the right.

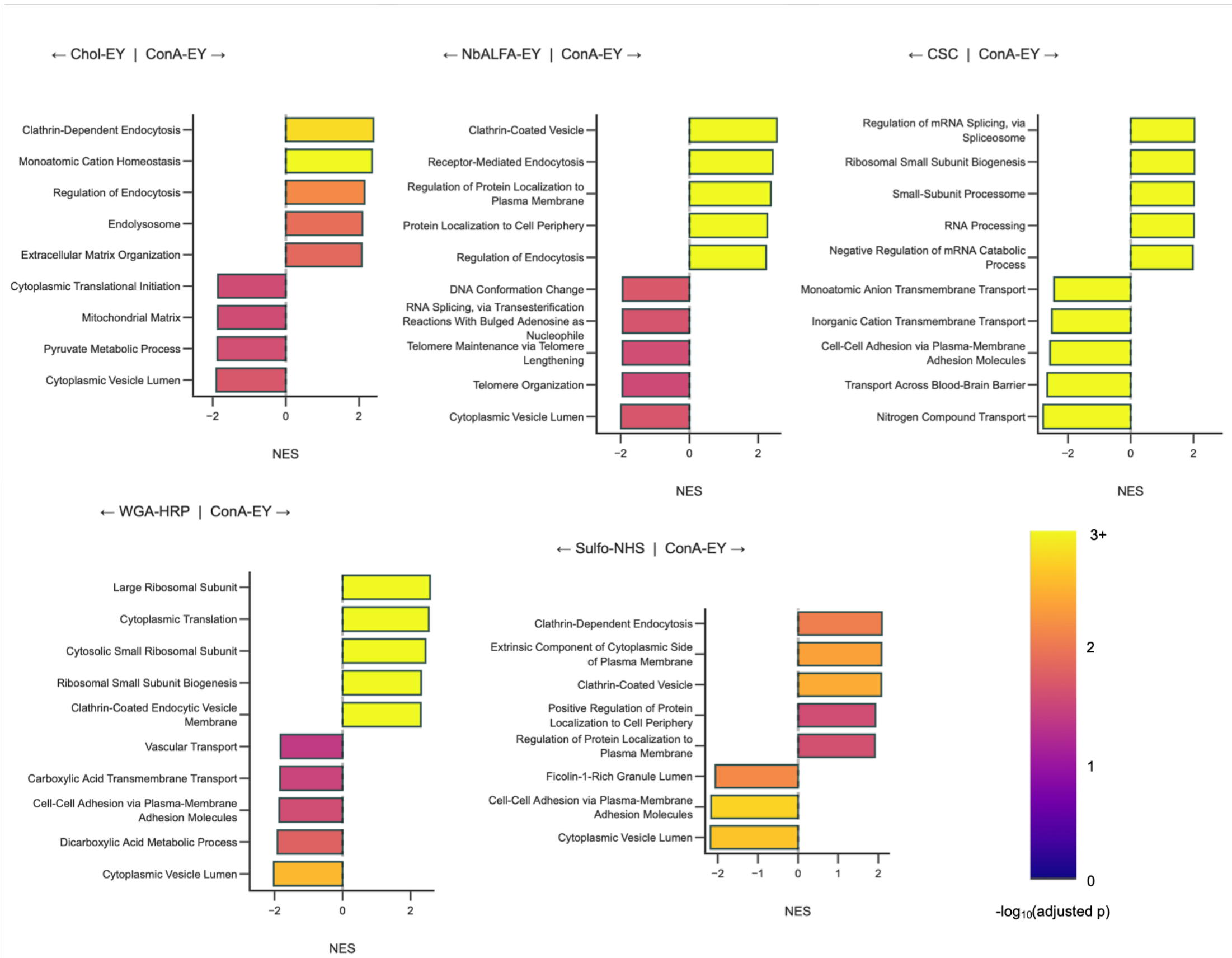

Fig. S50. Gene set enrichment analysis comparisons of surfaceomics methods to ConA-EY. Median normalized intensities for all surfaceomics methods were compared pairwise by GSEA to highlight differences in distribution irrespective of biotinylation efficiency. The top terms (maximum top five in each direction) enriched with FDR < 0.05 and ranked by normalized enrichment score (NES) magnitude greater than 1.5 are shown. ConA-EY enriched terms are on the right.

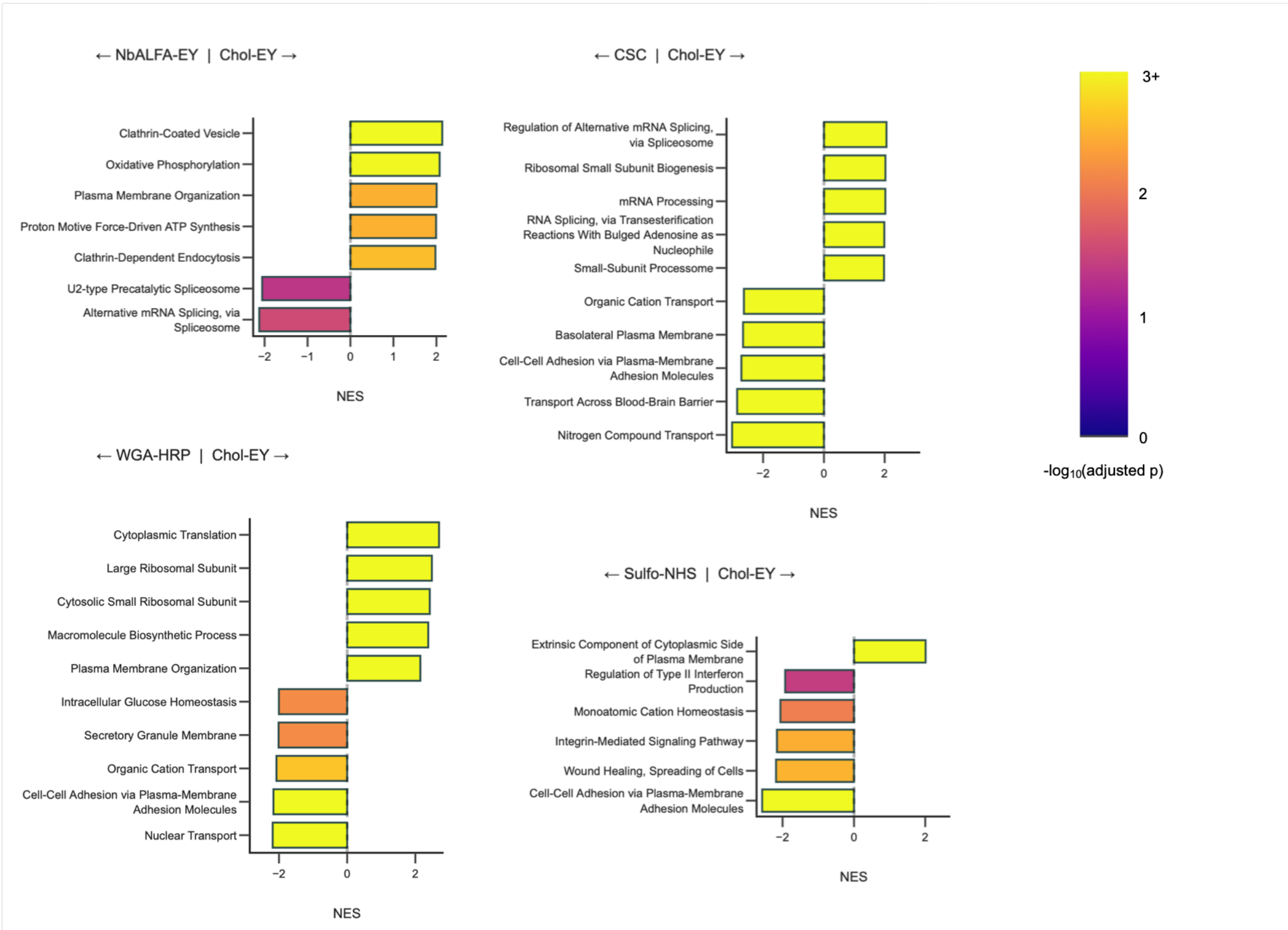

Fig. S51. Gene set enrichment analysis comparisons of surfaceomics methods to Chol-EY. Median normalized intensities for all surfaceomics methods were compared pairwise by GSEA to highlight differences in distribution irrespective of biotinylation efficiency. The top terms (maximum top five in each direction) enriched with FDR < 0.05 and ranked by normalized enrichment score (NES) magnitude greater than 1.5 are shown. Chol-EY enriched terms are on the right.

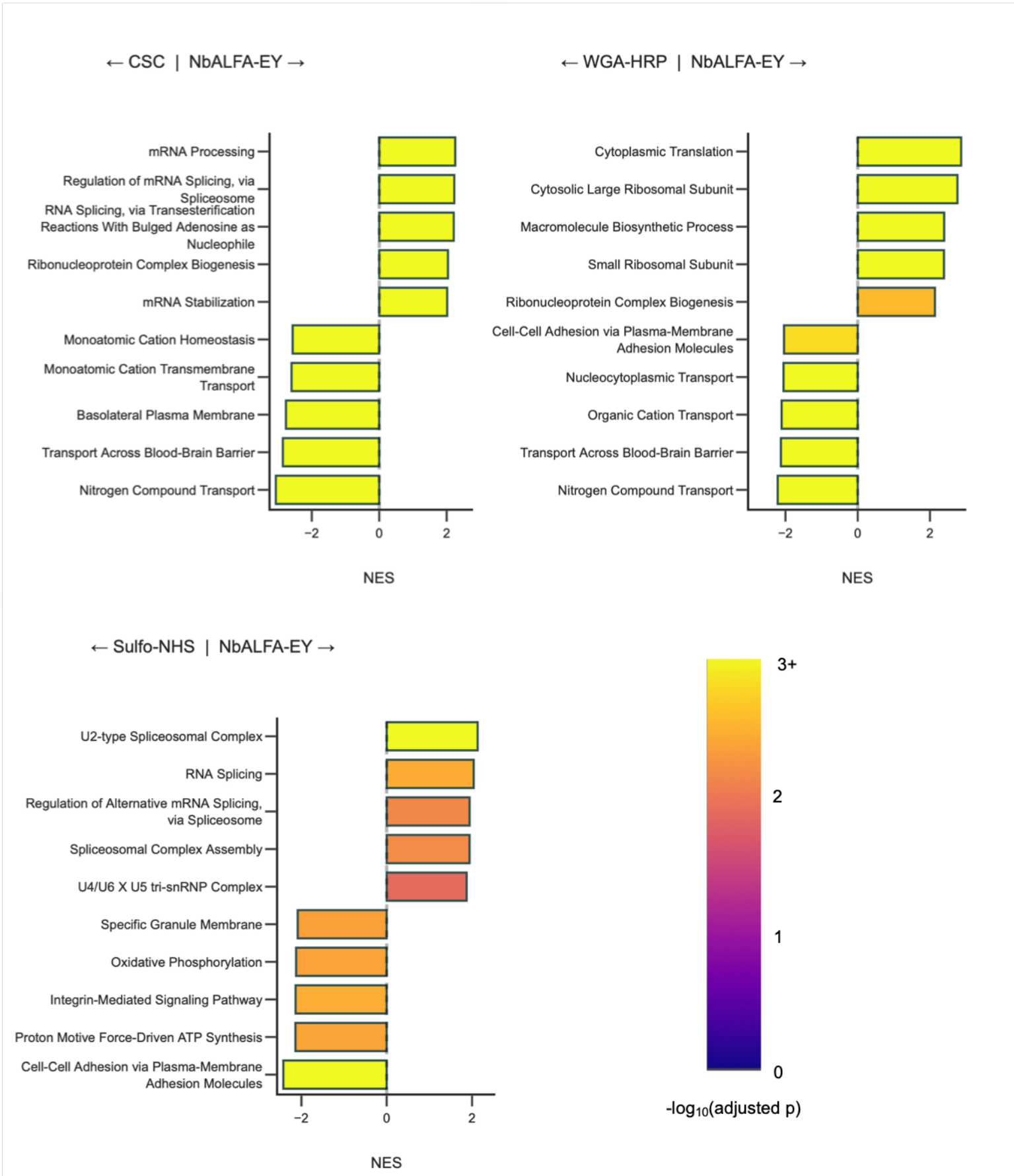

Fig. S52. Gene set enrichment analysis comparisons of surfaceomics methods to NbALFA-EY. Median normalized intensities for all surfaceomics methods were compared pairwise by GSEA to highlight differences in distribution irrespective of biotinylation efficiency. The top terms (maximum top five in each direction) enriched with FDR < 0.05 and ranked by normalized enrichment score (NES) magnitude greater than 1.5 are shown. NbALFA-EY enriched terms are on the right.

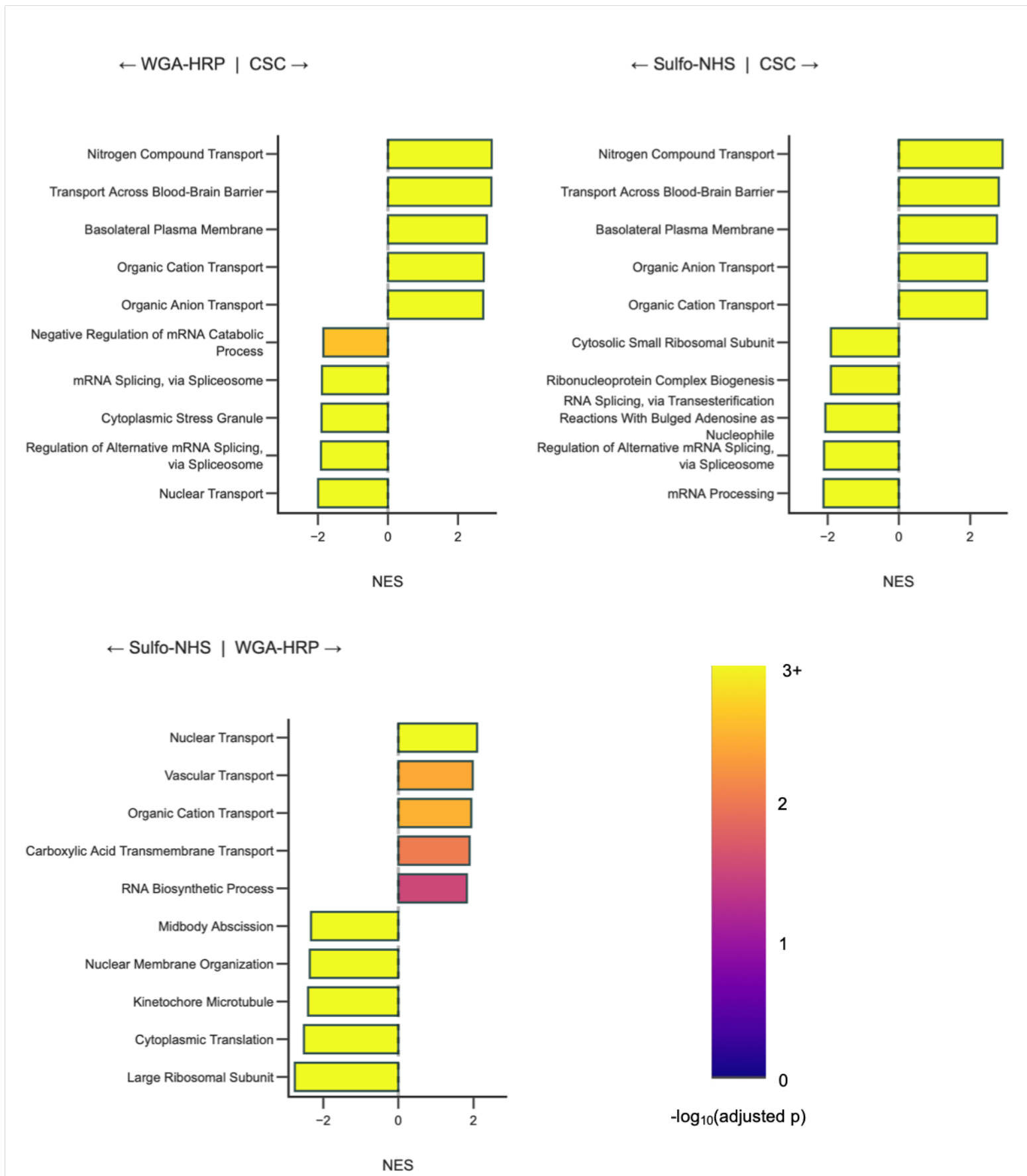

Fig. S53. Gene set enrichment analysis comparisons among literature reported surfaceomics methods. Median normalized intensities for all surfaceomics methods were compared pairwise by GSEA to highlight differences in distribution irrespective of biotinylation efficiency. The top terms (maximum top five in each direction) enriched with FDR < 0.05 and ranked by normalized enrichment score (NES) magnitude greater than 1.5 are shown.

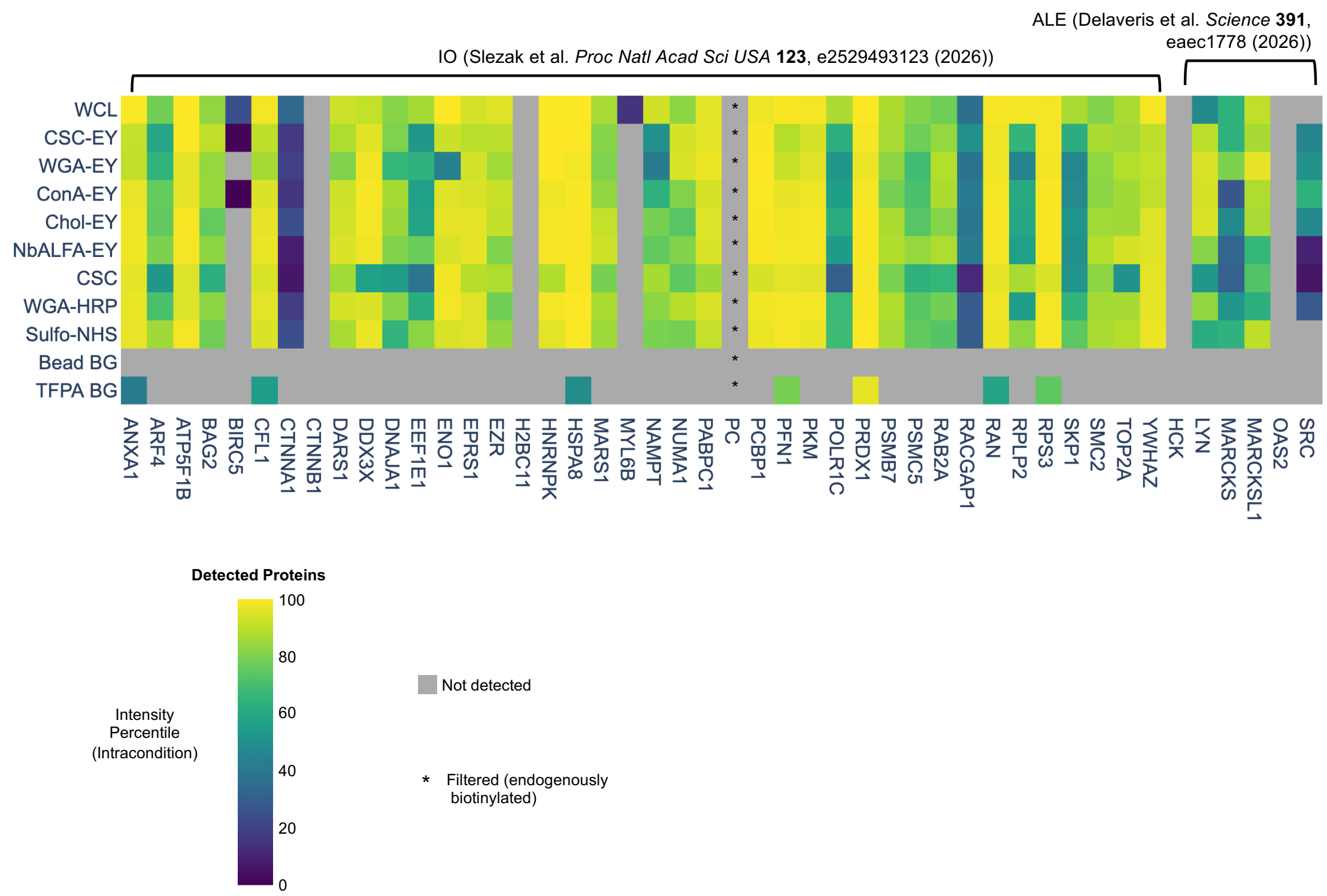

Fig. S54. Noncanonical IO and ALE proteins are consistently detected by surfaceomics. Previously described IO and ALE proteins are consistently detected across surfaceomics methods. Detected proteins are shown with Viridis coloring indicative of the percentile of each protein's intensity within that condition. PGs that did not pass the detection criteria are shown in grey. The IO protein pyruvate carboxylase is endogenously biotinylated and filtered because surfaceomics-induced biotinylation cannot be distinguished from endogenous biotinylation in this experiment.

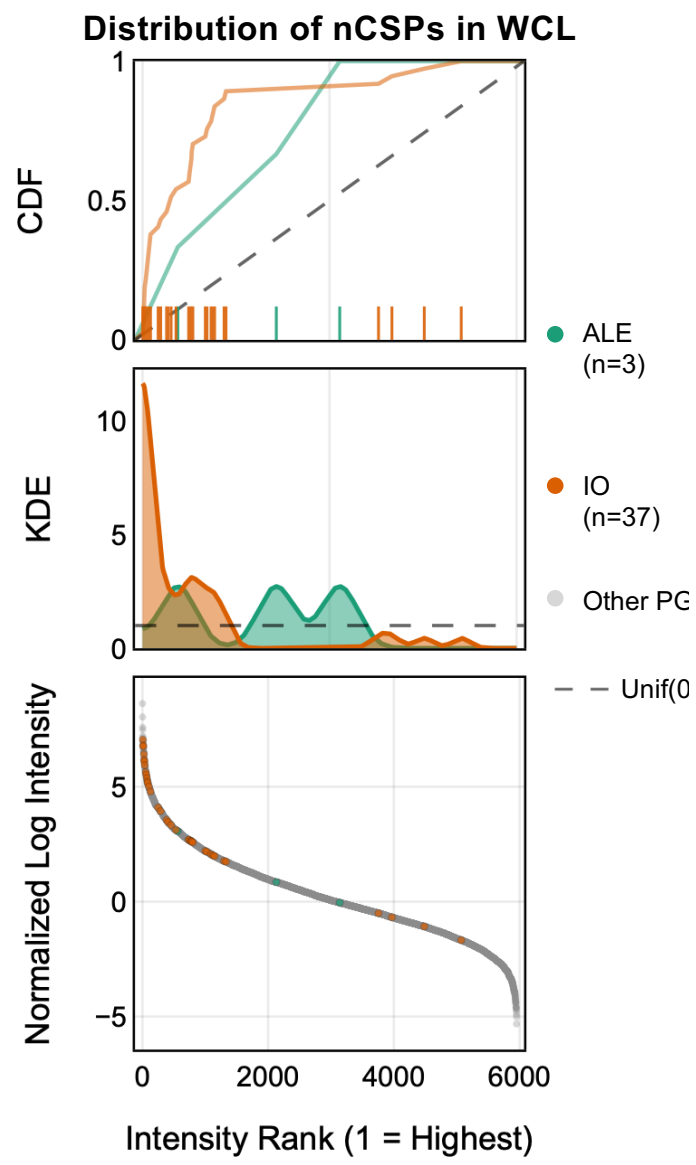

Fig. S55. Noncanonical IO and ALE proteins distribute across a wide intensity range in the whole cell proteome. Known nCSPs distribute across a range of intensities in unenriched whole cell lysate. **Top panel:** Empirical cumulative distribution function (CDF) of IO (orange) and ALE (green) proteins across intensity rank. Tick marks denote location of each individual protein over rank. **Center panel:** Kernel density estimate (KDE) of IO and ALE proteins across intensity rank. **Bottom panel:** Waterfall plot showing the intensity distribution of proteins in WCL, with IO and ALE proteins highlighted. Intensity measurements reflect the mean of biologic triplicate, normalized to endogenous biotin intensity.

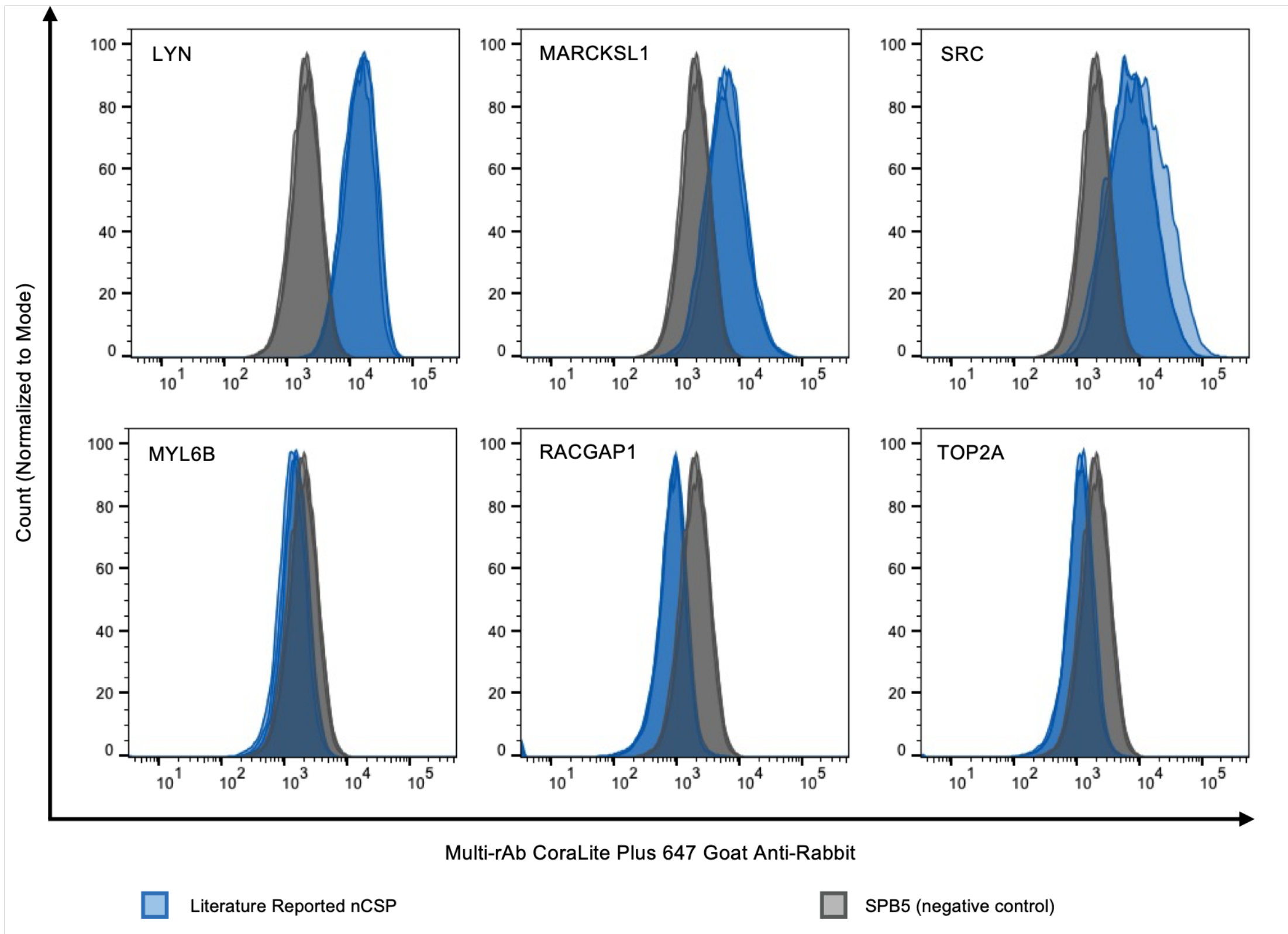

Fig. S56. Commercial antibodies detect canonically intracellular myristoylated proteins at the cell surface by flow cytometry. Flow cytometry histograms of live, nonpermeabilized K562 cells stained with primaries against previously published nCSPs. SYTOX Blue was used to gate out dead or membrane-compromised cells. These histograms correspond to the data used in **Fig. 5C**. Data are reflective of biologic triplicate.

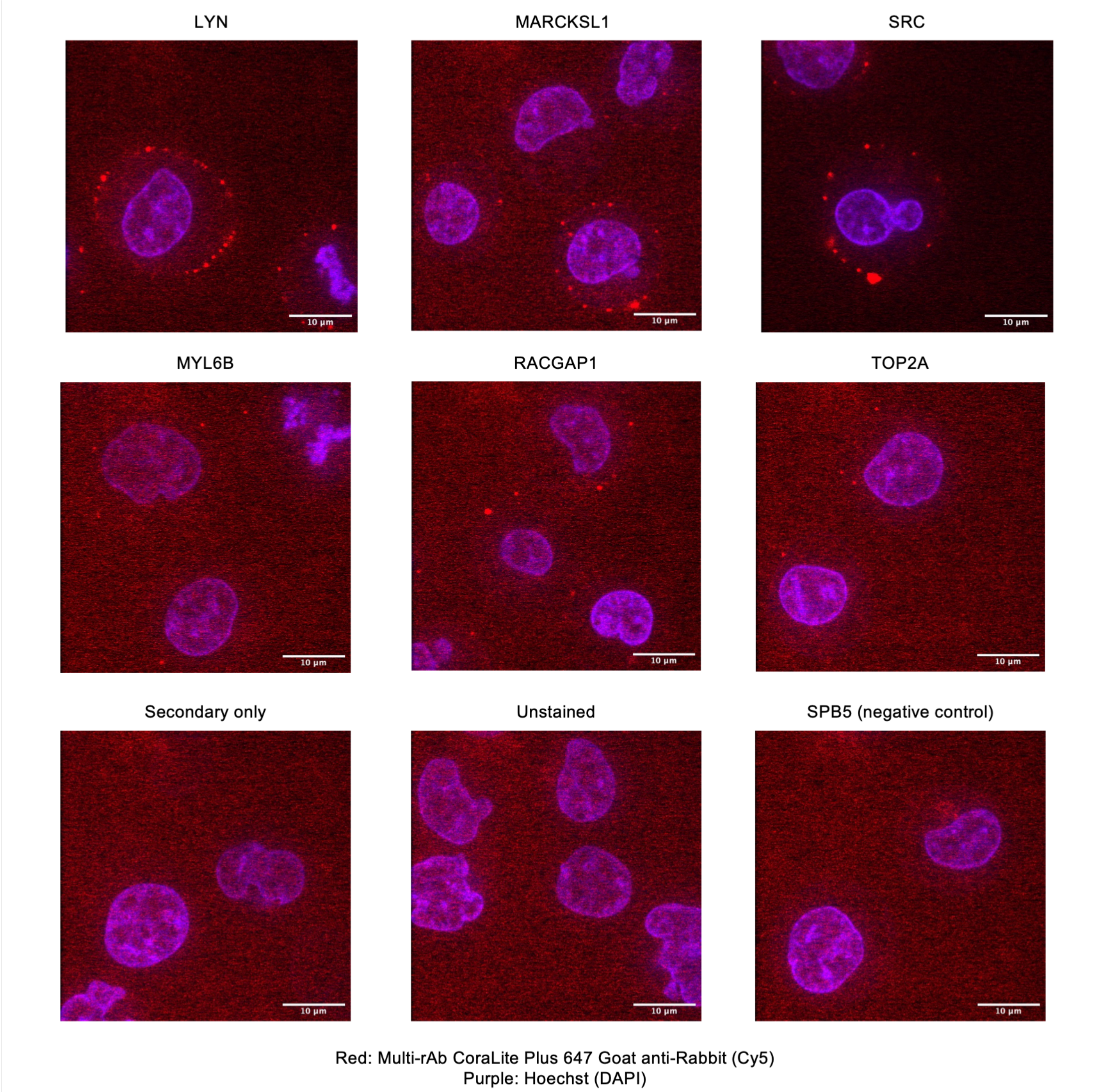

Fig. S57. Commercial antibodies detect canonically intracellular myristoylated proteins at the cell surface by confocal microscopy. Confocal micrographs of nonpermeabilized K562 cells stained with primaries against previously published IO and ALE proteins. A live/dead stain was used to confirm that the cells shown were not dead or permeable. Brightness and contrast were adjusted in each channel per image independently to highlight distribution. Scale bar 10 µm.

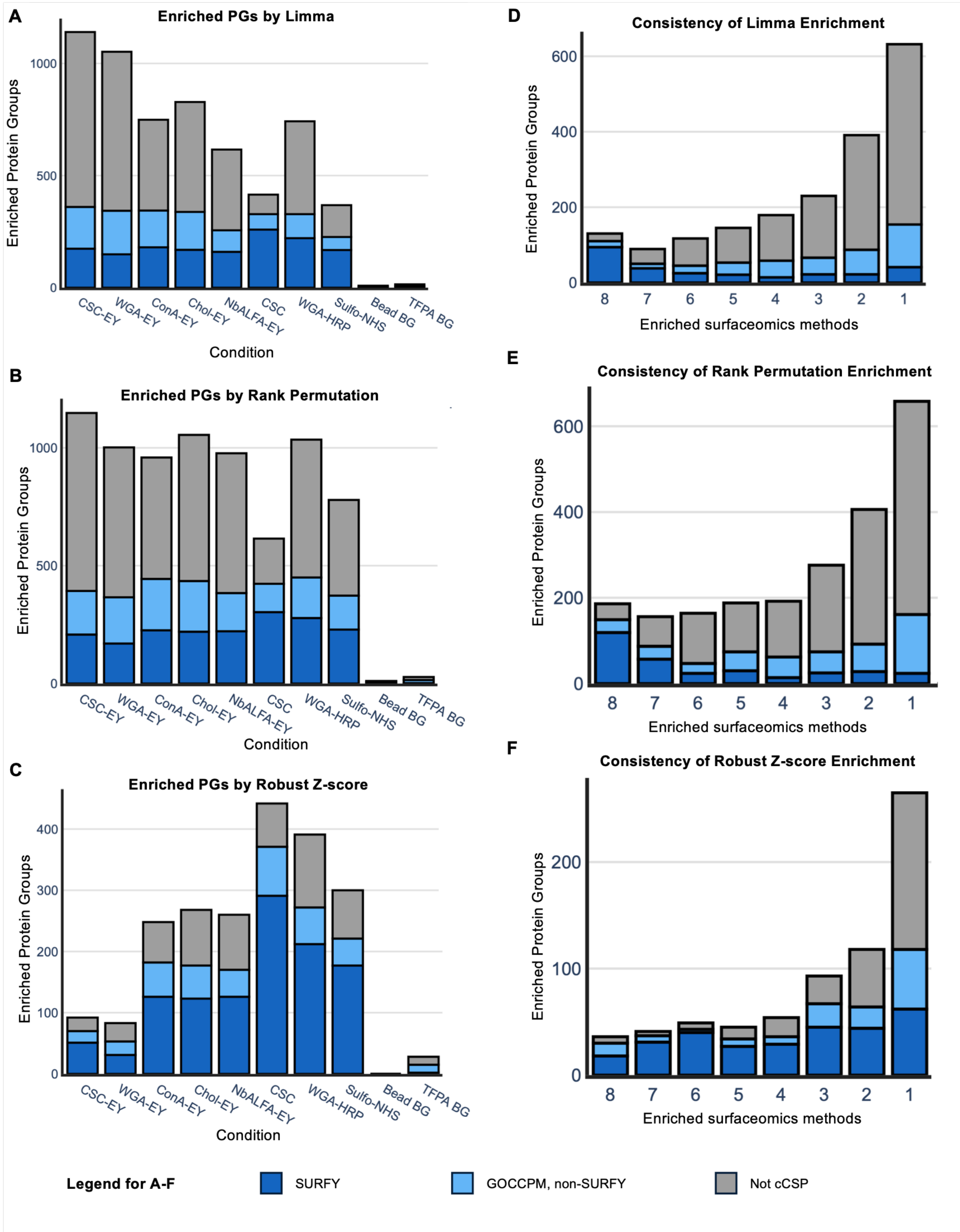

Fig. S58. Enrichment thresholds highlight cCSP enrichment and assist prioritization of nCSP candidates. (**A**) Restricting from detected PGs to PGs passing a limma enrichment threshold of $\log_2$(fold change) ≥ 1 and adjusted $p < 0.05$ when compared to WCL by median normalized values yields a cCSP-heavy population. (**B**) Rank permutation filtering for FDR < 0.05 with comparison to WCL reduces hit number less aggressively than limma, retaining larger non-cCSP populations, but overall recapitulates the trends of number of enriched PGs across surfaceomics methods. (**C**) Robust z-score comparison of surfaceomics methods to WCL with z*

> 2 threshold notably reduces the number of hits and increases cCSP fraction metric. With this filter, CSC enriches the most PGs with other literature reported methods also enriching many PGs. (**D**) PGs consistently enriched by surfaceomics methods according to limma thresholds are largely cCSPs, although there are multiple non-cCSPs. (**E**) Rank permutation enrichment filtering shows similar trends to limma for consistent PG enrichment across surfaceomics methods, although slightly more lenient. (**F**) PGs consistently detected by multiple surfaceomics methods that pass the robust z-score enrichment filter are predominantly cCSPs, although there are still minor populations of non-cCSPs. Robust z is the strictest of the three filters.

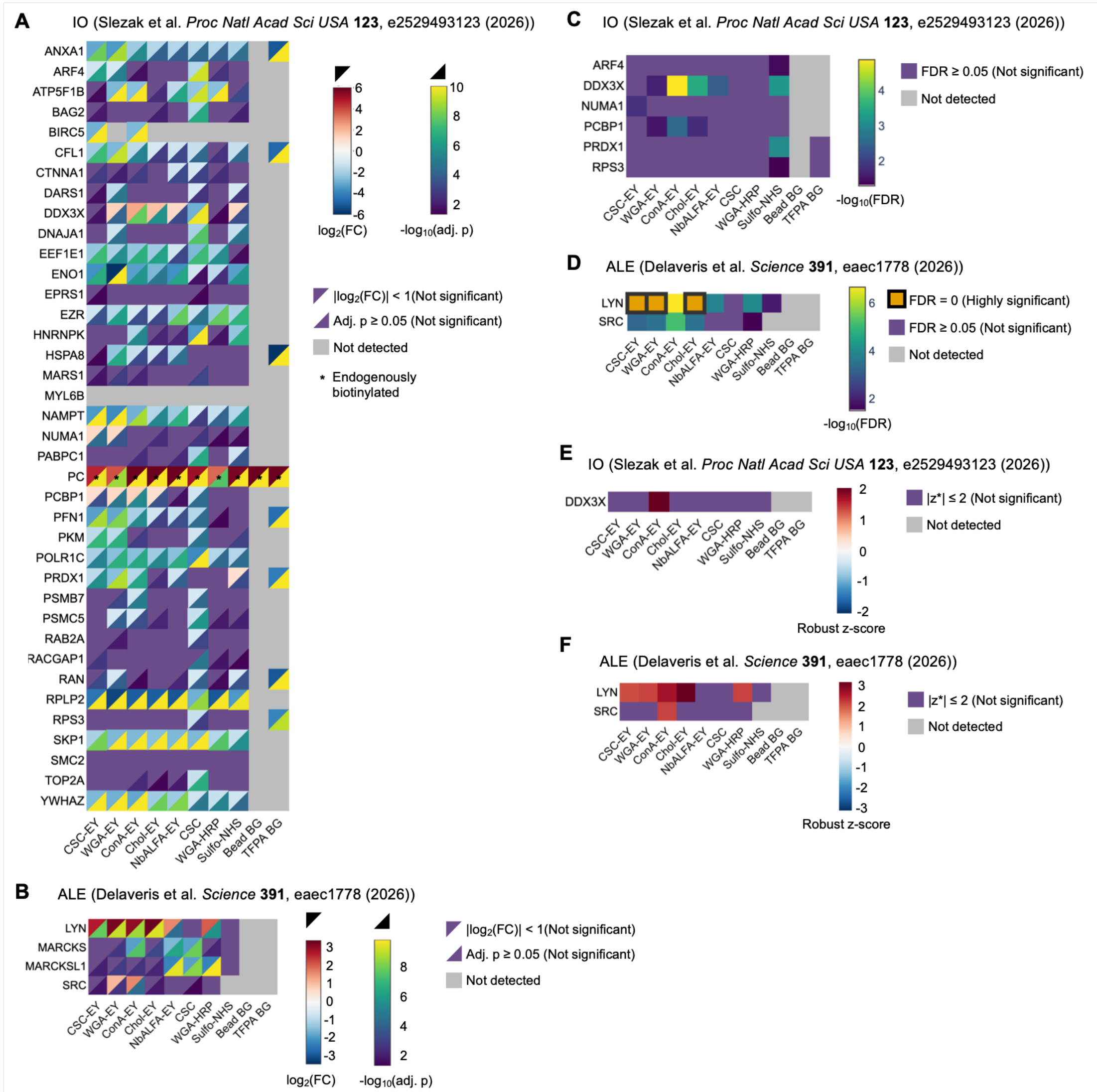

Fig. S59: Enrichment thresholds applied to literature reported nCSPs. (**A**) Limma enrichment signatures of reported IO proteins, showing their relative enrichment to whole cell lysate by median normalized intensities. The top left of each square corresponds to $\log_2$(fold change), and the bottom right corresponds to $-\log_{10}$(adjusted p-value). Light purple indicates that the protein was detected but did not pass the significance threshold in that respective category (($\log_2$(fold change) ≥ 1 or adjusted p < 0.05)). Grey indicates that a protein was not detected in a condition. Note that many IO proteins are higher abundance in WCL than surfaceomics, i.e. they are not enriched by surfaceomics presumably due to multicompartment localization and abundant cytoplasmic populations. (**B**) Limma enrichment signatures of reported ALE proteins. Notably, LYN and SRC that showed strong cell surface validation have significant enrichment by photo-PLP. (**C**) Rank permutation enrichment signatures of IO proteins. Only IO proteins that achieved enrichment significance in at least once condition are shown. Light purple indicates that the protein was detected but did not pass the significance threshold of FDR < 0.05. Grey indicates that a protein was not detected in a condition. (**D**) Rank permutation enrichment signatures of ALE proteins. Only LYN and SRC are shown because MARCKS and MARCKSL1 did not achieve enrichment significance in any condition by the rank permutation method. (**E**) Robust z-score enrichment signatures of DDX3X, the only IO protein to appear as enriched by robust z-score in

any surfaceomics method. Light purple indicates that the protein was detected but did not pass the significance threshold of $z^* > 2$. (**F**) LYN and SRC achieve significance by robust z-score.

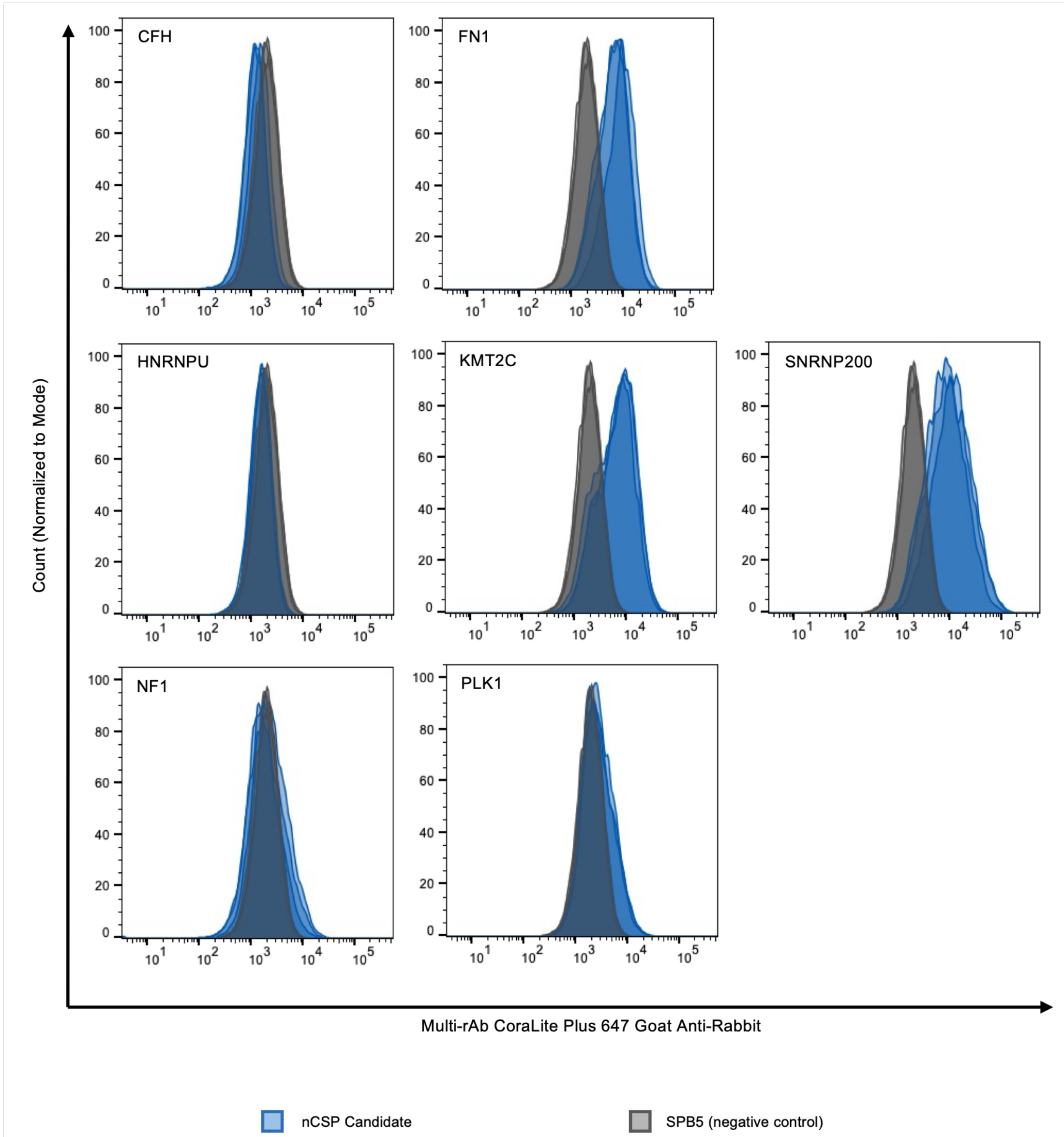

Fig. S60. Commercial antibodies detect CSP candidates at the cell surface by flow cytometry. Flow cytometry histograms of live, nonpermeabilized K562 cells stained with primaries against CSP candidates enriched by surfaceomics. SYTOX Blue was used to gate out dead or membrane-compromised cells. Of the seven tested proteins, FN1, KMT2C, and SNRNP200 showed noticeable surface staining. These histograms correspond to the data used in **Fig. 6D**. Data are reflective of biologic triplicate.

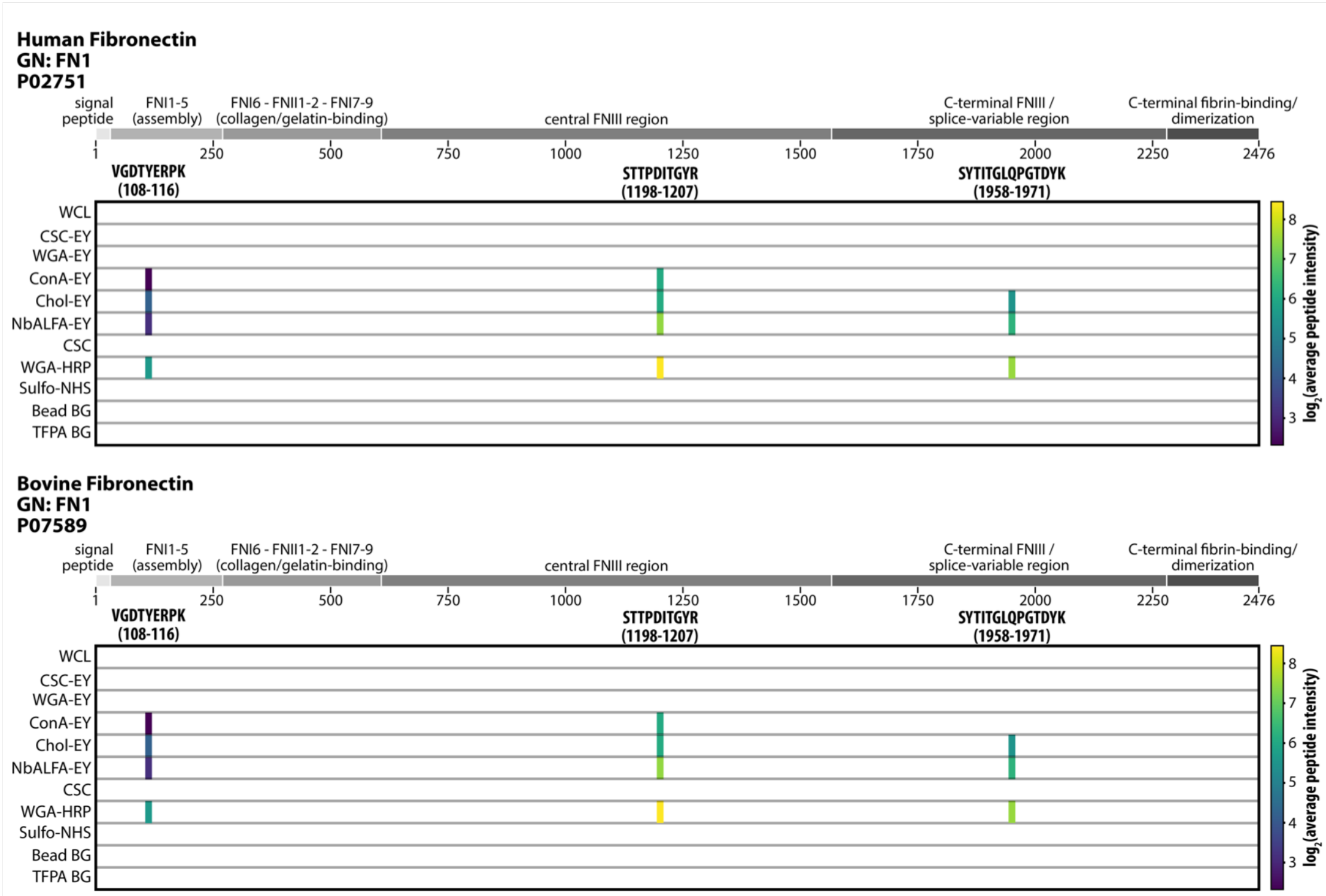

Fig. S61. Mass spectrometry does not distinguish the origin of cell surface FN1. Domains of FN1 from human and cow are shown. Peptides recovered in the mass spectrometry are shown with $\log_2$ average raw intensity. The detected peptides are identical in human and cow, as well as retaining the same residue numbers resulting from high homology.

Tables

Table S1. Enrichment of cCSPs and CIRFESS TM PGs by surfaceomics over WCL by limma

	CSC-EY	WGA-EY	ConA-EY	Chol-EY	NbALFA-EY	CSC	WGA-HRP	Sulfo-NHS
**Total enriched over WCL**	1155	1067	784	848	658	447	763	414
**SURFY enriched over WCL, #**	174	149	182	169	160	261	223	170
**SURFY enriched over WCL, % of all enriched**	15.1	14.0	23.2	19.9	24.3	58.4	29.2	41.1
**GOCCPM enriched over WCL, #**	344	329	342	326	253	308	314	227
**GOCCPM enriched over WCL, % of all enriched**	29.8	30.8	43.6	38.4	38.5	68.9	41.2	54.8
**cCSP enriched over WCL, #**	363	345	355	341	267	335	332	239
**cCSP enriched over WCL, % of all enriched**	31.4	32.3	45.3	40.2	40.6	74.9	43.5	57.7
**CIRFESS TM enriched over WCL, #**	440	395	342	370	304	381	401	260
**CIRFESS TM enriched over WCL, % of all enriched**	38.1	37.0	43.6	43.6	46.2	85.2	52.6	62.80

Table S2. Enrichment of cCSPs and CIRFESS TM PGs by surfaceomics over Bead BG by limma

	CSC-EY	WGA-EY	ConA-EY	Chol-EY	NbALFA-EY	CSC	WGA-HRP	Sulfo-NHS
**Total enriched over Bead BG**	1707	1356	1241	1566	1328	698	1579	1094
**SURFY enriched over Bead BG, #**	79	67	89	102	84	147	133	92
**SURFY enriched over Bead BG, % of all enriched**	4.6	4.9	7.2	6.5	6.3	21.1	8.4	8.4
**GOCCPM enriched over Bead BG, #**	367	300	323	379	302	272	402	293
**GOCCPM enriched over Bead BG, % of all enriched**	21.5	22.1	26.0	24.2	22.7	39.0	25.5	26.8
**cCSP enriched over Bead BG, #**	374	306	332	389	311	282	413	301
**cCSP enriched over Bead BG, % of all enriched**	21.9	22.6	26.8	24.8	23.4	40.4	26.2	27.5
**CIRFESS TM enriched over Bead BG, #**	369	301	285	342	278	252	358	244
**CIRFESS TM enriched over Bead BG, % of all enriched**	21.6	22.2	23.0	21.8	20.9	36.1	22.7	22.3

Table S3. Enrichment of cCSPs and CIRFESS TM PGs by surfaceomics over TFPA BG by limma

	CSC-EY	WGA-EY	ConA-EY	Chol-EY	NbALFA-EY	CSC*	WGA-HRP*	Sulfo-NHS*
**Total enriched over TFPA BG**	1145	917	751	949	738	485	1014	677
**SURFY enriched over TFPA BG, #**	63	51	70	64	50	119	95	72
**SURFY enriched over TFPA BG, % of all enriched**	5.5	5.6	9.3	6.7	6.8	24.54	9.4	10.6
**GOCCPM enriched over TFPA BG, #**	243	207	202	229	166	192	262	185
**GOCCPM enriched over TFPA BG, % of all enriched**	21.2	22.6	26.9	24.1	22.5	39.6	25.8	27.3
**cCSP enriched over TFPA BG, #**	247	211	209	234	171	200	270	191
**cCSP enriched over TFPA BG, % of all enriched**	21.6	23.0	27.8	24.7	23.2	41.2	26.6	28.2
**CIRFESS TM enriched over TFPA BG, #**	240	195	168	188	138	184	221	159
**CIRFESS TM enriched over TFPA BG, % of all enriched**	21.0	21.3	22.4	19.8	18.7	37.9	21.8	23.5

*TFPA BG is not an appropriate control for CSC, WGA-HRP, and Sulfo-NHS because they do not use TFPA-biotin or light. It is shown for these surfaceomics methods strictly for comprehensive comparison.

SI References

1. J. Sung, J. R. Hawkins, A highly sensitive internally-controlled real-time PCR assay for mycoplasma detection in cell cultures. *Biologicals* **64**, 58–72 (2020).

2. Z. Lin, *et al.*, Multiscale photocatalytic proximity labeling reveals cell surface neighbors on and between cells. *Science* **385**, eadl5763 (2024).

3. S. K. Elledge, *et al.*, Systematic identification of engineered methionines and oxaziridines for efficient, stable, and site-specific antibody bioconjugation. *Proceedings of the National Academy of Sciences* **117**, 5733–5740 (2020).

4. E. G. Bligh, W. J. Dyer, A rapid method of total lipid extraction and purification. *Can. J. Biochem. Physiol.* **37**, 911–917 (1959).

5. C. J. Bracken, *et al.*, Bi-paratopic and multivalent VH domains block ACE2 binding and neutralize SARS-CoV-2. *Nat Chem Biol* **17**, 113–121 (2021).

6. D. Z. Dieters-Castator, *et al.*, Magnetic Bead-Based Workflow for Sensitive and Streamlined Cell Surface Proteomics. *J. Proteome Res.* **23**, 618–632 (2024).

7. L. L. Kirkemo, *et al.*, Cell-surface tethered promiscuous biotinylators enable comparative small-scale surface proteomic analysis of human extracellular vesicles and cells. *eLife* **11**, e73982 (2022).

8. T. G. Bartholow, *et al.*, Photoproximity Labeling from Single Catalyst Sites Allows Calibration and Increased Resolution for Carbene Labeling of Protein Partners In Vitro and on Cells. *ACS Cent. Sci.* **10**, 199–208 (2024).

9. J. B. Geri, *et al.*, Microenvironment mapping via Dexter energy transfer on immune cells. *Science* **367**, 1091–1097 (2020).

10. S. W. Huth, J. B. Geri, J. V. Oakley, D. W. C. MacMillan, μMap-Interface: Temporal Photoproximity Labeling Identifies F11R as a Functional Member of the Transient Phagocytic Surfaceome. *J. Am. Chem. Soc.* (2024). https://doi.org/10.1021/jacs.4c11058.

11. D. Wessel, U. I. Flügge, A method for the quantitative recovery of protein in dilute solution in the presence of detergents and lipids. *Analytical Biochemistry* **138**, 141–143 (1984).

12. A. P. Martin, *et al.*, A spatiotemporal Notch interaction map from plasma membrane to nucleus. *Science Signaling* **16**, eadg6474 (2023).

13. K. K. Leung, K. Schaefer, Z. Lin, Z. Yao, J. A. Wells, Engineered Proteins and Chemical Tools to Probe the Cell Surface Proteome. *Chem. Rev.* (2025). https://doi.org/10.1021/acs.chemrev.4c00554.

14. R. Bruderer, *et al.*, Extending the Limits of Quantitative Proteome Profiling with Data-Independent Acquisition and Application to Acetaminophen-Treated Three-Dimensional Liver Microtissues*[S]. *Molecular & Cellular Proteomics* **14**, 1400–1410 (2015).

15. The UniProt Consortium, UniProt: the Universal Protein Knowledgebase in 2025. *Nucleic Acids Res* **53**, D609–D617 (2025).

16. A. M. Frankenfield, J. Ni, M. Ahmed, L. Hao, Protein Contaminants Matter: Building Universal Protein Contaminant Libraries for DDA and DIA Proteomics. *J. Proteome Res.* **21**, 2104–2113 (2022).

17. D. Bausch-Fluck, *et al.*, A Mass Spectrometric-Derived Cell Surface Protein Atlas. *PLOS ONE* **10**, e0121314 (2015).

18. T. G. O. Consortium, *et al.*, The Gene Ontology knowledgebase in 2026. *Nucleic Acids Res* **54**, D1779–D1792 (2026).

19. M. Ashburner, *et al.*, Gene Ontology: tool for the unification of biology. *Nat Genet* **25**, 25–29 (2000).

20. S. Tyanova, *et al.*, The Perseus computational platform for comprehensive analysis of (prote)omics data. *Nat Methods* **13**, 731–740 (2016).

21. F. Pedregosa, *et al.*, Scikit-learn: Machine Learning in Python. *Journal of Machine Learning Research* **12**, 2825–2830 (2011).

22. H. L. P. Tytgat, *et al.*, Endogenous biotin-binding proteins: an overlooked factor causing false positives in streptavidin-based protein detection. *Microb Biotechnol* **8**, 164–168 (2015).

23. M. Ingaramo, D. Beckett, Selectivity in Post-translational Biotin Addition to Five Human Carboxylases *. *Journal of Biological Chemistry* **287**, 1813–1822 (2012).

24. M. E. Ritchie, *et al.*, limma powers differential expression analyses for RNA-sequencing and microarray studies. *Nucleic Acids Res* **43**, e47 (2015).

25. Y. Benjamini, Y. Hochberg, Controlling the False Discovery Rate: A Practical and Powerful Approach to Multiple Testing. *Royal Statistical Society. Journal. Series B: Methodological* **57**, 289–300 (1995).

26. Z. Fang, X. Liu, G. Peltz, GSEApy: a comprehensive package for performing gene set enrichment analysis in Python. *Bioinformatics* **39**, btac757 (2023).

27. G. Yu, Gene Ontology Semantic Similarity Analysis Using GOSemSim. *Methods Mol Biol* **2117**, 207–215 (2020).

28. G. Yu, *et al.*, GOSemSim: an R package for measuring semantic similarity among GO terms and gene products. *Bioinformatics* **26**, 976–978 (2010).

29. M. Milacic, *et al.*, The Reactome Pathway Knowledgebase 2024. *Nucleic Acids Res* **52**, D672–D678 (2024).

30. M. Kanehisa, M. Furumichi, Y. Sato, Y. Matsuura, M. Ishiguro-Watanabe, KEGG: biological systems database as a model of the real world. *Nucleic Acids Res* **53**, D672–D677 (2025).

31. M. Kanehisa, S. Goto, KEGG: Kyoto Encyclopedia of Genes and Genomes. *Nucleic Acids Res* **28**, 27–30 (2000).

32. F. Hong, *et al.*, RankProd: a bioconductor package for detecting differentially expressed genes in meta-analysis. *Bioinformatics* **22**, 2825–2827 (2006).

33. C. S. Wright, 2·2 Å resolution structure analysis of two refined *N*-acetylneuraminyl-lactose—Wheat germ agglutinin isolectin complexes. *Journal of Molecular Biology* **215**, 635–651 (1990).

34. D. N. Moothoo, J. H. Naismith, Concanavalin A distorts the β-GlcNAc-(1→2)-Man linkage of β-GlcNAc-(1→2)-α-Man-(1→3)-[β-GlcNAc-(1→2)-α-Man-(1→6)]-Man upon binding. *Glycobiology* **8**, 173–181 (1998).

35. J. Abramson, *et al.*, Accurate structure prediction of biomolecular interactions with AlphaFold 3. *Nature* **630**, 493–500 (2024).

36. E. C. Meng, *et al.*, UCSF ChimeraX: Tools for structure building and analysis. *Protein Science* **32**, e4792 (2023).
